# Supplementary material for: Impact of review method on the conclusions of clinical reviews: A systematic review on dietary interventions in depression as a case in point
Source: PLoS One. 2020 Sep 16;15(9):e0238131. doi: 10.1371/journal.pone.0238131 (PMC7494108; doi:10.1371/journal.pone.0238131)
Supplement: S1 Text — (DOCX) [file pone.0238131.s002.docx]

**Search strategy (30^th^ May, 2020)**

**PubMed:** 1779 hits (primary search)

(((("diet"[MeSH Terms] OR "diet"[All Fields]) OR "food"[MeSH Terms]) OR "diet therapy"[MeSH Terms]) AND ((("depressive*"[All Fields] OR (((((((((((("depressed"[All Fields] OR "depression"[MeSH Terms]) OR "depression"[All Fields]) OR "depressions"[All Fields]) OR "depression s"[All Fields]) OR "depressive disorder"[MeSH Terms]) OR ("depressive"[All Fields] AND "disorder"[All Fields])) OR "depressive disorder"[All Fields]) OR "depressivity"[All Fields]) OR "depressive"[All Fields]) OR "depressively"[All Fields]) OR "depressiveness"[All Fields]) OR "depressives"[All Fields])) OR "mental health"[MeSH Terms]) OR "mental disorder*"[Title/Abstract])) AND (("systematic review"[Publication Type] OR "meta-analysis"[Publication Type]) OR "review"[Publication Type])

**Web of Science Core Collection:** 1353 (84 unique)

(TS=(diet OR food) AND TS=("depressive*" OR depression OR "mental health" OR "mental disorder*") AND TS=("systematic review" OR meta-analysis OR review)) AND DOCUMENT TYPES: (Review)

Indexes=SCI-EXPANDED, SSCI, A&HCI, ESCI Timespan=All years

**Cochrane Database of Systematic Reviews:** 55 hits (0 unique)

ID Search Hits

#1 ((diet OR food) AND (depression OR depressive* OR "mental disorder*" OR "mental health")):ti,ab,kw

in Cochrane Reviews (Word variations have been searched)

**Google Scholar:** 2 unique papers were found by a general search in google scholar using a broad range of keywords related to reviews on the topic of diet and depression.
