# Supplementary material for: Impact of review method on the conclusions of clinical reviews: A systematic review on dietary interventions in depression as a case in point
Source: PLoS One. 2020 Sep 16;15(9):e0238131. doi: 10.1371/journal.pone.0238131 (PMC7494108; doi:10.1371/journal.pone.0238131)
Supplement: S2 Text — (DOCX) [file pone.0238131.s003.docx]

**Meta-analysis of RCTs**

**Specification of errors in the Firth et al. (2019) meta-analysis**

To assess whether narrative reviews overestimated the effects of a diet on depression outcome, we aimed to judge the certainty of the evidence in this field using the GRADE approach. We aimed to do this using a high-quality meta-analysis of randomized controlled trials (RCTs) – the highest level of evidence. However, while assessing the meta-analysis by Firth *et al.* (2019)^1^ on the effects of dietary improvement on depression, two errors were noted. First, Firth *et al.* (2019) included the study by Endevelt *et al.* (2011)^2^ although it did not meet all inclusion criteria, as given in the paper. This study compared two dietary interventions, while the eligibility criteria by Firth *et al.* (2019) specify that only *non-dietary conditions* could serve as a control (page 266). Second, from the Wardle *et al.* (2000)^3^ study, Firth *et al.* (2019) extracted the effects size (Hedge’s *g*) of 1.68 (95% CI = 1.36 to 2.01; *P* < 0.0001). However, Wardle *et al.* (2000), report that there were *no significant between-group changes* (page 551). We estimated the effect size that can be derived from Wardle *et al.* (2000) to be around 0.25 (*P* > 0.132).

All errors are in the direction of the study hypothesis of the Firth *et al.* (2019) study. Their meta-analysis reports on 16 studies, of which nine report a significant benefit of the dietary intervention over the control condition (*i.e.,* a mix of active and non-active control conditions). Among these nine significant studies is Endevelt *et al.* (2011). The extracted effect size from Wardle *et al.* (2000) is six to seven times larger in the analysis by Firth *et al.* (2019) relative to the actual effect size, in favor of the dietary condition. These errors distort the results to such a worrisome extent that we decided to exclude this study from further analyses. Results from subgroup analyses and assessment of publication bias also are reported erroneously by Firth *et al.* (2019). The latter the authors have confirmed, yet appropriate action (*i.e.,* a full and transparent correction) by the editor (Professor Kop of Tilburg University, The Netherlands) of the Journal where the paper is published, *Psychosomatic Medicine*, has been not been taken yet.

As an alternative, and in line with our protocol, we conducted a corrected and updated version of the meta-analysis.

**Details on the method and results of our performed meta-analysis of RCTs**

Systematic searches yielded a total of 17 papers reporting RCTs on the effects of interventions aimed at improving diet on depressive symptoms (see Table 5 in the manuscript). Further information on study selection and exclusion and study characteristics of included studies are provided in Fig A and Tables A to D. Information about the methodological quality of the included studies is provided in Tables E and F. A GRADE evaluation of this meta-analysis can be found in the S9 Appendix.

Some of the included studies aimed to *prevent* depression or depressive symptoms from occurring by means of dietary improvement and included (at-risk) participants without clinical depressive symptoms. Other studies aimed to *treat* patients by means of a diet and, hence, included participants with clinical or subclinical depressive symptoms. A third type of study aimed to find an *association* between diet and depressive symptoms over time, without the specific aim to treat or prevent depression. Pooled effect-size estimates were calculated for these three types of studies: prevention studies, treatment studies, and association studies. Results are presented in Fig B and Table 5 of the main text. In sum, dietary interventions did not outperform control conditions in preventing or treating depression. In association studies only, dietary interventions outperformed control conditions in reducing depression (*g* = -0.14, 95% CI = -0.24 to -0.04). Between-study heterogeneity (*I*^2^ = 49%), however, casts doubt on the nature of this association. Furthermore, the effect-size that was obtained is far from being clinically significant. Note that these results need to be interpreted carefully since they are only based on 15 effect-size estimates.

Directly replicating the Firth *et al.* (2019) meta-analysis with relevant and correct data favored the dietary interventions over the control conditions (*g* = -0.07, 95% CI = -0.10 to -0.04, *P* = 0.04). The observed effect-size, however, was significantly lower (-0.07 versus -0.28). Note that the lack of effect for treatment and prevention studies was not due to potent effects by the control conditions (see Table D), as these, in general, were not able to yield substantial reductions in depressive symptoms over time (pooled effect-size estimate for all control conditions, *g* = -0.14, 95% CI = -0.27 to 0.02). Given that the effect size estimate of pill-placebo on depressive symptoms is > 0.5, the control conditions used in this field may not be perceived as being adequate control conditions accounting, for instance, for expectancy effects and other general treatment effects.^4^

**Differences in method and approach from the Firth et al. (2019) meta-analysis**

In our meta-analysis, we decided to deviate from the method by Firth *et al.* (2019) with regard to the following:

1. The final search date (December 3^rd^, 2018) was extended to August 3^rd^, 2019.
2. Articles were also eligible when they were written in Dutch French, German, or Spanish, and not only in English. This was done to reduce language bias.
3. We excluded Endevelt *et al.* (2011) because this study does not meet an exclusion criterion.
4. The error in the effect-size estimate of Wardle *et al.* (2000) was corrected.
5. Methodological quality assessment of included trials was performed using: (I) the tool recommended by the Cochrane library and (II) the tool recommended by the U.S. Department of Health and Human Services. We did not use the ADA evidence process, as was used by Firth *et al.* (2019). Quality assessment according to the ADA process is rather similar to the one that is recommended by the U.S. Department of Health and Human Services, except for questions about *relevance*, which in our view are rather subjective and not informative with regard to the methodological quality of an RCT.
6. Our primary sub-group analyses differed from those that were used by Firth *et al.* (2019). Our subgroup analyses were based on whether a study aimed to: (I) *prevent* depression or depressive symptoms from occurring (*i.e.,* studies that included participants without clinical depressive symptoms), (II) *treat* patients by means of a diet (*i.e.,* studies that at baseline included participants with clinical or subclinical depressive symptoms), and (III) find an *association* between diet and depressive symptoms over time, without the specific aim to treat or prevent depression, and in which having depressive symptoms or being completely free of depressive symptoms were not an exclusion criteria at baseline. Studies that included *individuals at risk* were considered prevention studies. The reason for choosing subgroups analyses based on whether a study is a treatment, prevention, or an association trial was based on the GRADE approach that we applied. The GRADE approach requires that specific questions are formulated regarding population, intervention, control, and outcome measure. Besides, in case effect sizes were reported separately for men and women (e.g., Kiernan *et al.,* 2001), we chose to use them as such and not to pool them into a single estimate.
7. In the SMILES trial, by Jacka *et al.* (2017)^5^, the interviewer-based improvement was much higher than the self-reported improvement in the dietary condition (*i.e.,* a difference with effect size, Cohen’s *d* of 0.8). This difference was not observed in the control condition (*d* = 0.0).^6^ This suggests a loss of blinding of the persons who conducted the assessments, which could be seen as an indication of bias. Hence, we used the self-reported outcomes of this study (standardized difference in means = ~ -0.62).
8. We only took depression/depressive symptoms as an outcome and, hence, did not use the search term "anxiety" that was used by Firth *et al*. (2019). Besides, we find the search term “Random Allocation” used by Firth *et al.* (2019) redundant. Hence, we excluded it from our search string. Because of this, the results from our systematic search is also somewhat different. Note that our simplified search did not return fewer eligible hits.

*Supplemental methods and results*

Electronic searches were performed using the following search string: (“Diet” OR “Mediterranean” OR “Therapy” OR “Educat*” OR “Counsel*” OR “Intervention*” OR “Treatment*”) AND (“Randomized Controlled Trial” or “Clinical Trial” or “Control Groups”) AND (“Depression” or “Depressive Disord*”). This search, together with alternative search methods that were performed until August 3^rd^, 2019 (see Firth *et al.,* 2019) returned a total of 6,220 candidate articles.

## Identification

6,183 records identified through databases PUBMED, PsycINFO, EMBASE, Web of Science, and Google Scholar

37 records identified through reference lists and grey literature

## Included

17 studies included in quantitative synthesis (= 18 effect sizes):

Prevention ^a^ studies: 2

Treatment ^b^ studies: 4

Association ^c^ studies: 12

## Eligibility

49 records excluded

Reasons for exclusion are shown in Table A

66 full-text records assessed for eligibility

4,527 records screened after duplicates removed

4,461 records excluded

## Screening

**Fig A. Flowchart on identification, screening, and inclusion of eligible articles.**

^a^ *Prevention* = the study starts with a group of non-clinically depressed individuals and a diet is tested for its depression preventive properties.

^b^ *Treatment* = the study starts with a group of (clinically)-depressed individuals and a diet is tested for its anti-depressant properties.

^c^ *Association* = the diet is associated with a change in symptoms over time.

| **Table A.** Reasons for in- or exclusion after full-text assessment (in chronological and alphabetically order). | | | |
| --- | --- | --- | --- |
| **Author, year** | **Type of trial** | **In- vs. excluded** | **Reason** |
| Rosen *et al.* (1982)^7^ | Prevention (RCT) | Excluded | Two diet-related conditions |
| Lloyd *et al.* (1996)^8^ | Association (RCT) | Excluded | Three diet-related conditions |
| Benton *et al.* (1995)^9^ | Association (RCT) | Excluded | Exposure to a nutraceutical, not diet |
| Wells *et al.* (1998)^10^ | Prevention (RCT) | Excluded | Two diet-related conditions |
| Nieman *et al.* (2000)^11^ | Association (RCT) | Included | Meets inclusion criteria |
| Wardle *et al.* (2000)^3^ | Association (RCT) | Included | Meets inclusion criteria |
| Kiernan *et al.* (2001)^12^ | Association (RCT) | Included | Meets inclusion criteria |
| Hyyppä *et al.* (2003)^13^ | Association (RCT) | Included | Meets inclusion criteria |
| Ness *et al.* (2003)^14^ | Prevention (RCT) | Excluded | Exposure to fish |
| Scheier *et al.* (2005)^15^ | Association (RCT) | Included | Meets inclusion criteria |
| Halyburton *et al.* (2007)^16^ | Association (RCT) | Excluded | Two diet-related conditions |
| Merrill *et al.* (2008)^17^ | Prevention (RCT) | Excluded | No pure dietary exposure |
| Brinkworth *et al.* (2009)^18^ | Association (RCT) | Excluded | Two diet-related conditions |
| Cheatham *et al.* (2009)^19^ | Association (RCT) | Excluded | Two diet-related conditions |
| Jenkinson *et al.* (2009)^20^ | Association (RCT) | Included | Meets inclusion criteria |
| Einvik *et al.* (2010)^21^ | Association (RCT) | Included | Meets inclusion criteria |
| Thomson *et al.* (2010)^22^ | Association (RCT) | Excluded | Three diet-related conditions |
| Endevelt *et al.* (2011)^2^ | Association (RCT) | Excluded | No suitable control condition |
| McMillan *et al.* (2011)^23^ | Association (RCT) | Included | Meets inclusion criteria, no active control |
| Ciarambino *et al.* (2011)^24^ | Association (RCT) | Excluded | Two diet-related conditions |
| Imayama *et al.* (2011)^25^ | Association (RCT) | Included | Meets inclusion criteria |
| Beezhold & Johnston (2012)^26^ | Association (RCT) | Excluded | Three diet-related conditions |
| Casañas *et al.* (2012)^27^ | Association (RCT) | Excluded | No pure dietary exposure |
| Ciarambino *et al.* (2012)^28^ | Association (RCT) | Excluded | Two diet-related conditions |
| Forster *et al.* (2012)^29^ | Association (RCT) | Included | Meets inclusion criteria |
| García-Toro *et al.* (2012 a)^30^ | Treatment (RCT) | Excluded | Two (not pure) diet-related conditions |
| García-Toro *et al.* (2012 b)^31^ | No trial | Excluded | Trial protocol |
| Torres & Nowson (2012)^32^ | Prevention (RCT) | Excluded | Two (not pure) diet-related conditions |
| Ruusunen *et al.* (2012)^33^ | Association (RCT) | Excluded | No pure dietary exposure |
| Nijamkin *et al.* (2013)^34^ | Prevention (RCT) | Excluded | No pure dietary exposure |
| Sánchez-Villegas *et al.* (2013)^35^ | Prevention (RCT) | Excluded | Three diet-related conditions |
| Shahnazari *et al.* (2013)^36^ | N.A. | Excluded | No mood/depression assessments |
| Stange *et al.* (2013)^37^ | Prevention (RCT) | Excluded | Dietary supplements, not diet |
| Kasckow *et al.* (2014 a)^38^ | Treatment (RCT) | Included | Meets inclusion criteria |
| Kasckow *et al.* (2014 b)^39^ | Association (RCT) | Included | Meets inclusion criteria |
| Reynolds *et al.* (2014)^40^ | Prevention (RCT) | Excluded | Overlap with Kasckow *et al.* (2014 b) |
| Stahl *et al.* (2014)^41^ | No trial | Excluded | Case study |
| ***Table A*** *continues on next page* | | | |
| ***Table A*** *continued* | | | |
| Agarwal *et al.* (2015)^42^ | Association (RCT) | Included | Meets inclusion criteria, no active control |
| Bartels *et al.* (2015)^43^ | Association (RCT) | Excluded | No pure dietary exposure |
| Forsyth *et al.* (2015)^44^ | Treatment (RCT) | Excluded | No (pure) diet-related conditions |
| Knight *et al.* (2015)^45^ | No trail | Excluded | Trial protocol |
| Melnyk *et al.* (2015)^46^ | Association (RCT) | Excluded | No pure dietary exposure |
| Suominen *et al.* (2015)^47^ | Prevention (RCT) | Excluded | Two diet-related conditions |
| Watson *et al.* (2015)^48^ | Association (RCT) | Excluded | Trial protocol |
| Assaf *et al.* (2016)^49^ | Prevention (RCT) | Excluded | Two diet-related conditions |
| Brinkworth *et al.* (2016)^50^ | Association (RCT) | Excluded | No pure dietary exposure |
| Dodd *et al.* (2016)^51^ | Association (RCT) | Excluded | No pure dietary exposure |
| Moncrieft *et al.* (2016)^52^ | Association (RCT) | Excluded | No pure dietary exposure |
| Roca *et al.* (2016)^53^ | No trail | Excluded | Trial protocol |
| Shomaker *et al.* (2016)^54^ | Treatment (RCT) | Excluded | No (pure) diet-related conditions |
| Webber *et al.* (2016)^55^ | Prevention (RCT) | Excluded | No pure dietary exposure |
| Cicero *et al.* (2017)^56^ | Treatment (RCT) | Excluded | Exposure to a nutraceutical, not diet |
| Jacka *et al.* (2017)^5^ | Treatment (RCT) | Included | Meets inclusion criteria |
| Ng *et al.* (2017)^57^ | Association (RCT) | Excluded | No pure dietary exposure |
| Sharifan *et al.* (2017)^58^ | Prevention (RCT) | Excluded | Exposure to a food group, not diet |
| Stewart *et al.* (2017)^59^ | Prevention (RCT) | Excluded | Exposure to a nutraceutical, not diet |
| Swoboda *et al.* (2017)^60^ | Association (RCT) | Excluded | No (pure) diet-related conditions |
| Young *et al.* (2017)^61^ | Association (RCT) | Excluded | No pure dietary exposure |
| Jamilian *et al.* (2018)^62^ | Treatment (RCT) | Excluded | Exposure to a nutraceutical, not diet |
| Sánchez-Villegas *et al.* (2018)^63^ | Association (RCT) | Excluded | Cross-sectional data only |
| Sarris *et al.* (2018)^64^ | Treatment (RCT) | Excluded | Exposure to a nutraceutical, not diet |
| Berk *et al.* (2019)^65^ | Treatment (RCT) | Excluded | Exposure to a nutraceutical, not diet |
| Bot *et al.* (2019)^66^ | Prevention (RCT) | Included | Meets inclusion criteria |
| Ma *et al.* (2019)^67^ | Treatment (RCT) | Excluded | No pure dietary exposure |
| Nishi *et al.* (2019)^68^ | Treatment (RCT) | Excluded | Exposure to a nutraceutical, not diet |
| Parletta *et al.* (2019)^69^ | Treatment (RCT) | Included | Meets inclusion criteria |

| **Table B.** Study characteristics. | | | | | |
| --- | --- | --- | --- | --- | --- |
| **Author, year** | ***N* _int_** | ***N* _con_** | **Average age** | **% female** | **Country** |
| **Association** |  |  |  |  |  |
| Nieman *et al.* (2000)^11^ ^a^ | 26 | 22 | 44 | 100 | U.S. |
| Nieman *et al.* (2000)^11^ ^b^ | 22 | 21 | 44 | 100 | U.S. |
| Wardle *et al.* (2000)^3 a 1^ | 52 | 50 | 52 | 42 | U.K. |
| Wardle *et al.* (2000)^3^ ^b^ | 53 | 50 | 53 | 56 | U.K. |
| Kiernan *et al.* (2001)^12^ ^a^ | 40 | 40 | +/-39 | 0 | U.S. |
| Kiernan *et al.* (2001)^12^ ^b^ | 31 | 39 | +/-39 | 100 | U.S. |
| Hyyppä *et al.* (2003)^13^ | 60 | 60 | 48 | 0 | Finland |
| Scheier *et al.* (2005)^15^ | 78 | 76 | 44 | 100 | U.S. |
| Jenkinson *et al.* (2009)^20^ | 190 | 126 | 61 | 66 | U.K. |
| Einvik *et al.* (2010)^21^ | 253 | 252 | 70 | 0 | Norway |
| Imayama *et al.* (2011)^25^ ^a^ | 118 | 87 | 58 | 100 | U.S. |
| Imayama *et al.* (2011)^25^ ^b^ | 117 | 117 | 58 | 100 | U.S. |
| McMillan *et al.* (2011)^23^ | 12 | 13 | 21 | 100 | Australia |
| Forster *et al.* (2012)^29^ | 72 | 67 | 73 | 51 | U.K. |
| Agarwal *et al.* (2015)^42^ | 137 | 139 | 44 | 82 | U.S. |
| Assaf *et al.* (2015)^49^ | 17,335 | 25,698 | 65 | 100 | U.S. |
| **Prevention** |  |  |  |  |  |
| Bot *et al.* (2019)^66^ ^a^ | 256 | 257 | 46 | 75 | Various EU countries |
| Bot *et al.* (2019)^66^ ^b^ | 256 | 256 | 47 | 77 | Various EU countries |
| **Treatment** |  |  |  |  |  |
| Kasckow et al. (2014 a)^38^ | 10 | 13 | 23 | 0 | U.S. |
| Kasckow et al. (2014 b)^39^ | 27 | 25 | 63 | 75 | U.S. |
| Jacka *et al.* (2017)^5^ | 31 | 25 | 40 | 72 | Australia |
| Parletta *et al.* (2019)^69^ | 47 | 38 | 44 | 69 | Australia |
| *Note. N* _int_; number of participants in the intervention condition who completed the trial, *N* _con_; number of participants in the control condition who completed the trial.  ^1^ Wardle et al. (2000) reported on 2 dietary conditions (1 low fat condition and 1 Mediterranean diet condition) and 1 control condition. Given that study effect size estimates in a meta-analysis should be independent, we excluded the low-fat condition from further analysis. The Cochrane library recommends this strategy (<https://handbook-5-1.cochrane.org/chapter_16/16_5_4_how_to_include_multiple_groups_from_one_study.htm>; accessed June 10, 2020). Note that both dietary interventions yielded similar results in the Wardle et al. study. Imputing the effect size yielded by the low fat condition in this meta-analysis did not lead to considerable changes in the results reported herein. | | | | | |

| **Table C.** Study characteristics - diagnostics and outcome measurement | | |
| --- | --- | --- |
| **Author, year** | **Diagnosis** | **Outcome measure** |
| **Association** |  |  |
| Nieman *et al.* (2000)^11^ ^a^ | None | Profiles of Mood State, depression |
| Nieman *et al.* (2000)^11^ ^b^ | None | Profiles of Mood State, depression |
| Wardle *et al.* (2000)^3 a^ | None | Beck’s Depression Inventory; Profiles of Mood State, depression |
| Wardle *et al.* (2000)^3^ ^b^ | None | Beck’s Depression Inventory; Profiles of Mood State, depression |
| Kiernan *et al.* (2001)^12^ ^a^ | None | Beck’s Depression Inventory |
| Kiernan *et al.* (2001)^12^ ^b^ | None | Beck’s Depression Inventory |
| Hyyppä *et al.* (2003)^13^ | None | Brief Symptom Inventory depression scale |
| Scheier *et al.* (2005)^15^ | None | ﻿Center for Epidemiological Studies Depression Scale |
| Jenkinson *et al.* (2009)^20^ | None | Hospital Anxiety and Depression Scale, depression |
| Einvik *et al.* (2010)^21^ | None | Hospital Anxiety and Depression Scale, depression |
| Imayama *et al.* (2011)^25^ ^a^ | None | Brief Symptom Inventory 18 items |
| Imayama *et al.* (2011)^25^ ^b^ | None | Brief Symptom Inventory 18 items |
| McMillan *et al.* (2011)^23^ | None | Profiles of Mood State, depression |
| Forster *et al.* (2012)^29^ | None | Geriatric Depression Screening scale |
| Agarwal *et al.* (2015)^42^ | None | Short-form health survey 36 items |
| Assaf *et al.* (2015)^49^ | None | Center for Epidemiological Studies Depression scale |
| **Prevention** |  |  |
| Bot *et al.* (2019)^66^ ^a^ | None | Patient Health Questionnaire |
| Bot *et al.* (2019)^66^ ^b^ | None | Patient Health Questionnaire |
| **Treatment** |  |  |
| Kasckow et al. (2014 a)^38^ | Depressive symptoms | Hamilton Depression Rating Scale |
| Kasckow et al. (2014 b)^39^ | Depressive symptoms | Beck’s Depression Inventory |
| Jacka *et al.* (2017)^5^ | Depression | Hospital Anxiety and Depression Scale, depression |
| Parletta *et al.* (2019)^69^ | Depression | Depression and Anxiety Stress Scale 21 items |

| **Table D.** Study characteristics – intervention and control conditions | | | | | | |  |
| --- | --- | --- | --- | --- | --- | --- | --- |
| **Author, year** | **Type of intervention** | **Control condition** | | | | **Duration** |  |
| **Association** |  |  | | | |  |  |
| Nieman *et al.* (2000)^11^ ^a^ | Dietary advice by dieticians and weekly 45 minutes sessions and calorie restriction | No active control | | | | 12 weeks |  |
| Nieman *et al.* (2000)^11^ ^b^ | Dietary advice by dieticians, weekly 45 minutes sessions, calorie restriction, and physical exercise | Physical exercise | | | | 12 weeks |  |
| Wardle *et al.* (2000)^3 a^ | Low-fat diet (coaching in individual and group sessions with a dietician and a psychologist). The intervention aimed to reduce the intake of fat, in particular, saturated fat | Waiting-list control | | | | 12 weeks |  |
| Wardle *et al.* (2000)^3^ ^b^ | Mediterranean diet (coaching in individual and group sessions with a dietician and a psychologist). The intervention aimed to increase adherence to a typical Mediterranean dietary pattern | Waiting-list control | | | | 12 weeks |  |
| Kiernan *et al.* (2001)^12^ | Dietary advice according to the National cholesterol education program. First 3 months, weekly meetings with a dietician, next 3 months every other week, and each month in the final 6 months | No active control | | | | 12 months |  |
| Hyyppä *et al.* (2003)^13^ | 1 individual session with a dietician, 2 groups counseling sessions and monthly group sessions in which participants were instructed to adhere to a Mediterranean diet | No active control; continue Hessians habitual diet | | | | 12 weeks |  |
| Scheier *et al.* (2005)^15^ | Dietary advice by dietician, 4 monthly 2 hour group sessions, instructed to adhere to a ﻿low-fat, high-fruit-and-vegetable diet | No active control | | | | 13 months |  |
| Jenkinson *et al.* (2009)^20^ | Individual dietary advice and exercise plus individual dietary advice. The dietary advice was in line with healthy eating practices and delivered through home visits (every month the first six months and thereafter every other month) | No active control, leaflet, and exercise | | | | 24 months |  |
| Einvik *et al.* (2010)^21^ | Counseling in order to increase the intake of healthy foods and to reduce weight. Meetings were once per 6 months. A random part of the participants also received n-3-PUFA’s | No active control. A random part of the participants also received n-3-PUFA | | | | 36 months |  |
| Imayama *et al.* (2011)^25^ ^a^ | Group sessions and email/phone contact with a dietician, mostly aimed at calorie restriction | No active control | | | | 12 months |  |
| Imayama *et al.* (2011)^25^ ^b^ | Group sessions + email/phone contact with a dietician, aimed at calorie restriction *plus* supervised exercise 5 days a week for 45 minutes per session | Supervised exercise 5 days a week for 45 minutes per session | | | | 12 months |  |
| McMillan *et al.* (2011)^23^ | Instructions aimed at adherence to a Mediterranean dietary pattern. Calorie intake was not restricted | No active control | | | | 10 days |  |
| Forster *et al.* (2012)^29^ | Dietary intervention focused on increased fruit and vegetable consumption | Pill-placebo | | | | 12 months |  |
| Agarwal *et al.* (2015)^42^ | Instructions to follow a vegan diet | No active control | | | | 18 weeks |  |
| Assaf *et al.* (2015)^49^ | 18 group sessions aimed at reducing dietary fat intake and increasing vegetable and fruit intake | No active control | | | | N.K*.* |  |
| **Prevention** |  |  | | | |  |  |
| Bot *et al.* (2019)^66^ ^a^ | A maximum of 21 individual- or group sessions of  food-related behavioral activation therapy in order to increase adherence to a Mediterranean dietary type | Placebo | | | | 12 months |  |
| Bot *et al.* (2019)^66^ ^b^ | A maximum of 21 individual- or group sessions of  food-related behavioral activation therapy in order to increase adherence to a Mediterranean dietary type *plus* dietary supplement | Dietary supplements | | | | 12 months |  |
| **Treatment** |  |  | | | |  |  |
| Kasckow et al. (2014 a)^38^ | 6-8 sessions of dietary education and coaching | 6-8 sessions of problem-solving therapy | | | | 12 weeks |  |
| Kasckow et al. (2014 b)^39^ | 6-8 sessions of dietary education and coaching | 6-8 sessions of problem-solving therapy | | | | 24 months |  |
| Jacka *et al.* (2017)^5^ | 7 sessions of individual counseling aimed at dietary improvement | 7 social support sessions | | | | 12 weeks |  |
| Parletta *et al.* (2019)^69^ | Fortnightly food hampers and cooking workshops in order to promote adherence to a Mediterranean diet | Fortnightly social group meetings | | | | 12 weeks |  |
| *Note.* N.K., Not known; n-3-PUFA, omega-3 polyunsaturated fatty acids. | | | | | | |  |
| **Table E.** Items that compose the tool to assess the methodological quality of treatment trials. | | | | | | | |
| **Item** | | | **Points** | | | | |
|  | | | Yes | No | Not known / N/A | | |
| **1** Was the study described as an RCT? | | | **⊕** = 1 | **⊗** = -1 | **∅** = 0 | | |
| **2** Was the method of randomization adequate? | | | **⊕** = 1 | **⊗** = -1 | **∅** = 0 | | |
| **3** Was the treatment allocation concealed? | | | **⊕** = 1 | **⊗** = -1 | **∅** = 0 | | |
| **4** Were study participants and providers blinded to treatment group assignment? | | | **⊕** = 1 | **⊗** = -1 | **∅** = 0 | | |
| **5** Were the people assessing the outcomes blinded to the group assignments? | | | **⊕** = 1 | **⊗** = -1 | **∅** = 0 | | |
| **6** Were the groups similar at baseline on characteristics that could affect outcomes? | | | **⊕** = 1 | **⊗** = -1 | **∅** = 0 | | |
| **7** Was the overall drop-out rate from the study at endpoint 20% or lower of the number allocated to treatment? | | | **⊕** = 1 | **⊗** = -1 | **∅** = 0 | | |
| **8** Was the differential drop-out rate at endpoint < 15%? | | | **⊕** = 1 | **⊗** = -1 | **∅** = 0 | | |
| **9** Was there high adherence to the intervention protocols for each treatment group? | | | **⊕** = 1 | **⊗** = -1 | **∅** = 0 | | |
| **10** Were other interventions avoided or similar in the groups? | | | **⊕** = 1 | **⊗** = -1 | **∅** = 0 | | |
| **11** Were outcomes assessed using valid and reliable measures, implemented consistently across all study participants? | | | **⊕** = 1 | **⊗** = -1 | **∅** = 0 | | |
| **12** Did the authors report that the sample size was sufficiently large to be able to detect a difference between groups with ≥ 80% power? | | | **⊕** = 1 | **⊗** = -1 | **∅** = 0 | | |
| **13** Were outcomes reported or subgroups analyzed pre-specified? | | | **⊕** = 1 | **⊗** = -1 | **∅** = 0 | | |
| **14** Were randomized participants analyzed in the group to which they were assigned? | | | **⊕** = 1 | **⊗** = -1 | **∅** = 0 | | |
| *Note*. N/A, not applicable. | | | | | | | |

| **Table F.** Methodological quality of included studies. | | | | | | | | | | | | | | | |
| --- | --- | --- | --- | --- | --- | --- | --- | --- | --- | --- | --- | --- | --- | --- | --- |
| **Author, year** | **I1** | **I2** | **I3** | **I4** | **I5** | **I6** | **I7** | **I8** | **I9** | **I10** | **I11** | **I12** | **I13** | **I14** | **Total** |
| **Association** |  |  |  |  |  |  |  |  |  |  |  |  |  |  |  |
| Nieman *et al.* (2000)^11^ ^a^ | **⊕** | **∅** | **⊗** | **⊗** | **⊗** | **∅** | **∅** | **∅** | **⊕** | **∅** | **⊗** | **⊗** | **⊗** | **∅** | -4 |
| Wardle *et al.* (2000)^3 a^ | **⊕** | **⊕** | **⊕** | **⊗** | **⊗** | **⊕** | **⊕** | **⊕** | **∅** | **∅** | **⊗** | **⊕** | **⊕** | **∅** | 5 |
| Kiernan *et al.* (2001)^12^ | **⊕** | **∅** | **∅** | **⊗** | **⊗** | **⊕** | **∅** | **∅** | **∅** | **∅** | **⊗** | **⊗** | **⊗** | **∅** | -3 |
| Hyyppä *et al.* (2003)^13^ | **⊕** | **∅** | **∅** | **⊗** | **⊗** | **⊕** | **⊕** | **⊕** | **∅** | **∅** | **⊗** | **⊕** | **∅** | **⊕** | 3 |
| Scheier *et al.* (2005)^15^ | **⊕** | **∅** | **∅** | **⊗** | **∅** | **⊕** | **⊕** | **⊕** | **⊕** | **∅** | **⊗** | **⊕** | **⊕** | **⊗** | 4 |
| Jenkinson *et al.* (2009)^20^ | **⊕** | **⊕** | **⊕** | **⊗** | **⊗** | **⊕** | **⊗** | **⊕** | **∅** | **∅** | **⊗** | **⊕** | **⊕** | **⊕** | 4 |
| Einvik *et al.* (2010)^21^ | **⊕** | **∅** | **∅** | **⊗** | **⊗** | **∅** | **∅** | **∅** | **∅** | **∅** | **⊗** | **∅** | **⊗** | **⊕** | -2 |
| Imayama *et al.* (2011)^25^ ^a^ | **⊕** | **∅** | **∅** | **⊗** | **⊗** | **⊕** | **⊕** | **⊕** | **⊕** | **∅** | **⊗** | **⊕** | **⊕** | **⊕** | 5 |
| McMillan *et al.* (2011)^23^ | **⊕** | **∅** | **∅** | **⊗** | **∅** | **⊕** | **⊕** | **⊕** | **⊕** | **∅** | **⊗** | **⊗** | **⊗** | **⊕** | 2 |
| Forster *et al.* (2012)^29^ | **⊕** | **⊕** | **⊗** | **⊗** | **∅** | **⊕** | **∅** | **∅** | **⊕** | **∅** | **⊗** | **⊕** | **⊕** | **∅** | 3 |
| Agarwal *et al.* (2015)^42^ | **⊕** | **∅** | **∅** | **⊗** | **∅** | **⊗** | **⊗** | **⊕** | **⊕** | **∅** | **⊗** | **⊗** | **⊕** | **⊕** | 0 |
| Assaf *et al.* (2015)^49^ | **⊕** | **∅** | **∅** | **⊗** | **⊗** | **⊕** | **⊕** | **⊕** | **⊕** | **∅** | **⊗** | **⊕** | **⊕** | **⊕** | 5 |
| **Prevention** |  |  |  |  |  |  |  |  |  |  |  |  |  |  |  |
| Bot *et al.* (2019)^66^ ^a^ | **⊕** | **⊕** | **⊕** | **⊗** | **⊗** | **⊕** | **⊕** | **⊕** | **⊕** | **∅** | **⊗** | **⊗** | **⊕** | **⊕** | 5 |
| **Treatment** |  |  |  |  |  |  |  |  |  |  |  |  |  |  |  |
| Kasckow et al. (2014 a)^38^ | **⊕** | **∅** | **∅** | **⊗** | **∅** | **⊕** | **⊗** | **∅** | **⊕** | **∅** | **⊗** | **⊗** | **⊗** | **∅** | -2 |
| Kasckow et al. (2014 b)^39^ | **⊕** | **∅** | **∅** | **⊗** | **∅** | **⊕** | **⊕** | **⊕** | **⊕** | **∅** | **⊗** | **⊗** | **⊕** | **∅** | 3 |
| Jacka *et al.* (2017)^5^ | **⊕** | **⊗** | **∅** | **⊗** | **⊗** | **∅** | **⊗** | **⊗** | **∅** | **∅** | **⊗** | **⊗** | **⊕** | **⊕** | -4 |
| Parletta *et al.* (2019)^69^ | **⊕** | **⊕** | **∅** | **⊗** | **⊗** | **∅** | **⊗** | **⊗** | **∅** | **∅** | **⊗** | **⊗** | **⊕** | **⊕** | -2 |
| *Note*. I, Item number. ⊕ = + 1 point; ⊗ = – 1 point; ∅ = 0 point. | | | | | | | | | | | | | | | |

**
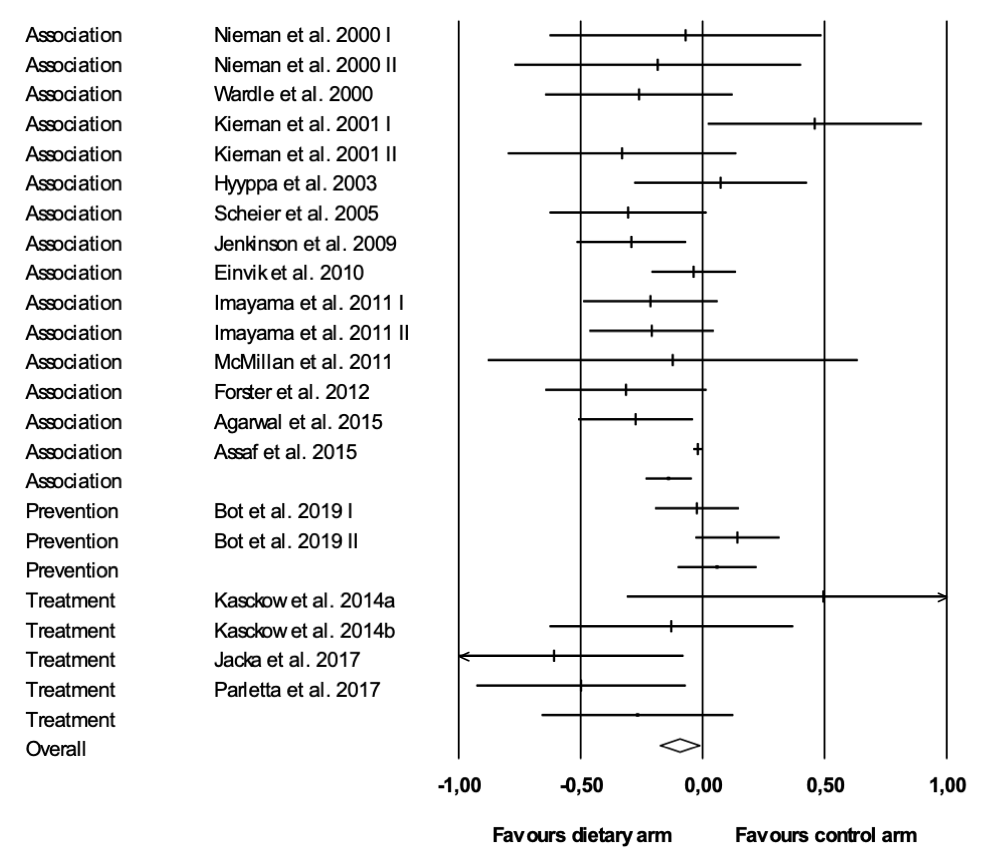
**

**Fig B. Forrest plot on the effect of diet on depression for association, prevention, and treatment studies.**

**References**

1 Firth J, Marx W, Dash S, Carney R, Teasdale SB, Solmi M *et al.* The Effects of Dietary Improvement on Symptoms of Depression and Anxiety: A Meta-Analysis of Randomized Controlled Trials. *Psychosom Med* 2019; **81**: 265–280.

2 Endevelt R, Lemberger J, Bregman J, Kowen G, Berger-Fecht I, Lander H *et al.* Intensive dietary intervention by a dietitian as a case manager among community dwelling older adults: The edit study. *J Nutr Heal Aging* 2011; **15**: 624–630.

3 Wardle J, Rogers P, Judd P, Taylor MA, Rapoport L, Green M *et al.* Randomized trial of the effects of cholesterol-lowering dietary treatment on psychological function. *Am J Med* 2000; **108**: 547–553.

4 Khan A, Brown WA. Antidepressants versus placebo in major depression: an overview. *World Psychiatry* 2015; **14**: 294–300.

5 Jacka FN, O’Neil A, Opie R, Itsiopoulos C, Cotton S, Mohebbi M *et al.* A randomised controlled trial of dietary improvement for adults with major depression (the ‘SMILES’ trial). *BMC Med* 2017; **15**: 23.

6 Molendijk ML, Fried EI, Van der Does W. The SMILES trial: do undisclosed recruitment practices explain the remarkably large effect? *BMC Med* 2018; **16**: 243.

7 Rosen JC, Hunt DA, Sims EA, Bogardus C. Comparison of carbohydrate-containing and carbohydrate-restricted hypocaloric diets in the treatment of obesity: effects on appetite and mood. *Am J Clin Nutr* 1982; **36**: 463–469.

8 Lloyd HM, Rogers PJ, Hedderley DI, Walker AF. Acute Effects on Mood and Cognitive Performance of Breakfasts Differing in Fat and Carbohydrate Content. *Appetite* 1996; **27**: 151–164.

9 Benton D, Haller J, Fordy J. Vitamin supplementation for 1 year improves mood. *Neuropsychobiology* 1995; **32**: 98–105.

10 Wells AS, Read NW, Laugharne JDE, Ahluwalia NS. Alterations in mood after changing to a low-fat diet. *Br J Nutr* 1998; **79**: 23–30.

11 Nieman DC, Custer WF, Butterworth DE, Utter AC, Henson DA. Psychological response to exercise training and/or energy restriction in obese women. *J Psychosom Res* 2000; **48**: 23–29.

12 Kiernan M, King AC, Stefanick ML, Killen JD. Men gain additional psychological benefits by adding exercise to a weight-loss program. *Obes Res* 2001; **9**: 770–777.

13 Hyyppä MT, Kronholm E, Virtanen A, Leino A, Jula A. Does simvastatin affect mood and steroid hormone levels in hypercholesterolemic men? A randomized double-blind trial. *Psychoneuroendocrinology* 2003; **28**: 181–194.

14 Ness AR, Gallacher JEJ, Bennett PD, Gunnell DJ, Rogers PJ, Kessler D *et al.* Advice to eat fish and mood: A randomised controlled trial in men with angina. *Nutr Neurosci* 2003; **6**: 63–65.

15 Scheier MF, Helgeson VS, Schulz R, Calvin S, Berga S, Bridges MW *et al.* Interventions to enhance physical and psychological functioning among younger women who are ending nonhormonal adjuvant treatment for early-stage breast cancer. *J Clin Oncol* 2005; **23**: 4298–4311.

16 Halyburton AK, Brinkworth GD, Wilson CJ, Noakes M, Buckley JD, Keogh JB *et al.* Low- and high-carbohydrate weight-loss diets have similar effects on mood but not cognitive performance. *Am J Clin Nutr* 2007; **86**: 580–587.

17 Merrill RM, Taylor P, Aldana SG. Coronary Health Improvement Project (CHIP) is associated with improved nutrient intake and decreased depression. *Nutrition* 2008; **24**: 314–321.

18 Brinkworth GD, Buckley JD, Noakes M, Clifton PM, Wilson CJ. Long-term effects of a very low-carbohydrate diet and a low-fat diet on mood and cognitive function. *Arch Intern Med* 2009; **169**: 1873–1880.

19 Cheatham RA, Roberts SB, Das SK, Gilhooly CH, Golden JK, Hyatt R *et al.* Long-term effects of provided low and high glycemic load low energy diets on mood and cognition. *Physiol Behav* 2009; **98**: 374–379.

20 Jenkinson CM, Doherty M, Avery AJ, Read A, Taylor MA, Sach TH *et al.* Effects of dietary intervention and quadriceps strengthening exercises on pain and function in overweight people with knee pain: Randomised controlled trial. *BMJ* 2009; **339**: 606–609.

21 Einvik G, Ekeberg O, Lavik JG, Ellingsen I, Klemsdal TO, Hjerkinn EM. The influence of long-term awareness of hyperlipidemia and of 3 years of dietary counseling on depression, anxiety, and quality of life. *J Psychosom Res* 2010; **68**: 567–572.

22 Thomson RL, Buckley JD, Lim SS, Noakes M, Clifton PM, Norman RJ *et al.* Lifestyle management improves quality of life and depression in overweight and obese women with polycystic ovary syndrome. *Fertil Steril* 2010; **94**: 1812–1816.

23 McMillan L, Owen L, Kras M, Scholey A. Behavioural effects of a 10-day Mediterranean diet. Results from a pilot study evaluating mood and cognitive performance. *Appetite* 2011; **56**: 143–147.

24 Ciarambino T, Ferrara N, Castellino P, Paolisso G, Coppola L, Giordano M. Effects of a 6-days-a-week low protein diet regimen on depressive symptoms in young-old type 2 diabetic patients. *Nutrition* 2011; **27**: 46–49.

25 Imayama I, Alfano CM, Kong A, Foster-Schubert KE, Bain CE, Xiao L *et al.* Dietary weight loss and exercise interventions effects on quality of life in overweight/obese postmenopausal women: a randomized controlled trial. *Int J Behav Nutr Phys Act* 2011; **8**: 118.

26 Beezhold BL, Johnston CS. Restriction of meat, fish, and poultry in omnivores improves mood: A pilot randomized controlled trial. *Nutr J* 2012; **11**: 1–5.

27 Casañas R, Catalán R, del Val JL, Real J, Valero S, Casas M. Effectiveness of a psycho-educational group program for major depression in primary care: a randomized controlled trial. *BMC Psychiatry* 2012; **12**: 230.

28 Ciarambino T, Castellino P, Paolisso G, Coppola L, Ferrara N, Signoriello G *et al.* Long term effects of low protein diet on depressive symptoms and quality of life in elderly Type 2 diabetic patients. *Clin Nephrol* 2012; **78**: 122—128.

29 Forster SE, Powers HJ, Foulds GA, Flower DJ, Hopkinson K, Parker SG *et al.* Improvement in nutritional status reduces the clinical impact of infections in older adults. *J Am Geriatr Soc* 2012; **60**: 1645–1654.

30 García-Toro M, Ibarra O, Gili M, Serrano MJ, Oliván B, Vicens E *et al.* Four hygienic-dietary recommendations as add-on treatment in depression: A randomized-controlled trial. *J Affect Disord* 2012; **140**: 200–203.

31 Garcia-Toro M, Roca M, Monzón S, Vives M, Oliván B, Vicens E *et al.* Hygienic-dietary recommendations for major depression treatment: Study protocol of a randomized controlled trial. *BMC Psychiatry* 2012; **12**: 2–7.

32 Torres SJ, Nowson CA. A moderate-sodium DASH-type diet improves mood in postmenopausal women. *Nutrition* 2012; **28**: 896–900.

33 Ruusunen A, Voutilainen S, Karhunen L, Lehto SM, Tolmunen T, Keinänen-Kiukaanniemi S *et al.* How does lifestyle intervention affect depressive symptoms? Results from the Finnish Diabetes Prevention Study. *Diabet Med* 2012; **29**: 126–132.

34 Petasne Nijamkin M, Campa A, Samiri Nijamkin S, Sosa J. Comprehensive behavioral-motivational nutrition education improves depressive symptoms following bariatric surgery: A randomized, controlled trial of obese hispanic Americans. *J Nutr Educ Behav* 2013; **45**: 620–626.

35 Sánchez-Villegas A, Martínez-González MA, Estruch R, Salas-Salvadó J, Corella D, Covas MI *et al.* Mediterranean dietary pattern and depression: the PREDIMED randomized trial. *BMC Med* 2013; **11**: 208.

36 Shahnazari M, Ceresa C, Foley S, Fong A, Zidaru E, Moody S. Nutrition-Focused Wellness Coaching Promotes a Reduction in Body Weight in Overweight US Veterans. *J Acad Nutr Diet* 2013; **113**: 928–935.

37 Stange I, Bartram M, Liao Y, Poeschl K, Kolpatzik S, Uter W *et al.* Effects of a Low-Volume, Nutrient- and Energy-Dense Oral Nutritional Supplement on Nutritional and Functional Status: A Randomized, Controlled Trial in Nursing Home Residents. *J Am Med Dir Assoc* 2013; **14**: 628.e1-628.e8.

38 Kasckow J, Morse J, Begley A, Anderson S, Bensasi S, Thomas S *et al.* Treatment of post traumatic stress disorder symptoms in emotionally distressed individuals. *Psychiatry Res* 2014; **220**: 370–375.

39 Kasckow J, Klaus J, Morse J, Oslin D, Luther J, Fox L *et al.* Using problem solving therapy to treat veterans with subsyndromal depression: a pilot study. *Int J Geriatr Psychiatry* 2014; **29**: 1255–1261.

40 Reynolds CF, Thomas SB, Morse JQ, Anderson SJ, Albert S, Dew MA *et al.* Early intervention to preempt major depression among older black and white adults. *Psychiatr Serv* 2014; **65**: 765–773.

41 Stahl ST, Albert SM, Dew MA, Lockovich MH, Reynolds CF. Coaching in healthy dietary practices in at-risk older adults: A case of indicated depression prevention. *Am J Psychiatry* 2014; **171**: 499–505.

42 Agarwal U, Mishra S, Xu J, Levin S, Gonzales J, Barnard ND. A multicenter randomized controlled trial of a nutrition intervention program in a multiethnic adult population in the corporate setting reduces depression and anxiety and improves quality of life: The GEICO study. *Am J Heal Promot* 2015; **29**: 245–254.

43 Bartels SJ, Pratt SI, Aschbrenner KA, Barre LK, Naslund JA, Wolfe R *et al.* Pragmatic replication trial of health promotion coaching for obesity in serious mental illness and maintenance of outcomes. *Am J Psychiatry* 2015; **172**: 344–352.

44 Forsyth A, Deane FP, Williams P. A lifestyle intervention for primary care patients with depression and anxiety: A randomised controlled trial. *Psychiatry Res* 2015; **230**: 537–544.

45 Knight A, Bryan J, Wilson C, Hodgson J, Murphy K. A randomised controlled intervention trial evaluating the efficacy of a Mediterranean dietary pattern on cognitive function and psychological wellbeing in healthy older adults: The MedLey study. *BMC Geriatr* 2015; **15**: 1–14.

46 Melnyk BM, Jacobson D, Kelly SA, Belyea MJ, Shaibi GQ, Small L *et al.* Twelve-Month Effects of the COPE Healthy Lifestyles TEEN Program on Overweight and Depressive Symptoms in High School Adolescents. *J Sch Health* 2015; **85**: 861–870.

47 Suominen MH, Puranen TM, Jyväkorpi SK, Eloniemi-Sulkava U, Kautiainen H, Siljamäki-Ojansuu U *et al.* Nutritional guidance improves nutrient intake and quality of life, and may prevent falls in aged persons with Alzheimer disease living with a spouse (NuAD trial). *J Nutr Heal Aging* 2015; **19**: 901–907.

48 Watson NA, Dyer KA, Buckley JD, Brinkworth GD, Coates AM, Parfitt G *et al.* A randomised trial comparing low-fat diets differing in carbohydrate and protein ratio, combined with regular moderate intensity exercise, on glycaemic control, cardiometabolic risk factors, food cravings, cognitive function and psychological wellbeing in. *Contemp Clin Trials* 2015; **45**: 217–225.

49 Assaf AR, Beresford SAA, Risica PM, Aragaki A, Brunner RL, Bowen DJ *et al.* Low-Fat Dietary Pattern Intervention and Health-Related Quality of Life: The Women’s Health Initiative Randomized Controlled Dietary Modification Trial. *J Acad Nutr Diet* 2016; **116**: 259–271.

50 Brinkworth GD, Luscombe-Marsh ND, Thompson CH, Noakes M, Buckley JD, Wittert G *et al.* Long-term effects of very low-carbohydrate and high-carbohydrate weight-loss diets on psychological health in obese adults with type 2 diabetes: randomized controlled trial. *J Intern Med* 2016; **280**: 388–397.

51 Dodd JM, Newman A, Moran LJ, Deussen AR, Grivell RM, Yelland LN *et al.* The effect of antenatal dietary and lifestyle advice for women who are overweight or obese on emotional well-being: The LIMIT randomized trial. *Acta Obstet Gynecol Scand* 2016; **95**: 309–318.

52 Moncrieft AE, Llabre MM, McCalla JR, Gutt M, Mendez AJ, Gellman MD *et al.* Effects of a Multicomponent Life-Style Intervention on Weight, Glycemic Control, Depressive Symptoms, and Renal Function in Low-Income, Minority Patients With Type 2 Diabetes: Results of the Community Approach to Lifestyle Modification for Diabetes Random. *Psychosom Med* 2016; **78**: 851–860.

53 Roca M, Kohls E, Gili M, Watkins E, Owens M, Hegerl U *et al.* Prevention of depression through nutritional strategies in high-risk persons: rationale and design of the MooDFOOD prevention trial. *BMC Psychiatry* 2016; **16**: 192.

54 Shomaker LB, Kelly NR, Pickworth CK, Cassidy OL, Radin RM, Shank LM *et al.* A Randomized Controlled Trial to Prevent Depression and Ameliorate Insulin Resistance in Adolescent Girls at Risk for Type 2 Diabetes. *Ann Behav Med* 2016; **50**: 762–774.

55 Webber KH, Casey EM, Mayes L, Katsumata Y, Mellin L. A comparison of a behavioral weight loss program to a stress management program: A pilot randomized controlled trial. *Nutrition* 2016; **32**: 904–909.

56 Cicero AF, Bove M, Colletti A, Rizzo M, Fogacci F, Giovannini M *et al.* Short-Term Impact of a Combined Nutraceutical on Cognitive Function, Perceived Stress and Depression in Young Elderly with Cognitive Impairment: A Pilot, Double-Blind, Randomized Clinical Trial. *J Prev Alzheimer’s Dis* 2017; **4**: 12–15.

57 Ng TP, Nyunt MSZ, Feng L, Niti M, Tan BY, Chan SM *et al.* Multi-Domains Lifestyle Interventions Reduces Depressive Symptoms Among Frail and Pre-Frail. *J Nutr Heal Aging* 2017; **21**: 918–926.

58 Sharifan P, Hosseini MS, Sharifan A. The interventional relationship between frequent fish consumption and depression symptoms in aging adults: a randomized controlled trial. *Int J Geriatr Psychiatry* 2017; **32**: e116–e122.

59 Stewart RC, Ashorn P, Umar E, Dewey KG, Ashorn U, Creed F *et al.* The impact of maternal diet fortification with lipid-based nutrient supplements on postpartum depression in rural Malawi: a randomised-controlled trial. *Matern Child Nutr* 2017; **13**: 1–14.

60 Swoboda CM, Miller CK, Wills CE. Impact of a goal setting and decision support telephone coaching intervention on diet, psychosocial, and decision outcomes among people with type 2 diabetes. *Patient Educ Couns* 2017; **100**: 1367–1373.

61 Young AJ, Marriott BP, Champagne CM, Hawes MR, Montain SJ, Johannsen NM *et al.* Blood fatty acid changes in healthy young Americans in response to a 10-week diet that increased n-3 and reduced n-6 fatty acid consumption: A randomised controlled trial. *Br J Nutr* 2017; **117**: 1257–1269.

62 Jamilian M, Samimi M, Mirhosseini N, Afshar Ebrahimi F, Aghadavod E, Talaee R *et al.* The influences of vitamin D and omega-3 co-supplementation on clinical, metabolic and genetic parameters in women with polycystic ovary syndrome. *J Affect Disord* 2018; **238**: 32–38.

63 Sánchez-Villegas A, Álvarez-Pérez J, Toledo E, Salas-Salvadó J, Ortega-Azorín C, Zomeño MD *et al.* Seafood consumption, omega-3 fatty acids intake, and life-time prevalence of depression in the PREDIMED-plus trial. *Nutrients* 2018; **10**. doi:10.3390/nu10122000.

64 Sarris J, Byrne GJ, Bousman C, Stough C, Murphy J, MacDonald P *et al.* Adjunctive S-adenosylmethionine (SAMe) in treating non-remittent major depressive disorder: An 8-week double-blind, randomized, controlled trial. *Eur Neuropsychopharmacol* 2018; **28**: 1126–1136.

65 Berk M, Turner A, Malhi GS, Ng CH, Cotton SM, Dodd S *et al.* Correction to: A randomised controlled trial of a mitochondrial therapeutic target for bipolar depression: Mitochondrial agents, N-acetylcysteine, and placebo (BMC Medicine (2019) 17 (18) DOI: 10.1186/s12916-019-1257-1). *BMC Med* 2019; **17**: 1–11.

66 Bot M, Brouwer IA, Roca M, Kohls E, Penninx BWJHJH, Watkins E *et al.* Effect of Multinutrient Supplementation and Food-Related Behavioral Activation Therapy on Prevention of Major Depressive Disorder Among Overweight or Obese Adults With Subsyndromal Depressive Symptoms. *JAMA* 2019; **321**: 858.

67 Ma J, Rosas LG, Lv N, Xiao L, Snowden MB, Venditti EM *et al.* Effect of Integrated Behavioral Weight Loss Treatment and Problem-Solving Therapy on Body Mass Index and Depressive Symptoms Among Patients With Obesity and Depression. *JAMA* 2019; **321**: 869.

68 Nishi D, Su KP, Usuda K, Pei-Chen Chang J, Chiang YJ, Chen HT *et al.* The efficacy of omega-3 fatty acids for depressive symptoms among pregnant women in Japan and Taiwan: A randomized, double-blind, placebo-controlled trial (SYNCHRO; NCT01948596). *Psychother Psychosom* 2019; **88**: 122–124.

69 Parletta N, Zarnowiecki D, Cho J, Wilson A, Bogomolova S, Villani A *et al.* A Mediterranean-style dietary intervention supplemented with fish oil improves diet quality and mental health in people with depression: A randomized controlled trial (HELFIMED). *Nutr Neurosci* 2019; **22**: 474–487.
