# Supplementary material for: Impact of review method on the conclusions of clinical reviews: A systematic review on dietary interventions in depression as a case in point
Source: PLoS One. 2020 Sep 16;15(9):e0238131. doi: 10.1371/journal.pone.0238131 (PMC7494108; doi:10.1371/journal.pone.0238131)
Supplement: S1 Table — (DOCX) [file pone.0238131.s004.docx]

| **Table G.** Excluded studies with reasons (in chronological and alphabetical order) | | |
| --- | --- | --- |
| **Author, year** | **Study type** | **Reason** |
| Hodkinson (1988)^1^ | Narrative review | No depression outcome |
| Christensen (1993)^2^ | Narrative review | No dietary exposure |
| Prasad (1998)^3^ | Narrative review | No dietary exposure |
| Ottley (2000)^4^ | Narrative review | No dietary exposure |
| Rogers (2001)^5^ | Narrative review | No dietary exposure |
| Hausteiner *et al.* (2007)^6^ | Narrative review | No dietary exposure |
| Melanson (2007)^7^ | Narrative review | No dietary exposure |
| Gomez-Pinilla (2008)^8^ | Narrative review | No depression outcome |
| Watts (2011)^9^ | Narrative review | No dietary exposure |
| Lopresti *et al.* (2013)^10^ | Narrative review | No dietary exposure |
| Nanri (2013)^11^ | Narrative review | No review |
| Cairns *et al.* (2014)^12^ | Meta-analysis | No dietary exposure |
| Hibbeln & Gow (2014)^13^ | Narrative review | No dietary exposure |
| Logan & Jacka (2014)^14^ | Narrative review | No dietary exposure |
| Tsai *et al.* (2014)^15^ | Cohort study | No review |
| Dennison *et al.* (2016)^16^ | Narrative review | No dietary exposure |
| Martínez-González & Sánchez-Villegas (2016)^17^ | Conference paper | No review |
| Bostock et al. (2017)^18^ | Narrative review | Animal models |
| Gould *et al.* (2017)^19^ | Narrative review | No dietary exposure |
| Marx *et al.* (2017)^20^ | Conference paper | No review |
| Owen & Corfe (2017)^21^ | Editorial | No review |
| Sparling *et al.* (2017)^22^ | Systematic review | No depression outcome |
| LaChance & Ramsey (2018)^23^ | Systematic review | No dietary exposure |
| Lopez *et al.* (2018)^24^ | Systematic review | No dietary exposure |
| Firth *et al.* (2019)^25^ | Meta-analysis | Invalid results |
| Jesus *et al.* (2019)^26^ | Narrative review | Inaccessible |
| Khanna *et al.* (2019)^27^ | Systematic review | Suspected plagiarism |
| Melo *et al.* (2019)^28^ | Narrative review | No dietary exposure |
| Sarris *et al.* (2019)^29^ | Narrative review | No dietary exposure |
| Silva *et al.* (2019)^30^ | Systematic review | No dietary exposure |
| Opie *et al.* (2020)^31^ | Systematic review | No depression outcome |

References

1 Hodkinson HM. Diet and Maintenance of Mental Health in the Elderly. *Nutr Rev* 1988; **46**: 79–82.

2 Christensen L. Effects of eating behavior on mood: A review of the literature. *Int J Eat Disord* 1993; **14**: 171–183.

3 Prasad C. Food, mood and health: A neurobiologic outlook. *Brazilian J Med Biol Res* 1998; **31**: 1517–1527.

4 Ottley C. Food and mood. *Ment Heal Pract* 2000; **4**.

5 Rogers PJ. A healthy body, a healthy mind: long-term impact of diet on mood and cognitive function. *Proc Nutr Soc* 2001; **60**: 135–143.

6 Hausteiner C, Bornschein S, Zilker T, Förstl H, Graßmann J. Über Den Möglichen Einfluss Der Ernährung Auf Die Psychische Gesundheit. *Nervenarzt* 2007; **78**: 696–705.

7 Melanson KJ. Nutrition Review: Relationships of Nutrition With Depression and Anxiety. *Am J Lifestyle Med* 2007; **1**: 171–174.

8 Gomez-Pinilla F. The influences of diet and exercise on mental health through hormesis. *Ageing Res Rev* 2008; **7**: 49–62.

9 Watts MK. Nutritional therapy in practice for learning, behavioural and mood disorders. *Nutr Health* 2011; **20**: 239–254.

10 Lopresti AL, Hood SD, Drummond PD. A review of lifestyle factors that contribute to important pathways associated with major depression: Diet, sleep and exercise. *J Affect Disord* 2013; **148**: 12–27.

11 Nanri A. Nutritional epidemiology of type 2 diabetes and depressive symptoms. *J Epidemiol* 2013; **23**: 243–250.

12 Cairns KE, Yap MBH, Pilkington PD, Jorm AF. Risk and protective factors for depression that adolescents can modify: A systematic review and meta-analysis of longitudinal studies. *J Affect Disord* 2014; **169**: 61–75.

13 Hibbeln JR, Gow R V. The Potential for Military Diets to Reduce Depression, Suicide, and Impulsive Aggression: A Review of Current Evidence for Omega-3 and Omega-6 Fatty Acids. *Mil Med* 2014; **179**: 117–128.

14 Logan AC, Jacka FN. Nutritional psychiatry research: An emerging discipline and its intersection with global urbanization, environmental challenges and the evolutionary mismatch. *J Physiol Anthropol* 2014; **33**: 1–16.

15 Tsai AC, Lucas M, Okereke OI, O’Reilly ÉJ, Mirzaei F, Kawachi I *et al.* Suicide Mortality in Relation to Dietary Intake of n-3 and n-6 Polyunsaturated Fatty Acids and Fish: Equivocal Findings From 3 Large US Cohort Studies. *Am J Epidemiol* 2014; **179**: 1458–1466.

16 Dennison M, Sisson SB, Morris A. Obesogenic behaviours and depressive symptoms in children: a narrative literature review. *Obes Rev* 2016; **17**: 735–757.

17 Martínez-González MA, Sánchez-Villegas A. Food patterns and the prevention of depression. *Proc Nutr Soc* 2016; **75**: 139–146.

18 Bostock ECS, Kirkby KC, Taylor BVM. The Current Status of the Ketogenic Diet in Psychiatry. *Front Psychiatry* 2017; **8**: 1–10.

19 Gould JF, Best K, Makrides M. Perinatal nutrition interventions and post-partum depressive symptoms. *J Affect Disord* 2017; **224**: 2–9.

20 Marx W, Moseley G, Berk M, Jacka F. Nutritional psychiatry: The present state of the evidence. *Proc Nutr Soc* 2017; **76**: 427–436.

21 Owen L, Corfe B. The role of diet and nutrition on mental health and wellbeing. *Proc Nutr Soc* 2017; **76**: 425–426.

22 Sparling TM, Henschke N, Nesbitt RC, Gabrysch S. The role of diet and nutritional supplementation in perinatal depression: a systematic review. *Matern Child Nutr* 2017; **13**: mcn.12235.

23 LaChance LR, Ramsey D. Antidepressant foods: An evidence-based nutrient profiling system for depression. *World J Psychiatry* 2018; **8**: 97–104.

24 Quintana VAL, Díaz KJL, Caire GJ. Interventions to improve healthy lifestyles and their effects on psychological variables among breast cancer survivors: a systematic review. *Nutr Hosp* 2018; **35**: 979–992.

25 Firth J, Marx W, Dash S, Carney R, Teasdale SB, Solmi M *et al.* The Effects of Dietary Improvement on Symptoms of Depression and Anxiety: A Meta-Analysis of Randomized Controlled Trials. *Psychosom Med* 2019; **81**: 265–280.

26 Jesus M, Silva T, Cagigal C, Martins V, Silva C. Dietary Patterns: A New Therapeutic Approach for Depression? *Curr Pharm Biotechnol* 2019; **20**: 123–129.

27 Khanna P, Chattu V, Aeri B. Nutritional aspects of depression in adolescents - A systematic review. *Int J Prev Med* 2019; **10**: 42.

28 Melo HM, Santos LE, Ferreira ST. Diet-Derived Fatty Acids, Brain Inflammation, and Mental Health. *Front Neurosci* 2019; **13**: 265.

29 Sarris J. Nutritional Psychiatry: From Concept to the Clinic. *Drugs* 2019; **79**: 929–934.

30 Silva DFO, Cobucci RN, Gonçalves AK, Lima SCVC. Systematic review of the association between dietary patterns and perinatal anxiety and depression. *BMC Pregnancy Childbirth* 2019; **19**: 212.

31 Opie RS, Uldrich AC, Ball K. Maternal Postpartum Diet and Postpartum Depression: A Systematic Review. *Matern Child Health J* 2020. doi:10.1007/s10995-020-02949-9.
