# Supplementary material for: Impact of review method on the conclusions of clinical reviews: A systematic review on dietary interventions in depression as a case in point
Source: PLoS One. 2020 Sep 16;15(9):e0238131. doi: 10.1371/journal.pone.0238131 (PMC7494108; doi:10.1371/journal.pone.0238131)
Supplement: S2 Table — (DOCX) [file pone.0238131.s005.docx]

| **Table H.** Basic characteristics of included meta-analyses and systematic reviews (in chronological and alphabetical order) | | | | |
| --- | --- | --- | --- | --- |
| **Author, year** | **Intervention** | **Comparators** | **Dietary assessment** | **Outcome** |
| *Meta-analyses* |  |  |  |  |
| Psaltopoulou *et al.* (2013)^1^ | *Dietary pattern:* Mediterranean diet | Highest vs. lowest, moderate vs. lowest category | FFQ | *Depressive symptoms***:** GDS, CES-D, ZDRS, HADS; *formal diagnosis* (physician-made); *antidepressant medication* |
| Lai *et al.* (2014)^2^ | *Dietary pattern***:** Healthy, Western diet | Highest vs. lowest category | FFQ, 24-h recall | **﻿***Depressive symptoms:* CES-D, GDS, HADS, HANDLS, ICD, DSM-IV; *formal diagnosis:* WHO CIDI, SCID, self-reported clinical diagnosis |
| Rahe *et al.* (2014)^3^ | *Dietary pattern*: Healthy / traditional diet, Unhealthy / Western diet | Highest vs. lowest category | FFQ, DHQ, 24-h recall | *Depressive symptom*: CES-D, EPDS, K10, HADS; *self-reported diagnosis;* *antidepressant medication* |
| Li *et al.* (2015)^4^ | *Food group:* Fish | Highest vs. lowest category | FFQ, ﻿DHQ, diet history interview, dietary records | *Depressive symptoms:* CES-D, BDI, HSCL, GDS; *formal diagnosis*: DSM-5, ICD-10, ﻿M-CIDI, *physician diagnosis;* *antidepressant medication* |
| Grosso *et al.* (2016)^5^ | *Food group:* Fish | Dose-response, highest vs. lowest category, some continuous | FFQ; 24-h dietary recall | *Depressive symptoms*: ﻿BDI-Y; CES-D, CIDI-SF, DASS-21, GDS, EPDS, M-CIDI; *mix*: diagnosis, hospitalization, medicament prescription |
| Liu *et al.* (2016)^6^ | *Food groups:* Fruits, vegetables | Highest vs. lowest category | FFQ, Interview, 24-h recall, dietary history method | *Depressive symptoms*: BDI, GDS, CES-D, CIDI-SF, Burnam 8-item scale; *formal diagnosis* (self-reported); *antidepressant medication* |
| Li *et al.* (2017)^7^ | *Dietary pattern:* Healthy, Western-style diet | Highest vs. lowest category | FFQ | *Depressive symptoms; self-reported diagnosis; antidepressant medication* |
| Molendijk *et al.* (2018)^8^ | *Dietary pattern:* Healthy; unhealthy diet; *food groups***:** Healthy (fish, fruit, legumes, nuts, pulses, seafood, seeds, soy, vegetables), unhealthy (junk/fast food; meat; refined grain), neutral (cereals, dairy, eggs, potatoes, starch, rice). | Dose-response, relative use (quantiles); depression risk as a function of first, second, third or fourth exposure category | FFQ, YAQ, dietary recall, single question, other questionnaires | *Depressive symptoms:* CES–D, EPDS, GDS, K10, SDQ; *mix:* formal diagnosis/depression symptom scales and/or the prescription of antidepressant drugs; *antidepressant medication* |
| Saghafian *et al.* (2018)^9^ | *Food groups:* Fruits, vegetables | Highest vs. lowest category | FFQ, 24-h recall, DHQ, food questionnaire, Interview | *Depressive symptoms:* CES-D, DASS, GDS, BDI, ﻿CIDI-SF, M-CIDI, MHI-5, Burnham 8-item scale; *formal diagnosis:* ICD-9/10 |
| Yang *et al.* (2018)^10^ | *Food groups:* Fish | Highest vs. lowest category | FFQ, 24-h recall | *Depressive symptoms:* CES-D, Burnam 8-item scale; *formal diagnosis* |
| Lassale *et al.* (2019)^11^ | *Dietary pattern:* Mediterranean /  healthy / prudent diet (AHEI, HEI, DASH, DII) | Highest vs. lowest category | FFQ, DQES, DHQ, 3-day food record, 4-day diet dairy, 24-h recall | *Depressive symptoms:* BDI, CES-D, DASS, GDS, GHQ-12, HADS(-D), K10, MFQ, PHQ-9; *formal diagnosis* (self-reported, study staff, medical records); *antidepressant medication* |

| ***Table H*** *continues on next page* |
| --- |

| ***Table H*** *continued* |
| --- |

| **Author, year** | **Intervention** | **Comparators** | **Dietary assessment** | **Outcome** |
| --- | --- | --- | --- | --- |
| Nicolaou *et al.* (2019)^12^ | *Dietary pattern***:** Mediterranean diet, healthy diet (MDS, AHEI, DASH) | Continuous, highest vs. lowest category | FFQ | *Depressive symptoms:* CES-D, IDS, PHQ-9 |
| Salari-Moghaddam *et al.* (2019)^13^ | *Dietary pattern*: Diet with high vs. low glycemic index, glycemic load | Highest vs. lowest category (cross-sectional, cohort); high-GL vs. low-GL diet, or low-GI diet vs. American Diabetes Association diet (RCTs) | ﻿FFQ, DHQ, 7-day weighing records | *Depressive symptoms*: CES-D, GDS, HADS, Burnam eight-item scale, POMS |
| Shafiei *et al.* (2019)^14^ | *Dietary pattern*: Mediterranean diet | Highest vs. lowest category | FFQ, 24-h recall, DQES | *Depressive symptoms*: ﻿CES-D, GDS, HADS, ZDRS, K10 |
| *Systematic reviews* |  |  |  |  |
| Murakami *et al.* (2010)^15^ | *Food group*: Fish (but also ﻿vitamins, minerals, and isoflavones, with focus on folate and other B vitamins) | Highest vs. lowest category | FFQ, DHQ, ﻿BDHQ, ﻿DHI, YAQ | *Depressive symptoms*: CES-D, EPDS, BDI, HADS, ZDRS, SF-36, HPL, HSCL, PHQ, GDS, DASS, WPDS, self-reported symptoms |
| Quirk *et al.* (2013)^16^ | ﻿*Dietary pattern*: Mediterranean or Norwegian diets, healthy, unhealthy, western diet | Dose-response, relative use (quantiles): depression risk as a function of first, second, third or fourth exposure category | FFQ, BDHQ, USDA AMPM, self-reported questionnaires, single dietary habits question, other dietary recall methods | *Depressive symptoms*: CES-D, GDS, EPDS, PHQ, CIDI, SCID-I/NP, HADS-D, M-BDI, DQI-P |
| Sanhueza *et al.* (2013)^17^ | *Dietary pattern*: whole versus processed diet, degree of adherence to the Mediterranean diet; *food groups*: (fish, olive oil and seed oils). | Highest vs. lowest category | FFQ, 24-h recall, DHQ, 4-day food recording | *Depressive symptoms*: CES-D, GDS, SDM; Formal Diagnosis: ICD; *antidepressant medication* |
| O'Neil *et al.* (2014)^18^ | *Dietary pattern*: Healthy, unhealthy diet | Dose-response, highest vs. lowest category | FFQ, DQI-I, OMD, YAQ | ﻿*Depressive & anxiety symptoms*: CBCL, SDQ, SMFQ, DSRS, EAS, PedsQL, SCARED; *formal diagnosis*: ICD-9 |
| Opie *et al.* (2015)^19^ | *Dietary pattern*: Diet high in fiber, vegetables, fruits, fish, red meat | No diet change control, waitlist control, treatment-as-usual, active control, placebo control, standard medical care | FFQ, ﻿Block 98 FFQ, ﻿FHQ, BFS, NCI FVS, ﻿Interview-based food survey, Food diaries, Food record, 24h recall, self-report dietary questionnaire, Daily food diary | *Depressive symptoms*: POMS, HADS, GDS, HAM-D, BDI, CGI, PHQ, BSI, TMAS, GWBS, CES-D |
| Khalid *et al.* (2017)^20^ | *Dietary patterns*: Healthy diet, unhealthy diet; *food groups*: Fruits, vegetables, cereal/grains, dairy, fish, fast food, snacks, sweets, meat | N/A | FFQ, YAQ, 3-day food records, controlled laboratory food measurement, eat healthy yes/no, kcal measure, food diary, eating behavior questionnaire, frequency of sweet foods and drinks, salty snacks, fast food | *Depressive symptoms*: CBC, SDQ, SMFQ, EuroQoL-youth, BDI-II, CBCL (4–18 years), CES-DC, DASS-21, EAS, DSRS, PedsQL; *formal diagnosis*: ICD 9 or 10 |

| ***Table H*** *continues on next page* |
| --- |

| ***Table H*** *continued* |
| --- |

| **Author, year** | **Intervention** | **Comparators** | **Dietary assessment** | **Outcome** |
| --- | --- | --- | --- | --- |
| Rahimlou *et al.* (2018)^21^ | *Dietary pattern:* Diet with high vs. low glycemic index, glycemic load | Highest vs. lowest category | FFQ, food record, DHQ | *Depressive symptoms*: POMS, CES-D, EPDS, GDS, HADS, Burnam scale |
| Altun *et al.* (2019)^22^ | *Dietary pattern:* Mediterranean diet | Highest vs. lowest category | FFQ, BDHQ, 24-h dietary recall, SDQ, interview | *Depressive symptoms*: CES-D, HADS, GDS, GHQ, K10, DASS-21, MADRS; *formal diagnosis*: DSM-IV; *antidepressant medication* |
| Arab *et al.* (2019)^23^ | *Dietary pattern:* DASH, Mediterranean diet, vegetable-based diet, glycemic load-based diet, Ketogenic diet, Paleo diet, Zone diet. | Highest vs. lowest category | FFQ, dietary record, 24-h recall | *Depressive symptoms*: POMS, BECK, HADS, AHIT, DASS |
| Tuck *et al.* (2019)^24^ | *Food groups:* Vegetables (& fruits) | Highest vs. lowest category | FFQ, food records, questions about daily intake, daily online survey, daily smartphone survey | *Depressive symptoms:* SF-36, CES-D, CIDI-SF, BDI-II |
| Glabska *et al.* (2020)^25^ | *Food groups:* Fruits, vegetables | Highest vs. lowest category | FFQ, rapid food screener, 3-day food records, self-report measures, 24-h recall, BDHQ, Chinese-FFQ | *Depressive symptoms:* CES-D, EURO-D scale, GHQ-12, GMS, SF-8, PHQ-9, DASS-21, GDS, DIGS, CIDI-SF, M-BDI, BDI, SF-36, GHQ-30 |
| Ljungberg *et al.* (2020)^26^ | *Dietary pattern:* Healthy diet (PANDiet, ﻿DQI-I, ﻿AHEI-2010, DASH, ﻿mPNNS-GS), Western diet, pro-inflammatory diet, micronutrients | Highest vs. lowest category, active control | N/A | N/A |
| *Abbreviations:* GI, glycemic index; GL, glycemic load; N/A, not known; RCTs, randomized controlled trials. *Outcome measures***:** AHIT, Atkins health indicator test; B-PHQ, Brief-Patient Health Questionnaire; BDI, Beck Depression Inventory, BDI-II, Beck Depression Inventory II; BDI-Y, Beck Depression Inventory for Youth; BSI, Brief Symptom Inventory; CBCL, Child Behavior Checklist (CBCL); CES-D, Center for Epidemiological Studies – Depression; CGI, Clinical Global Impression scale; CIDI, Composite International Diagnostic Interview (Version 2.1); CIDI-SF, Composite International Diagnostic Interview Short Form; DASS Depression Anxiety and Stress Scale; DIGS, ﻿Diagnostic Interview for Genetic Studies; DQI-P, Dietary Quality Index-Pregnancy; DSM-IV-TR, Diagnostic and Statistical Manual of Mental Disorders; DSM-IV, Diagnostic and Statistical Manual of Mental Disorders, Fourth Edition; DSRS, Depression Self-rating Scale for Children; EAS, Emotionality Activity and Sociability Questionnaire; EPDS, Edinburgh Post-partum Depression Scale; GDS, Geriatric Depression Scale; GHQ, General Health Questionnaire; GMS, Geriatric Mental State; GWBS, General Well-Being Schedule; HADS, Hospital Anxiety Depression Scale; HADS-D, Hospital Anxiety and Depression Scale for depression; HAM-D, Hamilton Depression scale; HANDLS, Healthy Aging in Neighborhoods of Diversity across the Life Span; HPL, Human Population Laboratory Depression Scale; HSCL, the Hopkins Symptom Checklist depression subscale; ICD, International Classification of Diseases; IDS, 30-item Inventory of Depressive Symptomatology Self-Report; K10, Kessler Psychological Distress Scale; M-BDI, Modified Beck Depression Inventory; M-CIDI, Munich-Composite International Diagnostic Interview; MADRS, Montgomery-Åsberg Depression Rating Scale; MFQ, Moods and Feelings Questionnaire; MHI-5, The five-item Mental Health Inventory; PedsQL, Pediatric Quality of Life Inventory; PHQ-9, Patient Health Questionnaire 9 item depression module; POMS, Profile of Mood States; SCARED, Screen for Child Anxiety Related Emotional Disorders; SCID-I/NP, Structured Clinical Interview for DSM-IV-TR Research Version, Non-Patient Edition; SCID, Structured Clinical Interview for Diagnostic and Statistical Manual of Mental Disorders, 4th edition; SDQ, Strengths and Difficulties Questionnaire; SF-36, Short-Form Health Survey; SMFQ, Short Mood and Feelings Questionnaire; TMAS, Taylor Manifest Anxiety Scale; WHO CIDI, WHO Composite International Diagnostic Interview; WPDS, Welsh Pure Depression sub-scale; Zung-SDS, Zung Self-Rating Depression Scale.  *Dietary indices:* AHEI, Alternative Healthy Eating Index; HEI, Healthy Eating Index; DASH, Dietary Approaches to Stop Hypertension; DII, Dietary Inflammatory Index; DQI-I, Diet Quality Index-International; mPNNS-GS, ﻿modified French Programme National Nutrition Sante’-Guideline Score; PANDiet, Probability of Adequate Nutrient Intake Dietary Score.  *Dietary measures:* BDHQ, Brief Diet History Questionnaire; BFS, Block Fat Screener; DHI, diet history interview; DHQ, Diet History Questionnaire; DQES v2, Dietary Questionnaire for Epidemiological Studies version 2; DQES, Dietary Questionnaire for Epidemiological Studies; DQES, Dietary questionnaire for epidemiological studies; DST, Dietary Screening Tool; FFQ, Food Frequency Questionnaire; ﻿FHQ, Food Habits Questionnaire; ﻿MDS, Mediterranean Diet Score; MeDi, Mediterranean Diet adherence score; NCI FVS, All Day NCI Fruit and Vegetable Screener; OMD, Optimized Mixed Diet; ﻿SDQ, Simple Dietary Questionnaire; USDA (AMPM), Automated Multiple Pass Method; YAQ; Youth and Adolescent Food Frequency Questionnaire. | | | | |

| **Table I.** Basic characteristics of included meta-analyses and systematic reviews (in chronological and alphabetical order) | | | | |
| --- | --- | --- | --- | --- |
| **Author, year** | ***N*** | ***k* (study types)** | **% Western Country** | **Mean follow-up** |
| *Meta-analyses* |  |  |  |  |
| Psaltopoulou *et al.* (2013)^1^ | 16,719 | 9 (8 cohort, 1 case-control) | 100% | 3.7y |
| Lai *et al.* (2014)^2^ | 128,923 | 20 (13 cross-sectional, 6 cohort, 1 case-control), but only 13 analyzed | 85.7% | 7.9y |
| Rahe *et al.* (2014)^3^ | 53,759 | 16 (9 cohort, 7 cross-sectional) | 81% | N/A |
| Li *et al.* (2015)^4^ | 150,278 | 26 (10 cohort, 16 cross-sectional) | 77% | N/A |
| Grosso *et al.* (2016)^5^ | 255,076 | 31 (13 cross-sectional, 15 cohort, 2 both) | 87% | ﻿2-3y |
| Liu *et al.* (2016)^6^ | 233,040 | 10 (4 cohort, 6 cross-sectional) | 50% | N/A |
| Li *et al.* (2017)^7^ | 117,229 | 21 (11 cohort, 6 cross-sectional, 4 case-control) | 43% | N/A |
| Molendijk *et al.* (2018)^8^ | 206,347 | 24 (cohort) | 93% | 7.9y |
| Saghafian *et al.* (2018)^9^ | 289,018 | 27 (16 cross-sectional, 9 cohort, 2 case-control) | 70.4% | N/A |
| Yang *et al.* (2018)^10^ | 109,764 | 10 (cohort) | 80% | 10y |
| Lassale *et al.* (2019)^11^ | 234,930 | 41 (20 cohort, 21 cross-sectional) | 82.2% | 8y |
| Nicolaou *et al.* (2019)^12^ | 23,026 | 6 (cohort) | 100% | N/A |
| Salari-Moghaddam *et al.* (2019)^13^ | 187,053 | 11 (6 cross-sectional, 2 cohort, 3 RCTs) | 53.8% | 10.3m |
| Shafiei *et al.* (2019)^14^ | 56,043 | 14 (7 cohort, 9 cross-sectional) | 100% | 9.2y |
| *Systematic reviews* |  |  |  |  |
| Murakami *et al.* (2010)^15^ | 120,935 | 34 (23 cross-sectional, 10 cohort, 1 case-control) | 79.4% | 6.8y |
| Quirk *et al.* (2013)^16^ | 53,770 | 25 (19 cross-sectional, 1 case-control, 5 cohort) | 80% | N/A |
| Sanhueza *et al.* (2013)^17^ | 57,980 | 11 (cohort) | 100% | 2 - 13y |
| O'Neil *et al.* (2014)^18^ | 82,779 | 12 (3 cohort, 9 cross-sectional) | 91.7% | 2 - 4y |
| Opie *et al.* (2015)^19^ | 4,015 | 17 (RCTs) | 88% | 10d - 36m |
| Khalid *et al.* (2017)^20^ | 109,533 | 20 (17 cross-sectional, 3 cohort) | 80% | N/A |
| Rahimlou *et al.* (2018)^21^ | 75,298 | 6 (3 cross-sectional, 3 cohort) | 33.3% | N/A |
| Altun *et al.* (2019)^22^ | 147,263 | 26 (7 cross-sectional, 13 cohort, 6 RCTs) | 88.5% | N/A |
| Arab *et al.* (2019)^23^ | 4,569 | 18 (4 observational, 14 RCTs) | 94.4% | 3.7m |
| Tuck *et al.* (2019)^24^ | 33,645 | 10 (9 observational, 1 RCT) | 90% | 1 y |
| Glabska *et al.* (2020)^25^ | N/A | 61 (observational) | 70.5% | N/A |
| Ljungberg *et al.* (2020)^26^ | 455,781 | 22 (17 cohort, 3 cross-sectional, 2 RCTs) | 100% | N/A |
| *Abbreviations*. d, days; *k*, total number of included studies; m, months; *N*, total number of participants; N/A, not avaliable; RCTs, randomized controlled trials, y, years. | | | | |

| **Table J.** Quantitative results of meta-analysis (in chronological and alphabetical order) | | | | | | | | |
| --- | --- | --- | --- | --- | --- | --- | --- | --- |
| **Author, year** | **Diet** | **Study design** | **ES Measure** | **ES** | **95% CI** | ***k*** | ***N*** |  |
| Psaltopoulou *et al.* (2013)^1^ | Mediterranean diet | Cohort, case-control | RR | 0.68 | 0.54, 0.86 | 9 | 17,175 |  |
| Lai *et al.* (2014)^2^ | Healthy diet | Cohort, cross-sectional | OR | 0.84 | 0.76, 0.92 | 13 | N/A |  |
|  | Western diet | Cohort, cross-sectional | OR | 1.17 | 0.97, 1.68 | 4 | N/A |  |
| Li *et al.* (2015)^4^ | Fish | Cohort | RR | 0.84 | 0.75, 0.94 | 10 | N/A |  |
|  |  | Cross-sectional | RR | 0.82 | 0.68, 1.00 | 16 | N/A |  |
| Grosso *et al.* (2016)^5^ | Fish | Cohort, cross-sectional | RR | 0.78 | 0.69, 0.89 | 18 | 107,098 |  |
| Liu *et al.* (2016)^6^ | Fruits | Cohort, cross-sectional | OR | 0.86 | 0.81, 0.91 | 10 | 227,852 |  |
|  | Vegetables | Cohort, cross-sectional | OR | 0.89 | 0.83, 0.94 | 8 | 218,699 |  |
| Li *et al.* (2017)^7^ | Healthy diet | Cohort, cross-sectional, case-control | OR | 0.64 | 0.57, 0.72 | 21 | 62,138 |  |
|  | Western-style diet | Cohort, cross-sectional, case-control | OR | 1.18 | 1.05, 1.34 | 17 | 40,975 |  |
| Molendijk *et al.* (2018)^8^ | High-quality diet | Cohort | OR | 0.77 | 0.69, 0.84 | 17 | 127,973 |  |
|  | Healthy food groups | Cohort | OR | 0.89 | 0.83, 0.95 | 18 | 147,011 |  |
|  | Unhealthy diet | Cohort | OR | 1.05 | ﻿0.99, 1.12 | 10 | 84,870 |  |
|  | Unhealthy food groups | Cohort | OR | 1.09 | 1.00, 1.19 | 7 | 97,632 |  |
|  | Neutral food groups | Cohort | OR | 0.91 | 0.84, 1.00 | 7 | 98,084 |  |
| Saghafian *et al.* (2018)^9^ | Fruits | Cohort | RR | 0.83 | 0.71, 0.98 | 6 | N/A |  |
|  |  | Cross-sectional | RR | 0.76 | 0.62, 0.92 | 6 | N/A |  |
|  | Vegetables | Cohort | RR | 0.86 | 0.75, 0.98 | 7 | N/A |  |
|  |  | Cross-sectional | RR | 0.75 | 0.62, 0.91 | 8 | N/A |  |
| Yang *et al.* (2018)^10^ | Fish | Cohort | RR | 0.89 | 0.80, 0.99 | 10 | 109,764 |  |
| Lassale *et al.* (2019)^11^ | Mediterranean diet | Cohort | OR | 0.67 | 0.55, 0.82 | 4 | 36,556 |  |
|  |  | Cross-sectional | OR | 0.66 | 0.23, 1.24 | 2 | 4,733 |  |
|  | DII | Cohort | OR | 0.76 | 0.63, 0.92 | 7 | 32,908 |  |
|  |  | Cross-sectional | OR | 0.64 | 0.45, 0.91 | 6 | 32,758 |  |
|  | DASH | Cohort | OR | 0.89 | 0.60, 1.31 | 4 | 14,051 |  |
|  |  | Cross-sectional | OR | 0.93 | 0.72, 1.21 | 4 | 6,421 |  |
|  | HEI | Cohort | OR | 0.76 | 0.57, 1.02 | 4 | 45,533 |  |
|  |  | Cross-sectional | OR | 0.53 | 0.38, 0.75 | 4 | 6,183 |  |

| ***Table J*** *continues on next page* |
| --- |
| ***Table J*** *continued* |

| **Author, year** | **Diet** | **Study design** | **ES Measure** | **ES** | **95% CI** | ***k*** | ***N*** |
| --- | --- | --- | --- | --- | --- | --- | --- |
| Nicolaou *et al.* (2019)^12^ | Mediterranean diet | Cohort | OR | 0.88 | 0.80–0.96 | 3 | 10,721 |
|  |  | Cross-sectional | OR | 0.87 | 0.84–0.91 | 6 | 23,107 |
|  | AHEI-2010 | Cohort | OR | 0.95 | 0.84–1.06 | 3 | 10,721 |
|  |  | Cross-sectional | OR | 0.93 | 0.88–0.98 | 6 | 23,107 |
|  | DASH | Cohort | OR | 0.90 | 0.84–0.97 | 3 | 10,721 |
|  |  | Cross-sectional | OR | 0.94 | 0.87–1.01 | 6 | 23,107 |
| Salari-Moghaddam *et al.* (2019)^13^ | Dietary GI | Cohort | HR | 1.13 | 1.02, 1.25 | 2 | 85,500 |
|  |  | Cross-sectional | OR | 1.01 | 0.84, 1.04 | 5 | 101,413 |
|  | Dietary GL | RCT | WMD | 0.66 | 0.28, 1.04 | 2 | 124 |
|  |  | Cross-sectional | OR | 0.93 | 0.84, 1.04 | 5 | 101,413 |
| Shafiei *et al.* (2019)^14^ | Mediterranean diet | Cohort | HR | 0.95 | 0.79, 1.16 | 4 | 37,806 |
|  |  | Cohort | β | -0.02 | -0.12, 0.11 | 3 | 17,252 |
|  |  | Cross-sectional | OR | 0.72 | 0.60, 0.87 | 9 | 35,873 |
| *Abbreviations:* AHEI, Alternative Healthy Eating Index; CI, confidence interval; DASH, Dietary Approaches to Stop Hypertension; DII, Dietary Inflammatory Index; ES, effect size; GI, glycemic index; GL, glycemic load; HEI, Healthy Eating Index; HR, hazard ratio; *k,* number of included studies; MDS, Mediterranean diet score; *N*, sample size; N/A, not available; OR, odds ratio; RCT, randomized controlled trial; RR, risk ratio; WMD, weighted mean difference. | | | | | | | |

| **Table K.** Qualitative results of systematic reviews (in chronological and alphabetical order) | |
| --- | --- |
| **Author, year** | **Results** |
| Murakami *et al.* (2010)^15^ | "Seven articles (two in men, one in women, and four in men and women combined) reported a significant inverse relation of fish intake with depressive symptoms, while nine (two in men, four in women, and three in men and women combined) observed no association." |
| Quirk *et al.* (2013)^16^ | "Our best-evidence analyses found limited evidence to support an association between traditional diets (Mediterranean or Norwegian diets) and depression. We also observed a conflicting level of evidence for associations between (i) a traditional Japanese diet and depression, (ii) a “healthy” diet and depression, (iii) a Western diet and depression, and (iv) individuals with depression and the likelihood of eating a less healthy diet." |
| Sanhueza *et al.* (2013)^17^ | "The review indicates that a diet including folate, omega-3 fatty acids, olive oil, fish, fruits, nuts and vegetables may have a protective effect against depression. On the other hand, there was some evidence indicating that diets containing processed foods, such as chocolate, refined grains, processed meat, whole-fat dairy products and fried foods, were associated with a higher risk of depression." |
| O'Neil *et al.* (2014)^18^ | "We found evidence of a significant, cross-sectional relationship between unhealthy dietary patterns and poorer mental health in children and adolescents. We observed a consistent trend for the relationship between good quality diet and better mental health and some evidence for the reverse. When including only the 7 studies deemed to be of high methodological quality, all but 1 of these trends remained." |
| Rahe *et al.* (2014)^3^ | "The available literature suggests a protective effect of healthy and Mediterranean patterns, as well as a potential positive association of Western patterns and depression. However, comparison of the included studies was difficult, due to differences in relevant study characteristics and methodological limitations." |
| Opie *et al.* (2015)^19^ | "Compared with a control condition, almost half (47 %) of the studies observed significant effects on depression scores in favor of the treatment group. The remaining studies reported a null effect." |
| Khalid *et al.* (2017)^20^ | "Despite some contradictory results, overall there was support for an association between healthy dietary patterns or consumption of a high-quality diet and lower levels of depression or better mental health. Similarly, there was a relationship between unhealthy diet and consumption of low-quality diet and depression or poor mental health. However, where significant relationships were reported, effect sizes were small." |
| Rahimlou *et al.* (2018)^21^ | "According to the results obtained, it seems that low GI carbohydrates are associated with reduced odds of depression, but association between GL and odds of depression was controversial." |
| Altun *et al.* (2019)^22^ | "﻿The majority (85%) of observational studies support the evidence that the Mediterranean dietary pattern is associated with reductions in depressive incidence and all intervention studies echoed these findings." |
| Arab *et al.* (2019)^23^ | ﻿"Although there are not consistent findings between studies, it seems that DASH, vegetable-based, glycemic load-based, ketogenic and Paleo diets could improve mood more than the others. Further studies are needed to assess such relationship in a longer period to draw a firm link between diet and mood." |
| Tuck *et al.* (2019)^24^ | ﻿"﻿Where studies explored the independent effects of fruit and vegetable consumption on psychological health (n = 3), 2 reported a preferential effect of vegetables (compared with fruit) on psychological well-being, whereas 1 reported a superior effect of fruit intake on odds reduction of symptoms of depression. More broadly, there was evidence that consuming the recommended amount of F&V (and exceeding this) was associated with increased psychological well-being. However, the effects of F&V consumption on mental health symptoms were inconsistent." |
| Glabska *et al.* (2020)^25^ | "The most prominent results indicated that high total intake of fruits and vegetables, and some of their specific subgroups including berries, citrus, and green leafy vegetables, may promote higher levels of optimism and self-efficacy, as well as reduce the level of psychological distress, ambiguity, and cancer fatalism, and protect against depressive symptoms. However, it must be indicated that the studies included were conducted using various methodologies and in different populations, so their results were not always sufficiently comparable, which is a limitation." |
| Ljungberg *et al.* (2020)^26^ | ﻿"High adherence to dietary recommendations, anti-inflammatory diet, fish consumption, exclusion of processed foods, and adequate intake of folic acid, magnesium different fatty acids, were associated with a reduced risk of mental illness." |
| *Abbreviations:* DASH, Dietary Approaches to Stop Hypertension; GI, glycemic index; GL, glycemic load. | |

References

1 Psaltopoulou T, Sergentanis TN, Panagiotakos DB, Sergentanis IN, Kosti R, Scarmeas N. Mediterranean diet, stroke, cognitive impairment, and depression: A meta-analysis. *Ann Neurol* 2013; **74**: 580–591.

2 Lai JS, Hiles S, Bisquera A, Hure AJ, McEvoy M, Attia J. A systematic review and meta-analysis of dietary patterns and depression in community-dwelling adults. *Am J Clin Nutr* 2014; **99**: 181–97.

3 Rahe C, Unrath M, Berger K. Dietary patterns and the risk of depression in adults: A systematic review of observational studies. *Eur J Nutr* 2014; **53**: 997–1013.

4 Li F, Liu X, Zhang D. Fish consumption and risk of depression: A meta-analysis. *J Epidemiol Community Health* 2015; **70**: 299–304.

5 Grosso G, Micek A, Marventano S, Castellano S, Mistretta A, Pajak A *et al.* Dietary n-3 PUFA, fish consumption and depression: A systematic review and meta-analysis of observational studies. *J Affect Disord* 2016; **205**: 269–281.

6 Liu X, Yan Y, Li F, Zhang D. Fruit and vegetable consumption and the risk of depression: A meta-analysis. *Nutrition* 2016; **32**: 296–302.

7 Li Y, Lv M-R, Wei Y-J, Sun L, Zhang J-X, Zhang H-G *et al.* Dietary patterns and depression risk: A meta-analysis. *Psychiatry Res* 2017; **253**: 373–382.

8 Molendijk M, Molero P, Ortuño Sánchez-Pedreño F, Van der Does W, Angel Martínez-González M. Diet quality and depression risk: A systematic review and dose-response meta-analysis of prospective studies. *J Affect Disord* 2018; **226**: 346–354.

9 Saghafian F, Malmir H, Saneei P, Milajerdi A, Larijani B, Esmaillzadeh A. Fruit and vegetable consumption and risk of depression: Accumulative evidence from an updated systematic review and meta-Analysis of epidemiological studies. *Br J Nutr* 2018; **119**: 1087–1101.

10 Yang Y, Kim Y, Je Y. Fish consumption and risk of depression: Epidemiological evidence from prospective studies. *Asia-Pacific Psychiatry* 2018; **10**: e12335.

11 Lassale C, Batty GD, Baghdadli A, Jacka F, Sánchez-Villegas A, Kivimäki M *et al.* Healthy dietary indices and risk of depressive outcomes: a systematic review and meta-analysis of observational studies. *Mol Psychiatry* 2019; **24**: 965–986.

12 Nicolaou M, Colpo M, Vermeulen E. Association of a priori dietary patterns with depressive symptoms: a harmonized meta-analysis of observational studies. *Psychol Med* 2019.in press.

13 Salari-Moghaddam A, Saneei P, Larijani B, Esmaillzadeh A. Glycemic index, glycemic load, and depression: a systematic review and meta-analysis. *Eur J Clin Nutr* 2019; **73**: 356–365.

14 Shafiei F, Salari-Moghaddam A, Larijani B, Esmaillzadeh A. Adherence to the mediterranean diet and risk of depression: A systematic review and updated meta-analysis of observational studies. *Nutr Rev* 2019; **77**: 230–239.

15 Murakami K, Sasaki S. Dietary intake and depressive symptoms: A systematic review of observational studies. *Mol Nutr Food Res* 2010; **54**: 471–488.

16 Quirk SE, Williams LJ, O’Neil A, Pasco JA, Jacka FN, Housden S *et al.* The association between diet quality, dietary patterns and depression in adults: a systematic review. *BMC Psychiatry* 2013; **13**: 175.

17 Sanhueza C, Ryan L, Foxcroft DR. Diet and the risk of unipolar depression in adults: Systematic review of cohort studies. *J Hum Nutr Diet* 2013; **26**: 56–70.

18 O’Neil A, Quirk SE, Housden S, Brennan SL, Williams LJ, Pasco JA *et al.* Relationship between diet and mental health in children and adolescents: A systematic review. *Am J Public Health* 2014; **104**: e31–e42.

19 Opie RS, O’Neil A, Itsiopoulos C, Jacka FN. The impact of whole-of-diet interventions on depression and anxiety: A systematic review of randomised controlled trials. *Public Health Nutr* 2015; **18**: 2074–2093.

20 Khalid S, Williams CM, Reynolds SA. Is there an association between diet and depression in children and adolescents? A systematic review. *Br J Nutr* 2016; **116**: 2097–2108.

21 Rahimlou M, Morshedzadeh N, Karimi S, Jafarirad S. Association between dietary glycemic index and glycemic load with depression: a systematic review. *Eur J Nutr* 2018; **57**: 2333–2340.

22 Altun A, Brown H, Szoeke C, Goodwill AM. The Mediterranean dietary pattern and depression risk: A systematic review. *Neurol Psychiatry Brain Res* 2019; **33**: 1–10.

23 Arab A, Mehrabani S, Moradi S, Amani R. The association between diet and mood: A systematic review of current literature. *Psychiatry Res* 2019; **271**: 428–437.

24 Tuck N-J, Farrow C, Thomas JM. Assessing the effects of vegetable consumption on the psychological health of healthy adults: a systematic review of prospective research. *Am J Clin Nutr* 2019; **110**: 196–211.

25 Głąbska D, Guzek D, Groele B, Gutkowska K. Fruit and Vegetable Intake and Mental Health in Adults: A Systematic Review. *Nutrients* 2020; **12**: 115.

26 Ljungberg T, Bondza E, Lethin C. Evidence of the Importance of Dietary Habits Regarding Depressive Symptoms and Depression. *Int J Environ Res Public Health* 2020; **17**: 1616.
