# Supplementary material for: Impact of review method on the conclusions of clinical reviews: A systematic review on dietary interventions in depression as a case in point
Source: PLoS One. 2020 Sep 16;15(9):e0238131. doi: 10.1371/journal.pone.0238131 (PMC7494108; doi:10.1371/journal.pone.0238131)
Supplement: S3 Table — (DOCX) [file pone.0238131.s006.docx]

| **Table I.** Reported conclusions of included studies with classified strength (in chronological and alphabetical order) | | |
| --- | --- | --- |
| **Author, year** | **Conclusions** | **Strength** |
| *Meta-analyses* |  |  |
| Psaltopoulou *et al.* (2013)^1^ | *Abstract*: "﻿Adherence to a Mediterranean diet may contribute to the prevention of a series of brain diseases; this may be of special value given the aging of Western societies." [I]  *Discussion*: “In conclusion, given the limited availability of pharmaceutical agents to treat cognitive impairment, cognitive decline, and stroke, one could argue for the importance of preventive measures, such as a healthy dietary regime, to diminish the risk of mild and advanced cognitive decline, AD, depression, and stroke.” | Moderate  Moderate |
| Lai *et al.* (2014)^2^ | *Abstract*: "﻿The results suggest that high intakes of fruit, vegetables, fish, and whole grains may be associated with a reduced depression risk. However, more high-quality randomized controlled trials and cohort studies are needed to confirm this finding, specifically the temporal sequence of this association." [I]  *Discussion*: “In conclusion, there is a need for more RCTs and prospective cohort studies to clarify whether true causal associations exist between dietary patterns and depression.” | Weak  Moderate |
| Rahe *et al.* (2014)^3^ | *Abstract*: "﻿There are indications that dietary patterns may have influence on the onset of depression, but no firm conclusion can be drawn at this point. Further research is needed to clarify the diet–depression relationship, preferably in the form of methodological strong prospective studies using more homogeneous methods." [I]  *Discussion*: “﻿In summary, the available literature suggests a possible association between dietary patterns and depression: Healthy and Mediterranean dietary patterns seem to be associated with lower odds of depression, whereas Western dietary patterns may be associated with higher odds of depression. But with respect to the major differences in study characteristics, the high level of heterogeneity and some methodological limitations of the included studies, no firm conclusion can be drawn at this point.” | Weak  Weak |
| Li *et al.* (2015)^4^ | *Abstract*: "﻿This meta-analysis indicates that high-fish consumption can reduce the risk of depression." [I]  *Discussion*: “In conclusion, higher fish consumption may be beneficial in the primary prevention of depression.” | Moderate  Moderate |
| Grosso *et al.* (2016)^5^ | ﻿*Abstract*: “The present analysis supports the hypothesis that dietary n-3 PUFA intake are associated with lower risk of depression.”  *Discussion*: "In conclusion, a comprehensive analysis of available observational studies supports the hypothesis that dietary n-3 PUFA intake, especially derived by fish, decrease the risk of depression. Heterogeneity among studies was found, weakening final conclusions". [I] | Moderate  Moderate |
| Liu *et al.* (2016)^6^ | *Abstract*: "﻿This meta-analysis indicated that fruit and vegetable consumption might be inversely associated with the risk of depression, respectively." [I]  *Discussion*: “In summary, the present meta-analyses indicated that fruit and vegetable intake was inversely associated with the risk of depression.” | Weak  Weak |
| Li *et al.* (2017)^7^ | ﻿ *Abstract*: "The results of this meta-analysis suggest that healthy pattern may decrease the risk of depression, whereas western-style may increase the risk of depression. However, more randomized controlled trails and cohort studies are urgently required to confirm this finding." [I]  *Discussion*: “In conclusion, the present meta-analysis suggested that the healthy dietary pattern was associated with a decreased risk of depression, whereas Western-style/unhealthy dietary pattern was associated with an increased risk of depression. Our findings add to the evidence of the role of dietary patterns in the prevention and management of depression. Therefore, it makes sense to elucidate the potential association between dietary patterns and depression risk and provide a scientific rational for formulating dietary guidelines. Further studies are urgently required to confirm the causal relationship between dietary patterns and the risk of depression” | Weak  Moderate |

| ***Table I*** *continues on next page* |
| --- |

| ***Table I*** *continued* |  |  |
| --- | --- | --- |
| Molendijk *et al.* (2018)^8^ | *Abstract*: "﻿There is evidence that a higher quality of a diet is associated with a lower risk for the onset of depressive symptoms, but not all available results are consistent with the hypothesis that diet influences depression risk. Prospective studies that control for relevant confounders such as obesity incidence and randomized controlled prevention trials are needed to increase the validity of findings in this field." [I]  *Discussion*: “We conclude that dietary quality seems to be associated with the incidence of depressive symptoms but also that much data exists that attenuates the conclusion that diet is crucially involved in depressive illness onset.” | Weak  Weak |
| Saghafian *et al.* (2018)^9^ | *Abstrac*t: "﻿This meta-analysis of observational studies provides further evidence that fruit and vegetable intake was protectively associated with depression." [I]  *Discussion*: “This meta-analysis of observational studies provides further evidence that fruit and vegetables intake was protectively associated with depression.” | Moderate  Moderate |
| Yang *et al.* (2018)^10^ [II] | *Abstract:* “Our findings provide quantitative evidence for a modest inverse association between fish or omega‐3 fatty acid intake and risk of depression, especially in women. These findings from the observational studies need to be confirmed through large randomized clinical trials of fish consumption or omega‐3 fatty acid intake and risk of depression.”  *Discussion:* “In conclusion, our findings provide quantitative evidence for a modest inverse association between fish or omega‐3 fatty acid intake and risk of depression, especially in women. These findings from the observational studies need to be confirmed through large randomized clinical trials of fish consumption or omega‐3 fatty acid intake and risk of depression.” | Moderate  Moderate |
| Lassale *et al.* (2019)^11^ | *Abstrac*t: "To conclude, adhering to a healthy diet, in particular a traditional Mediterranean diet, or avoiding a pro-inflammatory diet appears to confer some protection against depression in observational studies." [I]  *Discussion:* “Our review shows that there is observational evidence to suggest that both adhering to a healthy diet, in particular a traditional Mediterranean diet, and avoiding a pro-inflammatory diet is associated with reduced risk of depressive symptoms or clinical depression.” | Moderate  Moderate |
| Nicolaou *et al.* (2019)^12^ | *Abstract*: "Population-scale observational evidence indicates that adults following a healthy dietary pattern have fewer depressive symptoms and lower risk of developing depressive symptoms." [I]  *Discussion*: “Based on the findings from this harmonised meta-analysis of observational studies, we conclude that greater adherence to a healthy dietary pattern is associated with fewer depressive symptoms and a lower risk of developing depressive symptoms over time.” | Moderate  Moderate |
| Salari-Moghaddam *et al.* (2019)^13^ | *Abstract: “*Summarizing earlier findings, we found no significant association between either dietary GI or GL and odds of depression in cross-sectional studies. However, a significant positive association was observed between dietary GI and depression in cohort studies. In addition, a significant effect of a high-GL diet consumption on risk of depression was seen in clinical trials.”  *Discussion:* "﻿In conclusion, summarizing earlier findings, we found no significant association between dietary GI and GL, and odds of depression in cross-sectional studies. However, we found a significant positive association between dietary GI and depression in cohort studies. In addition, we found a significant change in depression score after consumption of a high-GL diet in clinical trials. Due to limited information in this field, further studies are required to reach a definite conclusion." [I] | Weak  Weak |
| Shafiei *et al.* (2019)^14^ | *Abstract*: "﻿The analysis of cohort studies revealed no significant association between adherence to the Mediterranean diet and risk of depression. However, an inverse significant association was found between adherence to the Mediterranean diet and odds of depression in cross-sectional studies." [I]  *Discussion*: “This systematic review and updated meta-analysis revealed no significant association between adherence to the Mediterranean diet and risk of depression when cohort studies were analyzed. However, analysis of cross-sectional studies showed an inverse significant association between adherence to the Mediterranean diet and odds of depression.” | Weak  Weak |

***Table I*** *continues on next page*

| ***Table I*** *continued* |  |  |
| --- | --- | --- |
| **Author, year** | **Conclusions** | **Strength** |
| *Systematic reviews* |  |  |
| Murakami *et al.* (2010)^15^ | *Abstract:* "Most studies found no association between dietary variables and depressive symptoms. However, most studies included at least one important methodological limitation, such as no inference for causality, unreliable or rough assessment of diet or depressive symptoms, inadequate treatment of potential confounding factors, and ignorance of the possible mediating or confounding influence of other dietary variables. Further evidence from well-designed observational studies is required to confirm or refute the association between dietary intake and depressive symptoms in free- living settings." [I]  *Discussion*: “﻿Given that the relationship between eating and mood is quite complex, and that food intake pattern reflects complex interrelations and interactions among the individual, the culture, and the society in which people live [2], firm conclusions regarding the association between dietary intake and depressive symptoms cannot be drawn at present.” | Weak    Weak |
| Quirk *et al.* (2013)^16^ | *Abstract:* "To our knowledge, this is the first review to synthesize and critically analyze evidence regarding diet quality, dietary patterns and depression. Further studies are urgently required to elucidate whether a true causal association exists." [I]  *Discussion*: “In summary, this systematic review provides a critical summary of the current evidence regarding diet quality and depression, a relatively new field of enquiry. To elucidate whether true causal associations exist between diet and depression, further research is urgently required.” | Moderate  Moderate |
| Sanhueza *et al.* (2013)^17^ | *Abstract*: "At the study level, weaknesses in the assessment of exposure and outcome may have introduced bias. Most studies investigated a cohort subgroup that may have resulted in selection bias. At the review level, there is a risk of publication bias and, in addition, narrative analyses are more prone to subjectivities than meta-analyses. Diet may potentially influence the risk of depression, although the evidence is not yet conclusive. […] Robust prospective cohort studies specially designed to study the association between diet and depression risk are needed." [I]  *Discussion*: “﻿Broadly speaking, the current literature, although sparse and with some methodological problems, does suggest that nutritional variables may have a role in the aetiology of unipolar depression, that certain nutrients and/or foods may be risk factors for this condition and, subsequently, that dietary modifications may help prevent unipolar depressive disorders. However, there is no strong or compelling consistency in the findings across different nutrients, food types and dietary patterns to enable drawing a firm conclusion that diet and nutritional factors can lead to depression. ﻿Although the evidence precludes a firm conclusion, the results from the included studies tentatively indicate that a dietary pattern including fruits, vegetables, fish, olive oil, nuts and legumes may protect against depression. On the other hand, a high consumption of processed food and sugary products may increase the likelihood of depression. Given the evidence, these are, at best, weak indications, and so more research is needed to confirm or refute this initial finding.” | Weak  Weak |
| O'Neil *et al.* (2014)^18^ | ﻿ *Abstract*: "Findings highlight the potential importance of the relationship between dietary patterns or quality and mental health early in the life span." [I]  *Discussion*: “Despite a paucity of data, our findings highlight the potential importance of the relationship between dietary patterns or quality and mental health early in the life span. Prospective and intervention studies are now required to improve the level of evidence.” | Moderate  Moderate |
| Opie *et al.* (2015)^19^ | *Abstract*: "﻿Although there was a high level of heterogeneity, we found some evidence for dietary interventions improving depression outcomes. However, as only one trial specifically investigated the impact of a dietary intervention in individuals with clinical depression, appropriately powered trials that examine the effects of dietary improvement on mental health outcomes in those with clinical disorders are required." [I]  *Discussion*: “The present review of RCTs has demonstrated that dietary intervention studies have the potential to achieve improved depression scores. ﻿The paper provides some insight into the key components that are likely to achieve improved depression outcomes. Appropriately powered RCT evaluating the impact of dietary improvement on mental health outcomes in those with clinical disorders are required.” | Moderate  Moderate |

***Table I*** *continues on next page*

| ***Table I*** *continued* |  |  |
| --- | --- | --- |
| Khalid *et al.* (2017)^20^ | *Abstract*: “Despite some contradictory results, overall there was support for an association between healthy dietary patterns or consumption of a high-quality diet and lower levels of depression or better mental health. Similarly, there was a relationship between unhealthy diet and consumption of low-quality diet and depression or poor mental health. However, where significant relationships were reported, effect sizes were small. Future research on the relationship between diet and mental health in young people should use more clearly defined constructs to define diet and include or control for important confounders.”  *Discussion*: "﻿Research regarding dietary pattern or diet quality and its association with mental health in children and adolescents is at an early stage. This review highlighted some conceptual and methodological problems that, if not addressed, will impede future research and public health interventions. It is therefore essential to make sure that further methodological problems are minimized to at least establish the strength of any association between diet and mental health." [I] | Weak    Weak |
| Rahimlou *et al.* (2018)^21^ | *Abstract*: "﻿Overall, the findings indicated that a diet with lower dietary glycemic index may be effective to reduce the risk or risk of depression."  *Discussion*: “This systematic review provided evidence for the association between GL and GI with depression risk. It seems that there was a strong positive association for GI and depression risk. However, the inverse association observed for GL is currently controversial and further high-quality studies are needed to confirm these findings.” [I] | Moderate  Moderate |
| Altun *et al.* (2019)^22^ | ﻿*Abstract*: "Modifying diet provides a potential treatment for depression which procures few side effects, lessens disease progression and demonstrates a cost-effective measure that can be implemented globally. Present research has found that more objective measures are necessary to define the Mediterranean diet and highlights the need for longitudinal studies and clinical trials for future research." [I]  *Discussion*: “The Mediterranean dietary pattern offers a potential alternative or useful adjunct to current treatments in place for depression, which procures few side effects, lessens disease severity and demonstrates a cost-effective measure that can be implemented globally.” | Strong  Strong |
| Arab *et al.* (2019)^23^ | *Abstract*: "Although there are not consistent findings between studies, it seems that DASH, vegetable-based, glycemic load-based, ketogenic and Paleo diets could improve mood more than the others. Further studies are needed to assess such relationship in a longer period to draw a firm link between diet and mood." [I]  *Discussion*: “According to all mentioned above, we found that diet could improve the mood states, but differences between the diets should be taken into account. Although there is not a consistent finding between studies, it seems that DASH, vegetable-based, glycemic load-based, ketogenic and Paleo diets could improve mood more than the others. Further studies are needed to assess such relationship in a longer period to draw a firm link between diet and mood.” | Moderate  Moderate |
| Tuck *et al.* (2019)^24^ [II] | ﻿*Abstract*: "Increased F&V consumption has a positive effect on psychological well-being and there appears to be a preferential effect of vegetables (compared with fruit) from the limited data examined. The effect of F&V intake on mental health is less clear and, at present, there are no clear data to support a preferential effect of vegetable intake on mental health outcomes. ﻿Hence, additional research is warranted to investigate the influence of vegetables, compared with fruit, on psychological health in order to inform nutrition-based interventions.”  *Discussion*: “In conclusion, this review identified that increased F&V consumption has a positive impact on psychological health. [...] However, it must be noted that these conclusions are drawn from limited data. The effect of F&V consumption on mental health is less clear, hence, further work is required, particularly RCT studies that delineate the effects of fruit and vegetables.” | Moderate  Moderate |
| Glabska *et al.* (2020)^25^ [II] | ﻿*Abstract*: "Taken together, it can be concluded that fruits and/or vegetables, and some of their specific subgroups, as well as processed fruits and vegetables, seems to have a positive influence on mental health, as stated in the vast majority of the included studies.”  *Discussion*: “The vast majority of the included studies indicated that the intake of fruits and/or vegetables and their specific subgroups, as well as processed fruits and vegetables, seems to have a positive influence on mental health.” | Moderate  Moderate |

***Table I*** *continues on next page*

| ***Table I*** *continued* | |  |  |
| --- | --- | --- | --- |
| **Author, year** | **Conclusions** | | **Strength** |
| Ljungberg *et al.* (2020)^26^ [II] | | ﻿*Abstract*: “The result showed that high adherence to dietary recommendations; avoiding processed foods; intake of anti-inflammatory diet; magnesium and folic acid; various fatty acids; and fish consumption had a [protective effect against] depression. ﻿Further research is needed to strengthen a causal relationship and define evidence-based strategies to implement in prevention and treatment by public healthcare." *(we corrected the typo in square brackets in accordance with the authors)*  *Discussion*: “The diet may have a significant effect on preventing and treating depression for the individual.” | Moderate  Moderate |
| *Narrative reviews* | |  |  |
| McGrath-Hanna *et al.* (2003)^27^ | | *Abstract*: “Studies in non-circumpolar peoples have shown that diet can have profound effects on neuronal and brain development, function, and health. Therefore, we hypothesize that diet is an important risk factor for mental health in circumpolar peoples.”  *Discussion*: “﻿We hypothesize that diet is an important risk factor for mental health in circumpolar peoples. As we reviewed here, the diet of circumpolar people has changed considerably from a traditional diet high in omega-3 fatty acids and antioxidants, to a Western-style diet high in carbohydrates and saturated fat. Several lines of evidence reveal that omega-3 fatty acids and other nutrients that are rich in traditional diets are beneficial for mental health and that depletion of omega-3 fatty acids are associated with increased levels of depression and possibly suicide. The combined decline in mental health and the disappearance of traditional diets in circumpolar peoples makes a direct connection between diet and mental health in these people a very real possibility." [I] | Moderate  Moderate |
| Bamber *et al.* (2007)^28^ [II] | | *Abstract*: “This is important, as improving understanding of the role of diet in mental health and promotion of appropriate dietary practices could significantly reduce the personal and social impact of depression in young people”  *Discussion*: "However, it is not yet clear what characteristics of adolescent depression, such as degree of severity or duration of illness, are associated with a positive response to dietary intervention, and more research is needed to clarify this. Overall, there is emerging evidence for an association between low dietary intakes of certain nutrients and certain eating behaviour patterns with the occurrence of common forms of emotional and behavioural disorders across all ages. However, much more research is needed on specific disorders and with young people." | Moderate  Moderate |
| Low Dog (2010)^29^ | | *Abstract*: None  *Discussion*: "Given what we know so far, a modified low-glycemic load, Mediterranean style-diet appears to be optimal for mood as well as overall health." [I] | -  Moderate |
| Jacka & Berk (2012)^30^ | | *Abstract*: "Recent evidence suggests that poor diet and a lack of exercise contribute to the genesis and course of depression. While studies examining dietary improvement as a treatment strategy in depression are lacking, epidemiological evidence clearly points to diet quality being of importance to the risk of depression." [I]  *Discussion*: “Given the strength of the observational data, this is an area of considerable therapeutic promise.” | Strong  Strong |
| Jacka *et al.* (2012)^31^ | | *Abstract*: "On the basis of this most recent evidence, we consequently argue for the inclusion of depression and anxiety in the ranks of the high prevalence noncommunicable diseases influenced by habitual lifestyle practices." [I]  *Discussion*: ﻿“We hereby present the case for inclusion of the common mental disorders under the noncommunicable diseases umbrella, based on the recent identification of lifestyle as a modifiable risk factor…” | Strong  Strong |

***Table I*** *continues on next page*

| ***Table I*** *continued* |  |  |
| --- | --- | --- |
| **Author, year** | **Conclusions** | **Strength** |
| Berk *et al.* (2013)^32^ | *Abstract*: "…﻿while the new epidemiological evidence supports the contention that diet is a risk factor for depression; good quality diets appear protective and poor diets increase risk." [I]  *Discussion*: “…poor diet is now emerging as a risk factor for depression, and early data hint that dietary modification may be of therapeutic value.” | Strong  Strong |
| Manosso *et al.* (2013)^33^ | *Abstract*: None  *Discussion*: "[This review] discusses that a proper diet may decrease the risk of developing depressive symptoms, whereas an inadequate diet (e.g. rich in sugar and refined foods) may increase the risk of depression." [I] | -  Moderate |
| Sanchez-Villegas *et al.* (2013)^34^ | *Abstract*: "﻿Only a few cohort studies have analyzed the relationship between overall dietary patterns, such as the Mediterranean diet, and primary prevention of depression. They have found similar results to those obtained for the role of this dietary pattern in cardiovascular disease. To confirm the findings obtained in these initial cohort studies, we need further observational longitudinal studies with improved methodology, as well as large randomized primary prevention trials, with interventions based on changes in the overall food pattern, that include participants at high risk of mental disorders." [I]  *Discussion*: “Although a few prospective cohort studies have analyzed the role of dietary patterns on depression risk, their contributions are still scarce. Further observational studies with improved methodology (including repeated measurements of diet, better validation of measuring instruments, longer follow-up periods, larger sample sizes and adequate control of confounders) as well as large randomized primary prevention trials with interventions based on changes in the overall food pattern and including participants at high risk of mental disorders are necessary to confirm the findings obtained in these initial studies.” | Weak  Weak |
| Sarris *et al.* (2014)^35^ | *Abstract*: “There is now compelling evidence that a range of lifestyle factors are involved in the pathogenesis of depression.”  *Discussion*: "While these emerging data provide potential validity for the role of dietary and nutritional factors in the genesis of depression and its management, it should be acknowledged that currently, there is minimal evidence for dietary modulation as a treatment for depression." [I] | Strong  Moderate |
| LaChance & Ramsey (2015)^36^ | *Abstract*: “This article will review the evidence linking dietary pattern to brain-based illnesses and provide an overview of the mechanisms that underlie the association between brain health and the food we eat. Considerations for dietary intervention will be discussed including encouraging a shift towards a traditional or whole foods dietary pattern.”  *Discussion*: "﻿Therefore, it appears that dietary pattern can influence mental health through a number of mechanisms." [I] | Moderate  Moderate |
| Lang *et al.* (2015)^37^ | *Abstract*: "﻿In this context several healthy foods such as olive oil, fish, fruits, vegetables, nuts, legumes, poultry, dairy and unprocessed meat have been inversely associated with depression risk and even have been postulated to improve depressive symptoms. In contrast, unhealthy western dietary patterns including the consumption of sweetened beverage, refined food, fried food, processed meat, refined grain, and high fat diary, biscuits, snacking and pastries have been shown to be associated with an increased risk of depression in longitudinal studies. However, it is always difficult to conclude a real prospective causal relationship from these mostly retrospective studies as depressed individuals might also change their eating habits secondarily to their depression." [I]  *Discussion*: “However, nutritional interventions for major depression have not been studied extensively yet and there are only pilot studies available. Therefore, to the knowledge of the author, there is no high level evidence (large prospective RCTs, meta-analyses) for an effective nutritional intervention for the treatment of major depression.” | Weak    Weak |

***Table I*** *continues on next page*

| ***Table I*** *continued* | |  |  |
| --- | --- | --- | --- |
| **Author, year** | **Conclusions** | | **Strength** |
| Sarris *et al.* (2015)^38^ | *Abstract*: “We advocate recognition of diet and nutrition as central determinants of both physical and mental health.”  *Discussion*: "﻿Diet and nutrition offer key modifiable targets for the prevention of mental disorders, having a fundamental role in the promotion of mental health." [I] | | Strong  Strong |
| Jacka (2017)^39^ | *Abstract*: "A consistent evidence base from the observational literature confirms that the quality of individuals' diets is related to their risk for common mental disorders, such as depression. This is the case across countries and age groups. Moreover, new intervention studies implementing dietary changes suggest promise for the prevention and treatment of depression. [...] On the other hand, the field is currently limited by a lack of data and methodological issues such as heterogeneity, residual confounding, measurement error, and challenges in measuring and ensuring dietary adherence in intervention studies." [I]  *Discussion*: “The emerging field of Nutritional Psychiatry offers promise for a new approach to both the prevention and treatment of disorders that account for the leading disability burden globally.” | | Moderate  Moderate |
| Libuda *et al.* (2017)^40^ | *Abstract*: "In Beobachtungsstudien erwiesen sich traditionelle Ernährungsmuster wie die mediterrane Ernährung als protektiv für die seelische Gesundheit." [I]  *Discussion*: “Bisherige Ergebnisse aus Beobachtungsstudien deuten potenzielle Zusammenhänge zwischen einem gesunden Ernährungsmuster und einem geringeren Risiko für Depressionen an. Allerdings ist die Anzahl an Beobachtungsstudien insgesamt gering und besteht zu einem großen Teil aus Querschnittsanalysen. Für eine Einschätzung des Potenzials eines gesunden Ernährungsmuster für die Prävention von Depressionen werden Ergebnisse aus prospektiven Kohortenstudien mit longitudinaler Datenanalyse und ausreichender statistischer Power (d. h. Anzahl an Probanden, die eine Depression entwickeln) benötigt” | | Moderate  Weak |
| Opie *et al.* (2017)^41^ | ﻿*Abstract*: "Although there are a number of gaps in the scientific literature to date, existing evidence suggests that a combination of healthful dietary practices may reduce the risk of developing depression. It is imperative to remain mindful of any protective effects that are likely to come from the cumulative and synergic effect of nutrients that comprise the whole-diet, rather than from the effects of individual nutrients or single foods. As the body of evidence grows from controlled intervention studies on dietary patterns and depression, these recommendations should be modified accordingly." [I]  *Discussion*: “Although there are a number of important gaps in the scientific literature to date, existing evidence suggests that a combination of healthful dietary practices may reduce the risk of developing depression.” | | Moderate  Moderate |
| Stevenson, (2017)^42^ | *Abstract*: "Diet may be a causal contributor to depression [...]."[I]  *Discussion*: “In summary, there is support for a link between diet and depression. Lai et al.’s (2014) meta-analysis suggests reduced depression risk is associated with a healthier diet, and that a Western-style diet may increase risk for depressed mood. The former finding is also supported by 3/5 of the more recent studies that meet the same criteria as those used in the meta-analysis. Such findings may be filtering back to physicians, as at least two studies have reported evidence of ‘reverse causality,’ with improved diet in those with some form of depression (Jacka et al., 2015; Rahe et al., 2015). Whether nutritionally poor diets cause depression (and whether healthy diets are recuperative or preventative) is not yet established, and it will require interventional studies to assess causation.” | | Moderate  Weak |
| Brietzke *et al.* (2018)^43^ | *Abstract*: "﻿Preclinical studies, case reports and case series have demonstrated antidepressant and mood stabilizing effects of KD, however, to date, no clinical trials for depression or bipolar disorder have been conducted.” [I]  *Discussion*: “Ketogenic diet opens a new avenue for investigation of diet as a potential therapeutic intervention in mood disorders, offering a possibility to evaluate its effects under specific domains of psychopathology, such as anhedonia or cognition.” | | Moderate  Moderate |

***Table I*** *continues on next page*

| ***Table I*** *continued* | | |  |  |  |
| --- | --- | --- | --- | --- | --- |
| **Author, year** | **Conclusions** | | | **Strength** |  |
| Mörkl *et al.* (2018)^44^ | *Abstract*: "﻿The use of nutritional interventions in psychiatry equips therapists with a promising tool for both the prevention and treatment of psychiatric disorders." [I]  *Discussion*: “Therefore, basic nutritional knowledge is essential for psychiatrists and psychotherapists. Lifestyle interventions such as dietary coaching could be used as promising, cost-effective and practical intervention for people with mental illness.” | | | Strong  Strong |  |
| Perez (2018)^45^ [II] | *Abstract*: “Epidemiological and observational evidence exists to support a protective effect of the Mediterranean diet in mood disorder prevalence and future diagnosis. To date, the majority of research has been conducted in adult populations. Future research is needed to examine if similar dietary relationships exist within a youth mood disorder population. Additionally, increased homogeneity in assessment methods of mood disorder symptoms and dietary patterns is needed; specifically, to determine more collective conclusions via meta-analyses.”  *Discussion*: "In summary, studies to date suggest the need for more rigorous large-scale randomized control trials to determine their benefit. Given the clinically significant negative side effects that are seen in the current psychotropic medications, as well as the unappealing risk:benefit ratio and unknown long-term effects, it is critical to explore alternative avenues for treatment. Public interest and demand for a nutrition- based treatment plan is rapidly increasing, in part encouraged by the growth of research supporting the feasibility and benefits of nutrition-based interventions in the child and adolescent mood disorder population. " | | | Moderate  Moderate |  |
| Hosker *et al.* (2019)^46^ | *Abstract*: "﻿Nutritional patterns that are high in a variety of fruits and vegetables, whole grains, seafood, and nuts, moderate in low-fat dairy products, low in red meat, and very limited in processed foods, saturated and trans fats, added sugars, and sodium have been associated with improved mental health outcomes across the lifespan" [I]  *Discussion*: “The benefits conferred by physical activity, balanced nutrition, and quality sleep to youth physical well-being have generally been accepted. Emerging evidence continues to shed light on their benefits for youth mental health and wellness as well, including psychiatric disorders.” | | | Strong  Strong |  |
| Huang *et al.* (2019)^47^ [II] | *Abstract*: “It is concluded that diet patterns, specific foods, and antioxidants play important roles in the prevention and clinical treatment of depression.”  *Discussion*: "Studies have shown that diet and nutrition play a significant role in the prevention and clinical treatment of depression" | | | Strong  Strong |  |
| Lopresti (2019)^48^ | *Abstract*: None  *Discussion*: "There is a strong body of evidence confirming inferior diet quality in people with depression. A poorer diet is also associated with an increased risk of developing future depression. However, a correlation does not confirm causation as poor eating could be an artefact of a depressed mood in many people. There is preliminary evidence to suggest dietary interventions (based on a Mediterranean diet) can effectively improve mood in depressed adults. However, this has mostly been conducted as an adjunctive treatment and its efficacy as a stand-alone intervention continues to be uncertain." [I] | | | -  Moderate |  |
| Kris-Etherton *et al.* (2020)^49^ [II] | ﻿*Abstract*: “Current evidence suggests that healthy eating patterns that meet food-based dietary recommendations and nutrient requirements may assist in the prevention and treatment of depression and anxiety.”  *Discussion*: “Current evidence does not support nutrient supplementation for the prevention of depressive disorders, but relatively convincing data from RCTs and prospective cohort studies suggest the Mediterranean diet and other healthy dietary patterns may assist in the prevention of depressive illnesses and potentially in the management of depression.” | | | Moderate  Moderate |  |
| Taylor *et al.* (2020)^50^ [II] | ﻿*Abstract*: “High-quality diets, prebiotics, and probiotics may beneficially affect mood. Habitual diets rich in dietary fiber and omega-3-polyunsaturated fatty acids may be linked to reduced risk of developing symptoms of depression, anxiety, and stress; however, additional studies are necessary.”  *Discussion*: “Reports from dietary interventions provide evidence that there is a link between diet quality and mood. Yet, the directionality of these relationships still needs to be discerned. To accomplish this, longitudinal studies and clinical trials need to be conducted and biological markers of disease need to be recorded.” | | | Moderate  Weak |  |
| *Abbreviations:* DASH, Dietary Approaches to Stop Hypertension; KD, ketogenic diet; n-3-PUFA, omega-3 polyunsaturated fatty acids. [I] Primary extracted conclusion; [II] Eligible papers after second search | | | | |  |
| **Table J.** Reported recommendations of included studies with classified strength (in chronological and alphabetical order) | | | | | |
| **Author, year** | | **Recommendations** | | **Strength** | |
| *Meta-analyses* | |  | |  | |
| Saghafian *et al.* (2018)^9^ | | *Abstract*: "This finding supports the current recommendation of increasing fruit and vegetable intake to improve mental health." [I]  *Discussion*: “The findings support the current recommendation of increasing fruit and vegetable intake to improve mental health” | | Strong  Strong | |
| Nicolaou *et al.* (2019)^12^ | | *Abstract*: None  *Discussion*: "﻿Promotion of a healthy diet, compatible with national dietary guidelines that are developed to prevent chronic diseases, may have additional benefits for mental health." [I] | | -  Moderate | |
| *Systematic reviews* | |  | |  | |
| Sanhueza *et al.* (2013)^17^ | | *Abstract*: "Strengthening healthy-eating patterns at the public health level may have a potential benefit." [I]  *Discussion*: “﻿However, we note that a diet rich in healthier foods and low in processed products coincides with the widely promoted healthy-eating pattern, which makes the case for strengthening these recommendations at the public health level.” | | Moderate  Moderate | |
| Opie *et al.* (2015)^19^ | | *Abstract*: None  *Discussion*: "﻿This is an important finding as it suggests that dietary interventions could potentially be used as a treatment and preventive approach at the clinical and population level." [I] | | -  Moderate | |
| Altun *et al.* (2019)^22^ | | *Abstract*: None  *Discussion*: "﻿The Mediterranean dietary pattern offers a potential alternative or useful adjunct to current treatments in place for depression, which procures few side effects, lessens disease severity and demonstrates a cost-effective measure that can be implemented globally." [I] | | -  Strong | |
| Tuck *et al.* (2019)^24^ [II] | | ﻿*Abstract*: None  *Discussion*: “Overall, based on the limited evidence to date, vegetable consumption is relevant to psychological health and could contribute to lifestyle medicine as an affordable preventative public health care strategy.” | | -  Moderate |  |
| Glabska *et al.* (2020)^25^ [II] | | ﻿*Abstract*: "Therefore, the general recommendation to consume at least 5 portions of fruit and vegetables a day may be beneficial also for mental health.”  *Discussion*: “Therefore, the general recommendation to consume at least 5 portions of fruit and vegetable a day may be beneficial also for mental health.” | | Moderate  Moderate |  |
| Ljungberg *et al.* (2020)^26^ [II] | | ﻿*Abstract*: “Public health professionals that work to support and motivate healthy eating habits may help prevent and treat depression based on the evidence presented in the results of this study.”  *Discussion*: “﻿A diet that protects and promotes depression should consist of vegetables, fruits, fibre, fish, whole grains, legumes and less added sugar, and processed foods. In the public health nurse’s preventative and health-promoting work, support and assistance with changing people’s dietary habits may be effective in promoting depression. [...] Resources should be used to help people maintain a healthy diet for preventive purposes for depression (strong evidence value).” | | Moderate  Strong |  |

| ***Table J*** *continues on next page* |
| --- |
| ***Table J*** *continued* |

| **Author, year** | **Recommendations** | | **Strength** | |
| --- | --- | --- | --- | --- |
| *Narrative reviews* |  | |  | |
| Bamber *et al.* (2007)^28^ [II] | | *Abstract*: “Dietary improvement and supplementation may offer an inexpensive and acceptable adjunct to standard treatment; yet this has, to date, been largely overlooked, owing to lack of evidence and knowledge.”  *Discussion*: "Dietary improvement and specific supplementation offer the potential for inexpensive, safe and acceptable intervention as an adjunct to standard treatment for depression in adolescence, as well as supporting wellbeing in healthy adolescents. | Moderate  Moderate |  |
| Low Dog (2010)^29^ | *Abstract*: None  *Discussion*: "When making dietary recommendations, clinicians should consider a low-glycemic, modified Mediterranean diet rich in fruits, vegetables, whole grains, and seafood (if not vegetarian) and low in processed, refined foods for optimizing mental health." [I] | | -  Strong | |
| Jacka & Berk (2012)^30^ | *Abstract*: "﻿Recommendations regarding dietary improvement, increases in physical activity and smoking cessation should be routinely given to patients with depression. Specialized and detailed advice may not be necessary. Recommendations should focus on following national guidelines for healthy eating and physical activity." [I]  *Discussion*: “Recommendations and encouragement to follow national guidelines for dietary and exercise practices should be a part of care for all people with depression. This is particularly so for patients with difficult-to-treat depression that has not responded to standard elements of care.” | | Strong  Strong | |
| Jacka *et al.* (2012)^31^ | *Abstract*: "We believe that it is both feasible and timely to begin to develop effective, sustainable, population-level prevention initiatives for the common mental illnesses that build on the established and developing approaches to the noncommunicable somatic diseases." [I]  *Discussion*: “…and call for the development of a coordinated strategy for the universal primary prevention of the common mental disorders that builds on the established and developing approaches to the noncommunicable somatic diseases.” | | Strong  Strong | |
| Berk *et al.* (2013)^32^ | *Abstract*: "﻿While there is yet little trial evidence to support smoking cessation or dietary advice in depression management, the precautionary principle would support both." [I]  *Discussion*: “Lifestyle modification, with a focus on exercise, enhancing social networks, exposure to green space, diet and smoking, is of substantial potential value, not only to the high prevalence psychiatric disorders, but to those medical disorders that are over-represented in these individuals.” | | Moderate  Strong | |
| Sarris *et al.* (2014)^35^ | *Abstract*: “While the judicious use of medication and psychological techniques are still advocated, due to the complexity of human illness/wellbeing, the emerging evidence encourages a more integrative approach for depression, and an acknowledgment that lifestyle modification should be a routine part of treatment and preventative efforts.”  *Discussion/main text*: "Regardless of the current deficit of empirical evidence for prescriptive dietary advice to treat depression, it is recognized that diet has a major impact on comorbid medical conditions that are exceedingly more common in people diagnosed with depression, including cardiovascular disease and metabolic disorders, and the precautionary principle should guide practice." [I] | | Strong  Strong | |
| LaChance & Ramsey (2015)^36^ | *Abstract*: None  *Discussion*: “﻿In order to promote mental health and recovery from mental illness, one could consider encouraging patients to eat a diet that is optimal for brain health." [I] | | -  Moderate | |

| ***Table J*** *continues on next page*   \| ***Table J*** *continued* \| \| --- \| |
| --- | --- |

| **Author, year** | **Recommendations** | | **Strength** | |
| --- | --- | --- | --- | --- |
| Lang *et al.* (2015)^37^ | *Abstract*: "﻿In this context, dietary and lifestyle interventions may be a desirable, effective, pragmatical and non-stigmatizing prevention and treatment strategy for depression." [I]  *Discussion*: None | | Moderate  - | |
| Sarris *et al.* (2015)^38^ | *Abstract*: None  *Discussion*: "﻿Nutritional medicine should now be considered as a mainstream element of psychiatric practice, with research, education, policy, and health promotion supporting this new framework." [I] | | -  Strong | |
| Jacka (2017)^39^ | *Abstract*: "Key challenges for the field are; […]; and continue to advocate for policy change to improve the food environment at the population level." [I]  *Discussion*: “However, given the very large burden of illness imposed by mental disorders and the evidence supporting the importance of nutrition to mental and brain health, it will be critical to continue to liaise with key allies in public health to advocate for policy change to improve the food environment at the population level.” | | Strong  Strong | |
| Opie *et al.* (2017)^41^ | *Abstract*: "Five key dietary recommendations for the prevention of depression emerged from current published evidence. These comprise: (1) follow ‘traditional’ dietary patterns, such as the Mediterranean, Norwegian, or Japanese diet; (2) increase consumption of fruits, vegetables, legumes, wholegrain cereals, nuts, and seeds; (3) include a high consumption of foods rich in omega-3 polyunsaturated fatty acids; (4) replace unhealthy foods with wholesome nutritious foods; (5) limit your intake of processed-foods, ‘fast’ foods, commercial bakery goods, and sweets." [I]  *Results*: Same as abstract | | Strong    Strong | |
| Brietzke *et al.* (2018)^43^ | *Abstract*: "Because of its potential pleiotropic benefits, ketogenic diet should be considered as a promising intervention in research in mood disorder therapeutics, especially in treatment resistant presentations." [I]  *Discussion*: “The “metabolic therapy” of mood disorders should necessarily explore KD using rigorous approaches as one of the most promising interventions.” | | Strong  Strong | |
| Mörkl *et al.* (2018)^44^ | *Abstract*: "Besides pharmacological therapy, psychotherapy and physical activity, nutritional interventions are an important pillar in the multifactorial, biopsychosocial treatment of psychiatric disease and could be used as a potential therapeutic target." [I]  *Discussion*: “Nutritional interventions should be integrated as an important pillar in the multifactorial, biopsychosocial treatment of our patients.” | | Strong  Strong | |
| Perez (2018)^45^ [II] | | *Abstract*: “Results from youth studies could be used to formulate future randomized controlled trials, health promotion programs or clinical interventions, via diet or supplement interventions, for alternative mood disorder treatment or prevention purposes.”  *Discussion*: "Future research should explore plans for developing and implementing a personalized nutrition treatment approach for the mood disorder patient from either a diet and nutrient supplementation perspective, as well as its outcomes, for comparison to current standard of care." | Moderate  Strong |  |
| Hosker *et al.* (2019)^46^ | *Abstract*: None  *Discussion*: “Integrating ongoing assessments and interventions related to these lifestyle domains within clinical practice promotes positive mental as well as physical health in youth, including enhancing treatment of psychiatric disorders and their impacts on functioning.” | | -  Strong |  |

| ***Table J*** *continues on next page*   \| ***Table J*** *continued* \| \| --- \| |
| --- | --- |

| **Author, year** | **Recommendations** | **Strength** | |
| --- | --- | --- | --- |
| Huang *et al.* (2019)^47^ [II] | *Abstract*: None  *Discussion*: "Diet and nutrition can be used as a part of a comprehensive strategy for the prevention of depressive problems. Moreover, patients with depression who are not suitable for drug therapy or psychotherapy can use diet and nutrition adjustments as an alternative treatment." | -  Strong |  |
| Kris-Etherton *et al.* (2020)^49^ [II] | ﻿*Abstract*: None  *Discussion*: “Adoption of a healthy eating pattern that meets food-based dietary recommendations and nutrient requirements is important to prevent, slow the progression of, or manage depressive symptoms, as well as promote optimal mental health. | -  Strong |  |
| *Abbreviations:* AD, Alzheimer’s disease; [I] Primary extracted recommendation | | | |

References

1 Psaltopoulou T, Sergentanis TN, Panagiotakos DB, Sergentanis IN, Kosti R, Scarmeas N. Mediterranean diet, stroke, cognitive impairment, and depression: A meta-analysis. *Ann Neurol* 2013; **74**: 580–591.

2 Lai JS, Hiles S, Bisquera A, Hure AJ, McEvoy M, Attia J. A systematic review and meta-analysis of dietary patterns and depression in community-dwelling adults. *Am J Clin Nutr* 2014; **99**: 181–97.

3 Rahe C, Unrath M, Berger K. Dietary patterns and the risk of depression in adults: A systematic review of observational studies. *Eur J Nutr* 2014; **53**: 997–1013.

4 Li F, Liu X, Zhang D. Fish consumption and risk of depression: A meta-analysis. *J Epidemiol Community Health* 2015; **70**: 299–304.

5 Grosso G, Micek A, Marventano S, Castellano S, Mistretta A, Pajak A *et al.* Dietary n-3 PUFA, fish consumption and depression: A systematic review and meta-analysis of observational studies. *J Affect Disord* 2016; **205**: 269–281.

6 Liu X, Yan Y, Li F, Zhang D. Fruit and vegetable consumption and the risk of depression: A meta-analysis. *Nutrition* 2016; **32**: 296–302.

7 Li Y, Lv M-R, Wei Y-J, Sun L, Zhang J-X, Zhang H-G *et al.* Dietary patterns and depression risk: A meta-analysis. *Psychiatry Res* 2017; **253**: 373–382.

8 Molendijk M, Molero P, Ortuño Sánchez-Pedreño F, Van der Does W, Angel Martínez-González M. Diet quality and depression risk: A systematic review and dose-response meta-analysis of prospective studies. *J Affect Disord* 2018; **226**: 346–354.

9 Saghafian F, Malmir H, Saneei P, Milajerdi A, Larijani B, Esmaillzadeh A. Fruit and vegetable consumption and risk of depression: Accumulative evidence from an updated systematic review and meta-Analysis of epidemiological studies. *Br J Nutr* 2018; **119**: 1087–1101.

10 Yang Y, Kim Y, Je Y. Fish consumption and risk of depression: Epidemiological evidence from prospective studies. *Asia-Pacific Psychiatry* 2018; **10**: e12335.

11 Lassale C, Batty GD, Baghdadli A, Jacka F, Sánchez-Villegas A, Kivimäki M *et al.* Healthy dietary indices and risk of depressive outcomes: a systematic review and meta-analysis of observational studies. *Mol Psychiatry* 2019; **24**: 965–986.

12 Nicolaou M, Colpo M, Vermeulen E. Association of a priori dietary patterns with depressive symptoms: a harmonized meta-analysis of observational studies. *Psychol Med* 2019.in press.

13 Salari-Moghaddam A, Saneei P, Larijani B, Esmaillzadeh A. Glycemic index, glycemic load, and depression: a systematic review and meta-analysis. *Eur J Clin Nutr* 2019; **73**: 356–365.

14 Shafiei F, Salari-Moghaddam A, Larijani B, Esmaillzadeh A. Adherence to the mediterranean diet and risk of depression: A systematic review and updated meta-analysis of observational studies. *Nutr Rev* 2019; **77**: 230–239.

15 Murakami K, Sasaki S. Dietary intake and depressive symptoms: A systematic review of observational studies. *Mol Nutr Food Res* 2010; **54**: 471–488.

16 Quirk SE, Williams LJ, O’Neil A, Pasco JA, Jacka FN, Housden S *et al.* The association between diet quality, dietary patterns and depression in adults: a systematic review. *BMC Psychiatry* 2013; **13**: 175.

17 Sanhueza C, Ryan L, Foxcroft DR. Diet and the risk of unipolar depression in adults: Systematic review of cohort studies. *J Hum Nutr Diet* 2013; **26**: 56–70.

18 O’Neil A, Quirk SE, Housden S, Brennan SL, Williams LJ, Pasco JA *et al.* Relationship between diet and mental health in children and adolescents: A systematic review. *Am J Public Health* 2014; **104**: e31–e42.

19 Opie RS, O’Neil A, Itsiopoulos C, Jacka FN. The impact of whole-of-diet interventions on depression and anxiety: A systematic review of randomised controlled trials. *Public Health Nutr* 2015; **18**: 2074–2093.

20 Khalid S, Williams CM, Reynolds SA. Is there an association between diet and depression in children and adolescents? A systematic review. *Br J Nutr* 2016; **116**: 2097–2108.

21 Rahimlou M, Morshedzadeh N, Karimi S, Jafarirad S. Association between dietary glycemic index and glycemic load with depression: a systematic review. *Eur J Nutr* 2018; **57**: 2333–2340.

22 Altun A, Brown H, Szoeke C, Goodwill AM. The Mediterranean dietary pattern and depression risk: A systematic review. *Neurol Psychiatry Brain Res* 2019; **33**: 1–10.

23 Arab A, Mehrabani S, Moradi S, Amani R. The association between diet and mood: A systematic review of current literature. *Psychiatry Res* 2019; **271**: 428–437.

24 Tuck N-J, Farrow C, Thomas JM. Assessing the effects of vegetable consumption on the psychological health of healthy adults: a systematic review of prospective research. *Am J Clin Nutr* 2019; **110**: 196–211.

25 Głąbska D, Guzek D, Groele B, Gutkowska K. Fruit and Vegetable Intake and Mental Health in Adults: A Systematic Review. *Nutrients* 2020; **12**: 115.

26 Ljungberg T, Bondza E, Lethin C. Evidence of the Importance of Dietary Habits Regarding Depressive Symptoms and Depression. *Int J Environ Res Public Health* 2020; **17**: 1616.

27 McGrath-Hanna NK, Greene DM, Tavernier RJ, Bult-Ito A. Diet and mental health in the Arctic: is diet an important risk factor for mental health in circumpolar peoples? - a review. *Int J Circumpolar Health* 2003; **62**: 228–241.

28 Bamber DJ, Stokes CS, Stephen AM. The role of diet in the prevention and management of adolescent depression. *Nutr Bull* 2007; **32**: 90–99.

29 Low Dog T. The role of nutrition in mental health. *Altern Ther Health Med* 2010; **16**: 42–46.

30 Jacka FN, Berk M. Depression, diet and exercise. *Med J Aust* 2012; **199**: S21–S23.

31 Jacka FN, Mykletun A, Berk M. Moving towards a population health approach to the primary prevention of common mental disorders. *BMC Med* 2012; **10**: 149.

32 Berk M, Sarris J, Coulson CE, Jacka FN. Lifestyle management of unipolar depression. *Acta Psychiatr Scand* 2013; **127**: 38–54.

33 Manosso LM, Moretti M, Rodrigues ALS. Nutritional strategies for dealing with depression. *Food Funct* 2013; **4**: 1776–1793.

34 Sanchez-Villegas A, Martínez-González MA. Diet, a new target to prevent depression? *BMC Med* 2013; **11**: 3.

35 Sarris J, O’Neil A, Coulson CE, Schweitzer I, Berk M. Lifestyle medicine for depression. *BMC Psychiatry* 2014; **14**: 1–13.

36 Lachance L, Ramsey D. Food, mood, and brain health: implications for the modern clinician. *Mo Med* 2015; **112**: 111–115.

37 Lang UE, Beglinger C, Schweinfurth N, Walter M, Borgwardt S. Nutritional aspects of depression. *Cell Physiol Biochem* 2015; **37**: 1029–1043.

38 Sarris J, Logan AC, Akbaraly TN, Amminger GP, Balanzá-Martínez V, Freeman MP *et al.* Nutritional medicine as mainstream in psychiatry. *The Lancet Psychiatry* 2015; **2**: 271–274.

39 Jacka FN. Nutritional Psychiatry: Where to Next? *EBioMedicine* 2017; **17**: 24–29.

40 Libuda L, Antel J, Hebebrand J, Föcker M. Nutrition and mental diseases: Focus depressive disorders. *Nervenarzt* 2017; **88**: 87–101.

41 Opie RS, Itsiopoulos C, Parletta N, Sanchez-Villegas A, Akbaraly TN, Ruusunen A *et al.* Dietary recommendations for the prevention of depression. *Nutr Neurosci* 2017; **20**: 161–171.

42 Stevenson RJ. Psychological correlates of habitual diet in healthy adults. *Psychol Bull* 2017; **143**: 53–90.

43 Brietzke E, Mansur RB, Subramaniapillai M, Banlanzá-Martínez V, Vinberg M, González-Pinto A *et al.* Ketogenic diet as a metabolic therapy for mood disorders: Evidence and developments. *Neurosci Biobehav Rev* 2018; **94**: 11–16.

44 Mörkl S, Wagner-Skacel J, Lahousen T, Lackner S, Holasek SJ, Bengesser SA *et al.* The Role of Nutrition and the Gut-Brain Axis in Psychiatry: A Review of the Literature. *Neuropsychobiology* 2018; : 1–9.

45 Perez L. The Role of Dietary Patterns in Mood Disorders: Prospective Research in Youth Populations. *Am J Lifestyle Med* 2018; **12**: 286–290.

46 Hosker DK, Elkins RM, Potter MP. Promoting Mental Health and Wellness in Youth Through Physical Activity, Nutrition, and Sleep. *Child Adolesc Psychiatr Clin N Am* 2019; **28**: 171–193.

47 Huang Q, Liu H, Suzuki K, Ma S, Liu C. Linking What We Eat to Our Mood: A Review of Diet, Dietary Antioxidants, and Depression. *Antioxidants* 2019; **8**: 376.

48 Lopresti AL. It is time to investigate integrative approaches to enhance treatment outcomes for depression? *Med Hypotheses* 2019; **126**: 82–94.

49 Kris-Etherton PM, Petersen KS, Hibbeln JR, Hurley D, Kolick V, Peoples S *et al.* Nutrition and behavioral health disorders: depression and anxiety. *Nutr Rev* 2020; **00**: 1–14.

50 Taylor AM, Holscher HD. A review of dietary and microbial connections to depression, anxiety, and stress. *Nutr Neurosci* 2020; **23**: 237–250.
