# Supplementary material for: Impact of review method on the conclusions of clinical reviews: A systematic review on dietary interventions in depression as a case in point
Source: PLoS One. 2020 Sep 16;15(9):e0238131. doi: 10.1371/journal.pone.0238131 (PMC7494108; doi:10.1371/journal.pone.0238131)
Supplement: S4 Table — (DOCX) [file pone.0238131.s007.docx]

| **Table L.** AMSTAR II scoring table | | | | | | | | | | | | | | | | | |
| --- | --- | --- | --- | --- | --- | --- | --- | --- | --- | --- | --- | --- | --- | --- | --- | --- | --- |
| **Author, year** | **I1** | **I2** | **I3** | **I4** | **I5** | **I6** | **I7** | **I8** | **I9** | **I10** | **I11** | **I12** | **I13** | **I14** | **I15** | **I16** | **AMSTAR score** |
| *Meta-analyses* |  |  |  |  |  |  |  |  |  |  |  |  |  |  |  |  |  |
| Psaltopoulou *et al.* (2013)^1^ | Y | N | N | N | N | Y | N | Y | P | N | Y | N | Y | Y | Y | Y | Critically low |
| Lai *et al.* (2014)^2^ | N | N | Y | N | Y | Y | N | Y | P | N | Y | Y | Y | Y | Y | Y | Critically low |
| Rahe *et al.* (2014)^3^ | N | N | N | N | N | N | N | Y | P | N | N/A | N/A | Y | Y | N/A | Y | Critically low |
| Li *et al.* (2015)^4^ | Y | N | Y | N | Y | Y | N | N | P | N | Y | N | Y | Y | Y | Y | Critically low |
| Grosso *et al.* (2016)^5^ | N | N | Y | N | N | Y | N | Y | P | N | Y | Y | Y | Y | Y | Y | Critically low |
| Liu *et al.* (2016)^6^ | N | N | N | P | Y | N | N | P | N | N | Y | N | N | Y | N | N | Critically low |
| Li *et al.* (2017)^7^ | N | N | Y | N | Y | N | N | N | P | N | Y | N | Y | Y | Y | N | Critically low |
| Molendijk *et al.* (2018)^8^ | Y | Y | Y | P | P | P | N | Y | P | N | Y | Y | Y | Y | Y | Y | Low |
| Saghafian *et al.* (2018)^9^ | Y | N | Y | P | N | N | N | Y | P | N | Y | Y | N | Y | Y | Y | Critically low |
| Yang *et al.* (2018)^10^ | Y | N | Y | N | N | Y | Y | Y | P | N | Y | Y | Y | Y | Y | Y | Critically low |
| Lassale *et al.* (2019)^11^ | Y | P | N | N | Y | Y | Y | Y | P | N | Y | N | Y | Y | N | Y | Critically low |
| Nicolaou *et al.* (2019)^12^ | Y | N | N | N | N | N | N | P | N | N | Y | N | N | Y | N | N | Critically low |
| Salari-Moghaddam *et al.* (2019)^13^ | Y | P | Y | P | N | N | Y | P | P | N | Y | Y | Y | Y | Y | Y | Moderate |
| Shafiei *et al.* (2019)^14^ | Y | N | Y | P | N | N | Y | Y | P | N | Y | Y | Y | Y | Y | Y | Low |
| *Systematic reviews* |  |  |  |  |  |  |  |  |  |  |  |  |  |  |  |  |  |
| Murakami *et al.* (2010)^15^ | N | N | Y | N | N | N | N | Y | N | N | N/A | N/A | Y | Y | N/A | Y | Critically low |
| Quirk *et al.* (2013)^16^ | Y | N | Y | N | N | N | N | P | P | N | N/A | N/A | Y | Y | N/A | N | Critically low |
| Sanhueza *et al.* (2013)^17^ | Y | N | Y | N | N | N | N | Y | Y | Y | N/A | N/A | Y | Y | N/A | Y | Critically low |
| O'Neil *et al.* (2014)^18^ | Y | N | Y | N | Y | N | Y | Y | P | N | N/A | N/A | Y | Y | N/A | N | Critically low |
| Opie *et al.* (2015)^19^ | Y | N | Y | N | N | Y | N | Y | P | N | N/A | N/A | N | Y | N/A | Y | Critically low |
| Khalid *et al.* (2017)^20^ | Y | N | Y | N | N | N | N | Y | Y | N | N/A | N/A | Y | Y | N/A | Y | Critically low |
| Rahimlou *et al.* (2018)^21^ | Y | N | N | N | N | N | N | Y | P | N | N/A | N/A | Y | Y | N/A | Y | Critically low |
| Altun *et al.* (2019)^22^ | Y | N | N | N | Y | N | N | P | P | N | N/A | N/A | Y | Y | N/A | Y | Critically low |
| Arab *et al.* (2019)^23^ | Y | N | Y | N | N | N | N | Y | P | N | N/A | N/A | Y | Y | N/A | Y | Critically low |
| Tuck *et al.* (2019)^24^ | Y | N | N | N | N | Y | Y | Y | N | N | N/A | N/A | N | N | N/A | Y | Critically low |
| Glabska *et al.* (2020)^25^ | Y | Y | N | N | Y | Y | N | Y | P | N | N/A | N/A | N | N | N/A | Y | Critically low |
| Ljungberg *et al.* (2020)^26^ | Y | N | Y | N | Y | N | N | P | N | N | N/A | N/A | Y | N | N/A | Y | Critically low |
| *Note.* Critical items are highlighted in grey. *Abbreviations:* I, item number; N, no; N/A, not applicable; P, partial; Y, yes. *Items:* I1 = Did the research questions and inclusion criteria for the review include the components of PICO? ﻿I2 = Did the report of the review contain an explicit statement that the review methods were established prior to the conduct of the review and did the report justify any significant deviations from the protocol? ﻿I3 = Did the review authors explain their selection of the study designs for inclusion in the review? ﻿I4 = Did the review authors use a comprehensive literature search strategy? ﻿I5 = Did the review authors perform study selection in duplicate? ﻿I6 = Did the review authors perform data extraction in duplicate? ﻿I7 = Did the review authors provide a list of excluded studies and justify the exclusions? ﻿I8 = Did the review authors describe the included studies in adequate detail? ﻿I9 = Did the review authors use a satisfactory technique for assessing the risk of bias (RoB) in individual studies that were included in the review? ﻿I10 = Did the review authors report on the sources of funding for the studies included in the review? ﻿I11 = If meta-analysis was performed did the review authors use appropriate methods for statistical combination of results? ﻿I12 = If meta-analysis was performed, did the review authors assess the potential impact of RoB in individual studies on the results of the meta-analysis or other evidence synthesis? ﻿I13 = Did the review authors account for RoB in individual studies when interpreting/ discussing the results of the review? ﻿I14 = Did the review authors provide a satisfactory explanation for, and discussion of, any heterogeneity observed in the results of the review? ﻿I15 = If they performed quantitative synthesis did the review authors carry out an adequate investigation of publication bias (small study bias) and discuss its likely impact on the results of the review? ﻿I16 = Did the review authors report any potential sources of conflict of interest, including any funding they received for conducting the review? | | | | | | | | | | | | | | | | | |

References

1 Psaltopoulou T, Sergentanis TN, Panagiotakos DB, Sergentanis IN, Kosti R, Scarmeas N. Mediterranean diet, stroke, cognitive impairment, and depression: A meta-analysis. *Ann Neurol* 2013; **74**: 580–591.

2 Lai JS, Hiles S, Bisquera A, Hure AJ, McEvoy M, Attia J. A systematic review and meta-analysis of dietary patterns and depression in community-dwelling adults. *Am J Clin Nutr* 2014; **99**: 181–97.

3 Rahe C, Unrath M, Berger K. Dietary patterns and the risk of depression in adults: A systematic review of observational studies. *Eur J Nutr* 2014; **53**: 997–1013.

4 Li F, Liu X, Zhang D. Fish consumption and risk of depression: A meta-analysis. *J Epidemiol Community Health* 2015; **70**: 299–304.

5 Grosso G, Micek A, Marventano S, Castellano S, Mistretta A, Pajak A *et al.* Dietary n-3 PUFA, fish consumption and depression: A systematic review and meta-analysis of observational studies. *J Affect Disord* 2016; **205**: 269–281.

6 Liu X, Yan Y, Li F, Zhang D. Fruit and vegetable consumption and the risk of depression: A meta-analysis. *Nutrition* 2016; **32**: 296–302.

7 Li Y, Lv M-R, Wei Y-J, Sun L, Zhang J-X, Zhang H-G *et al.* Dietary patterns and depression risk: A meta-analysis. *Psychiatry Res* 2017; **253**: 373–382.

8 Molendijk M, Molero P, Ortuño Sánchez-Pedreño F, Van der Does W, Angel Martínez-González M. Diet quality and depression risk: A systematic review and dose-response meta-analysis of prospective studies. *J Affect Disord* 2018; **226**: 346–354.

9 Saghafian F, Malmir H, Saneei P, Milajerdi A, Larijani B, Esmaillzadeh A. Fruit and vegetable consumption and risk of depression: Accumulative evidence from an updated systematic review and meta-Analysis of epidemiological studies. *Br J Nutr* 2018; **119**: 1087–1101.

10 Yang Y, Kim Y, Je Y. Fish consumption and risk of depression: Epidemiological evidence from prospective studies. *Asia-Pacific Psychiatry* 2018; **10**: e12335.

11 Lassale C, Batty GD, Baghdadli A, Jacka F, Sánchez-Villegas A, Kivimäki M *et al.* Healthy dietary indices and risk of depressive outcomes: a systematic review and meta-analysis of observational studies. *Mol Psychiatry* 2019; **24**: 965–986.

12 Nicolaou M, Colpo M, Vermeulen E. Association of a priori dietary patterns with depressive symptoms: a harmonized meta-analysis of observational studies. *Psychol Med* 2019.in press.

13 Salari-Moghaddam A, Saneei P, Larijani B, Esmaillzadeh A. Glycemic index, glycemic load, and depression: a systematic review and meta-analysis. *Eur J Clin Nutr* 2019; **73**: 356–365.

14 Shafiei F, Salari-Moghaddam A, Larijani B, Esmaillzadeh A. Adherence to the mediterranean diet and risk of depression: A systematic review and updated meta-analysis of observational studies. *Nutr Rev* 2019; **77**: 230–239.

15 Murakami K, Sasaki S. Dietary intake and depressive symptoms: A systematic review of observational studies. *Mol Nutr Food Res* 2010; **54**: 471–488.

16 Quirk SE, Williams LJ, O’Neil A, Pasco JA, Jacka FN, Housden S *et al.* The association between diet quality, dietary patterns and depression in adults: a systematic review. *BMC Psychiatry* 2013; **13**: 175.

17 Sanhueza C, Ryan L, Foxcroft DR. Diet and the risk of unipolar depression in adults: Systematic review of cohort studies. *J Hum Nutr Diet* 2013; **26**: 56–70.

18 O’Neil A, Quirk SE, Housden S, Brennan SL, Williams LJ, Pasco JA *et al.* Relationship between diet and mental health in children and adolescents: A systematic review. *Am J Public Health* 2014; **104**: e31–e42.

19 Opie RS, O’Neil A, Itsiopoulos C, Jacka FN. The impact of whole-of-diet interventions on depression and anxiety: A systematic review of randomised controlled trials. *Public Health Nutr* 2015; **18**: 2074–2093.

20 Khalid S, Williams CM, Reynolds SA. Is there an association between diet and depression in children and adolescents? A systematic review. *Br J Nutr* 2016; **116**: 2097–2108.

21 Rahimlou M, Morshedzadeh N, Karimi S, Jafarirad S. Association between dietary glycemic index and glycemic load with depression: a systematic review. *Eur J Nutr* 2018; **57**: 2333–2340.

22 Altun A, Brown H, Szoeke C, Goodwill AM. The Mediterranean dietary pattern and depression risk: A systematic review. *Neurol Psychiatry Brain Res* 2019; **33**: 1–10.

23 Arab A, Mehrabani S, Moradi S, Amani R. The association between diet and mood: A systematic review of current literature. *Psychiatry Res* 2019; **271**: 428–437.

24 Tuck N-J, Farrow C, Thomas JM. Assessing the effects of vegetable consumption on the psychological health of healthy adults: a systematic review of prospective research. *Am J Clin Nutr* 2019; **110**: 196–211.

25 Głąbska D, Guzek D, Groele B, Gutkowska K. Fruit and Vegetable Intake and Mental Health in Adults: A Systematic Review. *Nutrients* 2020; **12**: 115.

26 Ljungberg T, Bondza E, Lethin C. Evidence of the Importance of Dietary Habits Regarding Depressive Symptoms and Depression. *Int J Environ Res Public Health* 2020; **17**: 1616.
