# Supplementary material for: Impact of review method on the conclusions of clinical reviews: A systematic review on dietary interventions in depression as a case in point
Source: PLoS One. 2020 Sep 16;15(9):e0238131. doi: 10.1371/journal.pone.0238131 (PMC7494108; doi:10.1371/journal.pone.0238131)
Supplement: S5 Table — (DOCX) [file pone.0238131.s008.docx]

| **Table M.** Year of publication, strength of conclusion, number of input papers, various indicators of impact, and potential conflicts of interest by study type and study | | | | | | | | | |
| --- | --- | --- | --- | --- | --- | --- | --- | --- | --- |
| **Author** | **Year** | **Conclusion: Abstract** | **Conclusion: Discussion** | **# input papers ^a^** | **# citations ^b^** | **Altmetric ^c^** | **IF ^c^** | **PFCOI** | **ISNPR** |
| *Meta-analyses* |  |  |  |  |  |  |  |  |  |
| Psaltopoulou *et al.*^1^ | 2013 | Moderate (2) | Moderate (2) | 9 | 522 | 654 | 10.24 | No | No |
| Lai *et al.*^2^ | 2014 | Weak (1) | Moderate (2) | 21 | 468 | 369 | 7.77 | No | No |
| Rahe *et al.*^3^ | 2014 | Weak (1) | Weak (1) | 16 | 133 | 130 | 4.45 | No | No |
| Li *et al.*^4^ | 2015 | Moderate (2) | Moderate (2) | 26 | 102 | 692 | 3.87 | No | No |
| Grosso *et al.*^5^ | 2016 | Moderate (2) | Moderate (2) | 31 | 89 | 124 | 4.08 | No | No |
| Liu *et al.*^6^ | 2016 | Weak (1) | Weak (1) | 10 | 131 | 42 | 3.59 | N/K | No |
| Li *et al.*^7^ | 2017 | Weak (1) | Moderate (2) | 21 | 138 | 810 | 2.68 | No | No |
| Molendijk *et al.*^8^ | 2018 | Weak (1) | Weak (1) | 24 | 132 | 182 | 4.08 | No | No |
| Saghafian *et al.*^9^ | 2018 | Moderate (2) | Moderate (2) | 27 | 34 | 121 | 3.66 | No | No |
| Yang *et al.* ^10^ | 2018 | Moderate (2) | Moderate (2) | 10 | 16 | 2 | 1.14 | No | No |
| Lassale *et al.*^11^ | 2019 | Moderate (2) | Moderate (2) | 41 | 104 | 1632 | 11.97 | No | Yes |
| Nicolaou *et al.*^12^ | 2019 | Moderate (2) | Moderate (2) | 6 | 4 | 34 | 5.64 | No | No |
| Salari-Moghaddam *et al.*^13^ | 2019 | Weak (1) | Weak (1) | 11 | 10 | 19 | 3.06 | No | No |
| Shafiei *et al.*^14^ | 2019 | Weak (1) | Weak (1) | 14 | 12 | 13 | 5.79 | No | No |
| Average (SD) or *n* (%) | 2017 (2) | 1.50 (0.52) | 1.64 (0.50) | 19 (10) | 135 (161) | 345 (463) | 5.14 (2.98) | No (93%) | No (93%) |
| *Systematic reviews* |  |  |  |  |  |  |  |  |  |
| Murakami *et al.*^15^ | 2010 | Weak (1) | Weak (1) | 34 | 133 | 1 | 4.91 | No | No |
| Quirk *et al.*^16^ | 2013 | Moderate (2) | Moderate (2) | 25 | 221 | 60 | 2.67 | Yes | Yes |
| Sanhueza *et al.*^17^ | 2013 | Weak (1) | Weak (1) | 11 | 127 | 20 | 2.68 | No | No |
| O'Neil *et al.*^18^ | 2014 | Moderate (2) | Moderate (2) | 12 | 311 | 414 | 4.14 | Yes | Yes |
| Opie *et al.*^19^ | 2015 | Moderate (2) | Moderate (2) | 17 | 129 | 25 | 2.48 | Yes | Yes |
| Khalid *et al.*^20^ | 2017 | Weak (1) | Weak (1) | 20 | 69 | 11 | 3.30 | No | No |
| Rahimlou *et al.*^21^ | 2018 | Moderate (2) | Moderate (2) | 6 | 3 | 4 | 4.45 | No | No |
| Altun *et al.*^22^ | 2019 | Strong (3) | Strong (3) | 26 | 3 | 9 | 0.41 | No | No |
| Arab *et al.*^23^ | 2019 | Moderate (2) | Moderate (2) | 18 | 5 | 25 | 2.68 | No | No |
| Tuck *et al.*^24^ | 2019 | Moderate (2) | Moderate (2) | 10 | 3 | 26 | 6.77 | No | No |
| Glabska *et al.*^25^ | 2020 | Moderate (2) | Moderate (2) | 61 | 3 | 41 | 4.17 | Yes | No |
| Ljungberg *et al.*^26^ | 2020 | Moderate (2) | Moderate (2) | 22 | 0 | 5 | 2.47 | No | No |
| Average (SD) | 2016 (3) | 1.83 (0.58) | 1.83 (0.58) | 22 (15) | 84 (103) | 53 (115) | 3.43 (1.60) | No (67%) | No (75%) |
| ***Table M*** *continues on next page* | | | | | | | | | |
| ***Table M*** *continued* | | | | | | | | | |
| **Author** | **Year** | **Conclusion: Abstract** | **Conclusion: Discussion** | **# input papers ^a^** | **# citations ^b^** | **Altmetric ^c^** | **IF ^d^** | **PFCOI** | **ISNPR** |
| *Narrative reviews* |  |  |  |  |  |  |  |  |  |
| McGrath-Hanna *et al.*^27^ | 2003 | Moderate (2) | Moderate (2) | 2 | 116 | 32 | 1.06 | No | No |
| Bamber *et al.*^28^ | 2007 | Moderate (2) | Moderate (2) | 1 | 41 | 3 | 1.88 | N/K | No |
| Low Dog^29^ | 2010 | N/A | Moderate (2) | 3 | 28 | N/K | 1.33 | N/K | No |
| Jacka & Berk^30^ | 2012 | Strong (3) | Strong (3) | 10 | 47 | 8 | 4.23 | No | Yes |
| Jacka *et al.*^31^ | 2012 | Strong (3) | Strong (3) | 17 | 138 | 167 | 9.09 | No | Yes |
| Berk *et al.*^32^ | 2013 | Strong (3) | Strong (3) | 18 | 90 | 6 | 5.61 | Yes | Yes |
| Manosso *et al.*^33^ | 2013 | N/A | Moderate (2) | 3 | 28 | 10 | 3.28 | N/K | No |
| Sanchez-Villegas *et al.*^34^ | 2013 | Weak (1) | Weak (1) | 10 | 126 | 51 | 9.09 | No | Yes |
| Sarris *et al.*^35^ | 2014 | Strong (3) | Moderate (2) | 14 | 167 | 66 | 3.30 | No | Yes |
| LaChance & Ramsey^36^ | 2015 | Moderate (2) | Moderate (2) | 6 | 9 | 32 | 0.16 | No | Yes |
| Lang *et al.*^37^ | 2015 | Weak (1) | Weak (1) | 10 | 120 | 112 | 5.50 | No | No |
| Sarris *et al.*^38^ | 2015 | Strong (3) | Strong (3) | 5 | 288 | 985 | 18.33 | Yes | Yes |
| Jacka^39^ | 2017 | Moderate (2) | Moderate (2) | 18 | 79 | 256 | 6.68 | Yes | Yes |
| Libuda *et al.*^40^ | 2017 | Moderate (2) | Weak (1) | 5 | 10 | 1 | 0.83 | No | No |
| Opie *et al.*^41^ | 2017 | Moderate (2) | Moderate (2) | 17 | 104 | 260 | 3.77 | Yes | Yes |
| Stevenson^42^ | 2017 | Moderate (2) | Weak (1) | 13 | 26 | 1 | 13.25 | N/K | No |
| Brietzke *et al.*^43^ | 2018 | Moderate (2) | Moderate (2) | 2 | 24 | 162 | 9.44 | N/K | Yes |
| Mörkl *et al.*^44^ | 2018 | Strong (3) | Strong (3) | 9 | 24 | 92 | 1.80 | No | No |
| Perez^45^ | 2018 | Moderate (2) | Moderate (2) | 24 | 0 | 0 | 2.00 | No | No |
| Hosker *et al.*^46^ | 2019 | Strong (3) | Strong (3) | 10 | 6 | 0 | 1.64 | N/K | No |
| Huang *et al.*^47^ | 2020 | Strong (3) | Strong (3) | 24 | 6 | 31 | 4.52 | No | No |
| Lopresti^48^ | 2019 | N/A | Moderate (2) | 9 | 1 | 7 | 1.32 | No | No |
| Kris-Etherton *et al.*^49^ | 2020 | Moderate (2) | Moderate (2) | 19 | 0 | 8 | 5.78 | No | Yes |
| Taylor *et al.*^50^ | 2020 | Moderate (2) | Weak (1) | 17 | 21 | 64 | 3.76 | No | No |
| Average (SD) | 2015 (4) | 2.29 (0.64) | 2.08 (0.7) | 11 (7) | 62 (70) | 98 (204) | 4.90 (4.35) | No (58%) | No 12 (54%) |
| *Note.* Yes and no categories were coded as 1 and 0, respectively. *Abbreviations*. IF, Impact Factor; ISNPR, International Society of Nutritional Psychiatry Research; N/K, not known; PFCOI, potential financial conflict of interest.  ^a^ Estimated empirical papers specifically underlying the conclusion on the potential association between diet and depression (in accordance with our inclusion criteria).  ^b^ Number of citations to the study (<https://www.scholar.google.com>), June 3-8, 2020.  ^c^ Weighted count of all online attention (<https://www.altmetric.com>), June 3-8, 2020.  ^d^ Impact Factor of the Journal in which the study is published (<https://www.webofknowledge.com>). | | | | | | | | | |

|  | **Table N.** Spearman’s ρ (rho) rank correlation coefficients among study types, conclusions, year of publication, number of input papers, various indicators of impact, and potential conflicts of interest | | | | | | | | | | |
| --- | --- | --- | --- | --- | --- | --- | --- | --- | --- | --- | --- |
|  | | Study type ^a^ | Conclusion: Abstract ^b^ | Conclusion: Discussion ^b^ | Year | # citations | # input papers | Impact factor | Altmetric score | PFCOI | ISNPR member |
| Study type | | 1.000 | -0.51^**^ | -0.29^*^ | 0.18 | 0.20 | 0.41^**^ | 0.10 | 0.31^*^ | -0.21 | -0.36^*^ |
| Conclusion: Abstract | | -0.51^**^ | 1.00 | 0.85^**^ | 0.07 | -0.23 | -0.09 | -0.08 | -0.09 | 0.24 | 0.34^*^ |
| Conclusion: Discussion | | -0.29^*^ | 0.85^**^ | 1.00 | 0.01 | -0.13 | -0.01 | -0.06 | 0.05 | 0.26 | 0.31^*^ |
| Year | | 0.18 | 0.07 | 0.01 | 1.00 | -0.71^**^ | 0.28^**^ | -0.02 | 0.09 | -0.16 | -0.25 |
| # citations | | 0.20 | -0.23 | -0.13 | -0.71^**^ | 1.00 | 0.61 | 0.30^*^ | 0.61^**^ | 0.27 | 0.32^*^ |
| # input papers | | 0.41^**^ | -0.09 | -0.01 | 0.28^*^ | 0.06 | 1.00 | 0.14 | 0.21 | 0.09 | -0.01 |
| Impact factor | | 0.10 | -0.08 | -0.06 | -0.02 | 0.30^*^ | 0.14 | 1.00 | 0.44^**^ | 0.12 | 0.31^*^ |
| Altmetric score | | 0.31^*^ | -0.09 | 0.05 | -0.09 | 0.61^**^ | 0.21 | 0.44^**^ | 1.00 | 0.22 | 0.32^*^ |
| PFCOI | | -0.21 | 0.24 | 0.26 | -0.16 | 0.27 | 0.09 | 0.12 | 0.22 | 1.00 | 0.56^**^ |
| ISNPR member | | -0.36^*^ | 0.34^*^ | 0.31^*^ | -0.25 | 0.32^*^ | -0.01 | 0.31^*^ | 0.32^*^ | 0.56^**^ | 1.00 |
|  | *Abbreviations.* ISNPR, International Society of Nutritional Psychiatry Research; PFCOI, potential financial conflict of interest.  ^a^ Study type coded as: 1 = narrative review, 2 = systematic review, 3 = meta-analysis.  ^b^ Conclusion coded as: 1 = weak, 2 = moderate, 3 = strong. | | | | | | | | | | |
|  | * *P* < 0.05, ** *P* < 0.01 one-sided. | | | | | | | | | | |

| **Table O.** Distribution of potential conflicts of interest per study types and conclusions | | | | | | |
| --- | --- | --- | --- | --- | --- | --- |
|  |  | **INSPR member ^a^** | **No ISNPR member** | **Industry funding ^b^** | **No industry funding** |  |
| **Study type** | Meta-analyses | 1 (7.1%) | 13 (92.9%) | 0 (0%) | 13 (100%) |  |
|  | Systematic reviews | 3 (25%) | 9 (75%) | 4 (33.3%) | 8 (66.6%) |  |
|  | Narrative reviews | 11 (45.8%) | 13 (54.2%) | 4 (22.2%) | 14 (77.8%) |  |
| **Conclusion: Abstract** | Strong | 5 (55.6%) | 4 (44.4%) | 2 (25%) | 6 (75%) |  |
|  | Moderate | 9 (34.6%) | 17 (65.4%) | 6 (26.1%) | 17 (73.9%) |  |
|  | Weak | 1 (8.3%) | 11 (91.7%) | 0 (0%) | 11 (100%) |  |
| **Conclusion: Discussion** | Strong  Moderate  Weak | 4 (50%)  10 (34.5%)  1 (7.7%) | 4 (50%)  19 (65.5%)  12 (92.3%) | 2 (28.6%)  6 (24%)  0 (0%) | 5 (71.4%)  19 (76%)  11 (100%) |  |
| *Note.* Parentheses show percentages of study types and conclusions within INSPR membership or food industry funding.  *^a^* INSPR members wrote 30% of all included articles on the topic of diet and depression.  ^b^ Industry-funded authors wrote 19% of all included articles on the topic of diet and depression. | | | | | | |

References

1 Psaltopoulou T, Sergentanis TN, Panagiotakos DB, Sergentanis IN, Kosti R, Scarmeas N. Mediterranean diet, stroke, cognitive impairment, and depression: A meta-analysis. *Ann Neurol* 2013; **74**: 580–591.

2 Lai JS, Hiles S, Bisquera A, Hure AJ, McEvoy M, Attia J. A systematic review and meta-analysis of dietary patterns and depression in community-dwelling adults. *Am J Clin Nutr* 2014; **99**: 181–97.

3 Rahe C, Unrath M, Berger K. Dietary patterns and the risk of depression in adults: A systematic review of observational studies. *Eur J Nutr* 2014; **53**: 997–1013.

4 Li F, Liu X, Zhang D. Fish consumption and risk of depression: A meta-analysis. *J Epidemiol Community Health* 2015; **70**: 299–304.

5 Grosso G, Micek A, Marventano S, Castellano S, Mistretta A, Pajak A *et al.* Dietary n-3 PUFA, fish consumption and depression: A systematic review and meta-analysis of observational studies. *J Affect Disord* 2016; **205**: 269–281.

6 Liu X, Yan Y, Li F, Zhang D. Fruit and vegetable consumption and the risk of depression: A meta-analysis. *Nutrition* 2016; **32**: 296–302.

7 Li Y, Lv M-R, Wei Y-J, Sun L, Zhang J-X, Zhang H-G *et al.* Dietary patterns and depression risk: A meta-analysis. *Psychiatry Res* 2017; **253**: 373–382.

8 Molendijk M, Molero P, Ortuño Sánchez-Pedreño F, Van der Does W, Angel Martínez-González M. Diet quality and depression risk: A systematic review and dose-response meta-analysis of prospective studies. *J Affect Disord* 2018; **226**: 346–354.

9 Saghafian F, Malmir H, Saneei P, Milajerdi A, Larijani B, Esmaillzadeh A. Fruit and vegetable consumption and risk of depression: Accumulative evidence from an updated systematic review and meta-Analysis of epidemiological studies. *Br J Nutr* 2018; **119**: 1087–1101.

10 Yang Y, Kim Y, Je Y. Fish consumption and risk of depression: Epidemiological evidence from prospective studies. *Asia-Pacific Psychiatry* 2018; **10**: e12335.

11 Lassale C, Batty GD, Baghdadli A, Jacka F, Sánchez-Villegas A, Kivimäki M *et al.* Healthy dietary indices and risk of depressive outcomes: a systematic review and meta-analysis of observational studies. *Mol Psychiatry* 2019; **24**: 965–986.

12 Nicolaou M, Colpo M, Vermeulen E. Association of a priori dietary patterns with depressive symptoms: a harmonized meta-analysis of observational studies. *Psychol Med* 2019.in press.

13 Salari-Moghaddam A, Saneei P, Larijani B, Esmaillzadeh A. Glycemic index, glycemic load, and depression: a systematic review and meta-analysis. *Eur J Clin Nutr* 2019; **73**: 356–365.

14 Shafiei F, Salari-Moghaddam A, Larijani B, Esmaillzadeh A. Adherence to the mediterranean diet and risk of depression: A systematic review and updated meta-analysis of observational studies. *Nutr Rev* 2019; **77**: 230–239.

15 Murakami K, Sasaki S. Dietary intake and depressive symptoms: A systematic review of observational studies. *Mol Nutr Food Res* 2010; **54**: 471–488.

16 Quirk SE, Williams LJ, O’Neil A, Pasco JA, Jacka FN, Housden S *et al.* The association between diet quality, dietary patterns and depression in adults: a systematic review. *BMC Psychiatry* 2013; **13**: 175.

17 Sanhueza C, Ryan L, Foxcroft DR. Diet and the risk of unipolar depression in adults: Systematic review of cohort studies. *J Hum Nutr Diet* 2013; **26**: 56–70.

18 O’Neil A, Quirk SE, Housden S, Brennan SL, Williams LJ, Pasco JA *et al.* Relationship between diet and mental health in children and adolescents: A systematic review. *Am J Public Health* 2014; **104**: e31–e42.

19 Opie RS, O’Neil A, Itsiopoulos C, Jacka FN. The impact of whole-of-diet interventions on depression and anxiety: A systematic review of randomised controlled trials. *Public Health Nutr* 2015; **18**: 2074–2093.

20 Khalid S, Williams CM, Reynolds SA. Is there an association between diet and depression in children and adolescents? A systematic review. *Br J Nutr* 2016; **116**: 2097–2108.

21 Rahimlou M, Morshedzadeh N, Karimi S, Jafarirad S. Association between dietary glycemic index and glycemic load with depression: a systematic review. *Eur J Nutr* 2018; **57**: 2333–2340.

22 Altun A, Brown H, Szoeke C, Goodwill AM. The Mediterranean dietary pattern and depression risk: A systematic review. *Neurol Psychiatry Brain Res* 2019; **33**: 1–10.

23 Arab A, Mehrabani S, Moradi S, Amani R. The association between diet and mood: A systematic review of current literature. *Psychiatry Res* 2019; **271**: 428–437.

24 Tuck N-J, Farrow C, Thomas JM. Assessing the effects of vegetable consumption on the psychological health of healthy adults: a systematic review of prospective research. *Am J Clin Nutr* 2019; **110**: 196–211.

25 Głąbska D, Guzek D, Groele B, Gutkowska K. Fruit and Vegetable Intake and Mental Health in Adults: A Systematic Review. *Nutrients* 2020; **12**: 115.

26 Ljungberg T, Bondza E, Lethin C. Evidence of the Importance of Dietary Habits Regarding Depressive Symptoms and Depression. *Int J Environ Res Public Health* 2020; **17**: 1616.

27 McGrath-Hanna NK, Greene DM, Tavernier RJ, Bult-Ito A. Diet and mental health in the Arctic: is diet an important risk factor for mental health in circumpolar peoples? - a review. *Int J Circumpolar Health* 2003; **62**: 228–241.

28 Bamber DJ, Stokes CS, Stephen AM. The role of diet in the prevention and management of adolescent depression. *Nutr Bull* 2007; **32**: 90–99.

29 Low Dog T. The role of nutrition in mental health. *Altern Ther Health Med* 2010; **16**: 42–46.

30 Jacka FN, Berk M. Depression, diet and exercise. *Med J Aust* 2012; **199**: S21–S23.

31 Jacka FN, Mykletun A, Berk M. Moving towards a population health approach to the primary prevention of common mental disorders. *BMC Med* 2012; **10**: 149.

32 Berk M, Sarris J, Coulson CE, Jacka FN. Lifestyle management of unipolar depression. *Acta Psychiatr Scand* 2013; **127**: 38–54.

33 Manosso LM, Moretti M, Rodrigues ALS. Nutritional strategies for dealing with depression. *Food Funct* 2013; **4**: 1776–1793.

34 Sanchez-Villegas A, Martínez-González MA. Diet, a new target to prevent depression? *BMC Med* 2013; **11**: 3.

35 Sarris J, O’Neil A, Coulson CE, Schweitzer I, Berk M. Lifestyle medicine for depression. *BMC Psychiatry* 2014; **14**: 1–13.

36 Lachance L, Ramsey D. Food, mood, and brain health: implications for the modern clinician. *Mo Med* 2015; **112**: 111–115.

37 Lang UE, Beglinger C, Schweinfurth N, Walter M, Borgwardt S. Nutritional aspects of depression. *Cell Physiol Biochem* 2015; **37**: 1029–1043.

38 Sarris J, Logan AC, Akbaraly TN, Amminger GP, Balanzá-Martínez V, Freeman MP *et al.* Nutritional medicine as mainstream in psychiatry. *The Lancet Psychiatry* 2015; **2**: 271–274.

39 Jacka FN. Nutritional Psychiatry: Where to Next? *EBioMedicine* 2017; **17**: 24–29.

40 Libuda L, Antel J, Hebebrand J, Föcker M. Nutrition and mental diseases: Focus depressive disorders. *Nervenarzt* 2017; **88**: 87–101.

41 Opie RS, Itsiopoulos C, Parletta N, Sanchez-Villegas A, Akbaraly TN, Ruusunen A *et al.* Dietary recommendations for the prevention of depression. *Nutr Neurosci* 2017; **20**: 161–171.

42 Stevenson RJ. Psychological correlates of habitual diet in healthy adults. *Psychol Bull* 2017; **143**: 53–90.

43 Brietzke E, Mansur RB, Subramaniapillai M, Banlanzá-Martínez V, Vinberg M, González-Pinto A *et al.* Ketogenic diet as a metabolic therapy for mood disorders: Evidence and developments. *Neurosci Biobehav Rev* 2018; **94**: 11–16.

44 Mörkl S, Wagner-Skacel J, Lahousen T, Lackner S, Holasek SJ, Bengesser SA *et al.* The Role of Nutrition and the Gut-Brain Axis in Psychiatry: A Review of the Literature. *Neuropsychobiology* 2018; : 1–9.

45 Perez L. The Role of Dietary Patterns in Mood Disorders: Prospective Research in Youth Populations. *Am J Lifestyle Med* 2018; **12**: 286–290.

46 Hosker DK, Elkins RM, Potter MP. Promoting Mental Health and Wellness in Youth Through Physical Activity, Nutrition, and Sleep. *Child Adolesc Psychiatr Clin N Am* 2019; **28**: 171–193.

47 Huang Q, Liu H, Suzuki K, Ma S, Liu C. Linking What We Eat to Our Mood: A Review of Diet, Dietary Antioxidants, and Depression. *Antioxidants* 2019; **8**: 376.

48 Lopresti AL. It is time to investigate integrative approaches to enhance treatment outcomes for depression? *Med Hypotheses* 2019; **126**: 82–94.

49 Kris-Etherton PM, Petersen KS, Hibbeln JR, Hurley D, Kolick V, Peoples S *et al.* Nutrition and behavioral health disorders: depression and anxiety. *Nutr Rev* 2020; **00**: 1–14.

50 Taylor AM, Holscher HD. A review of dietary and microbial connections to depression, anxiety, and stress. *Nutr Neurosci* 2020; **23**: 237–250.
