# Supplementary material for: Impact of review method on the conclusions of clinical reviews: A systematic review on dietary interventions in depression as a case in point
Source: PLoS One. 2020 Sep 16;15(9):e0238131. doi: 10.1371/journal.pone.0238131 (PMC7494108; doi:10.1371/journal.pone.0238131)
Supplement: S1 Appendix — (DOCX) [file pone.0238131.s009.docx]

GRADE evaluation of three patient-relevant health care questions

**1. Question: Should a healthy diet (vs. no dietary change) be used to prevent depression in people at risk?**

| **Certainty assessment** | | | | | | | **№ of patients** | | **Effect** | | **Certainty** | **Plain language summary** |
| --- | --- | --- | --- | --- | --- | --- | --- | --- | --- | --- | --- | --- |
| **№ of studies** | **Study design** | **Risk of bias** | **Inconsistency** | **Indirectness** | **Imprecision** | **Other considerations** | **a healthy diet** | **no dietary change** | **Relative (95% CI)** | **Absolute (95% CI)** |  |  |
| **Depressive symptoms (follow up: range 10 days to 12 months; assessed with: Self-report measures)** | | | | | | | | | | | | |
| 2 | randomized trials | serious ^a^ | not serious | not serious | serious ^b^ | none | 512 | 513 | - | Hedges' g **0.06 SD higher** (0.10 lower to 0.22 higher)* | ⨁⨁◯◯ LOW | The evidence suggests that a healthy diet may not prevent depressive symptoms. |

**CI:** Confidence interval; **RR:** Risk ratio; **MD:** Mean difference. ^*^Results stem from our performed meta-analysis of RCTs (see S8 Text).

**Explanations**

a. Unclear or no blinding of participants, investigators, and outcome assessors; loss-to-follow-up ranged from 0 - 23%; use of invalid outcome measures (all RCTs were unblinded and all outcome measures were self-reported; depression rating scales include items that may capture changes in symptoms of metabolic diseases, like fatigue or weight gain, instead of changes in severity of depression [1]).

b. The CI includes a potential benefit and harm.

**2. Question: Should a dietary intervention (vs. a non-dietary intervention) be used to treat people with (sub-)clinical depression?**

| **Certainty assessment** | | | | | | | **№ of patients** | | **Effect** | | **Certainty** | **Plain language summary** |
| --- | --- | --- | --- | --- | --- | --- | --- | --- | --- | --- | --- | --- |
| **№ of studies** | **Study design** | **Risk of bias** | **Inconsistency** | **Indirectness** | **Imprecision** | **Other considerations** | **a dietary intervention** | **a non-dietary intervention** | **Relative (95% CI)** | **Absolute (95% CI)** |  |  |
| **Depressive symptoms (follow up: range 12 weeks to 24 months; assessed with: Self-report measures)** | | | | | | | | | | | | |
| 4 | randomized trials | very serious^a^ | not serious | not serious | serious ^b^ | none | 115 | 101 | - | Hedges' g **0.27 SD lower** (0.66 lower to 0.13 higher)* | ⨁◯◯◯ VERY LOW | The evidence is very uncertain about the treatment effect of a dietary intervention on depressive symptoms. |

**CI:** Confidence interval; **RR:** Risk ratio; **MD:** Mean difference. ^*^Results stem from our performed meta-analysis of RCTs (see S8 Text).

**Explanations**

a. Unclear or no blinding of participants, investigators, and outcome assessors; unclear allocation concealment; loss-to-follow-up ranged from 13 - 51% with higher rates in the control condition; use of invalid outcome measures (no blinding of participants to conditions, expectancy was selectively inducted in the dietary arm, while outcome measures were self-reported [2]; depression rating scales include items that may capture in symptoms of metabolic diseases, like fatigue or weight gain, instead of changes in severity of depression [1]).

b. The CI includes a potential appreciable benefit and harm.

**3. Question: Should a dietary intervention (vs. no intervention or control) be used to reduce depressive symptoms in the general population?**

| **Certainty assessment** | | | | | | | **№ of patients** | | **Effect** | | **Certainty** | **Plain language summary** |
| --- | --- | --- | --- | --- | --- | --- | --- | --- | --- | --- | --- | --- |
| **№ of studies** | **Study design** | **Risk of bias** | **Inconsistency** | **Indirectness** | **Imprecision** | **Other considerations** | **a dietary intervention** | **no intervention or control** | **Relative (95% CI)** | **Absolute (95% CI)** |  |  |
| **Depressive symptoms (follow up: range 12 weeks to 24 months; assessed with: Self-report measures)** | | | | | | | | | | | | |
| 12 | randomized trials | very serious^a^ | serious ^b^ | not serious | not serious | none | 18622 | 26877 | - | Hedges' g **0.14 SD lower** (0.24 lower to 0.04 lower)* | ⨁◯◯◯ VERY LOW | The evidence is very uncertain about the effect of a dietary intervention on depressive symptoms. |
| **Major adverse cardiovascular events (follow up: range 27 months to 4.8 years)^1¶^** | | | | | | | | | | | | |
| 2 | randomized trials | serious ^c^ | serious ^d^ | not serious | not serious | none | 187/5299 (3.5%) | 145/2753 (5.3%) | **RR 0.69** (0.55 to 0.86) | **16 fewer per 1,000** (from 24 fewer to 7 fewer) | ⨁⨁◯◯ LOW | The evidence suggests a dietary intervention may result in a slight reduction in major adverse cardiovascular events. |
| **Weight loss (follow up: range 6 months to 5 years; assessed with: kg)^2¶^** | | | | | | | | | | | | |
| 7 | randomized trials | serious ^e^ | serious ^b^ | not serious | not serious | none | 1848 | 1588 | - | MD **1.75 kg lower** (2.86 lower to 0.64 lower) | ⨁⨁◯◯ LOW | The evidence suggests a dietary intervention may result in a slight reduction in weight loss. |
| **Systolic blood pressure (follow up: 24 months)^3¶^** | | | | | | | | | | | | |
| 6 | randomized trials | serious ^f^ | serious ^b^ | not serious | not serious ^g^ | none | 3994* | 3993* | - | MD **1.44 mmHg lower** (2.88 lower to 0.01 higher) | ⨁⨁◯◯ LOW | The evidence suggests that a dietary intervention may result in little to no difference in systolic blood pressure. |
| **Diastolic blood pressure (follow up: mean 24 months)^3¶^** | | | | | | | | | | | | |
| 6 | randomized trials | serious ^f^ | serious ^b^ | not serious | not serious | none | 3994* | 3993* | - | MD **0.7 mmHg lower** (1.34 lower to 0.07 lower) | ⨁⨁◯◯ LOW | The evidence suggests a dietary intervention may result in a slight reduction in diastolic blood pressure. |
| **Type II diabetes mellitus (follow up: range 3.2 years to 20 years)^4¶^** | | | | | | | | | | | | |
| 8 | observational studies | serious ^h^ | serious ^b, d^ | not serious | not serious | none | 59635* | 59635* | - | RR **0.83 higher** (0.74 higher to 0.92 higher) | ⨁◯◯◯ VERY LOW | The evidence is very uncertain about the effect of a dietary intervention on type II diabetes mellitus. |

**CI:** Confidence interval; **RR:** Risk ratio; **MD:** Mean difference. ^*^Results stem from our performed meta-analysis of RCTs (see S8 Text).

* numbers reflect the total number of participants divided by two as no information was provided about the number of participants per condition.

**^¶^** We also applied the GRADE framework on existing meta-analyses of RCTs on health-related outcomes other than depression (identified through non-systematic searches) to obtain a first reference on how certain the evidence is regarding the benefits of a diet on major cardiovascular events, weight loss, blood pressure, and type II diabetes.

**Explanations**

a. Unclear or no blinding of participants, investigators, and outcome assessors; unclear allocation concealment; unknown loss-to-follow-up rates; selective outcome reporting bias; use of invalid outcome measures (all RCTs were unblinded and all outcome measures were self-reported; depression rating scales include items that may capture changes in symptoms of metabolic diseases, like fatigue or weight gain, instead of changes in severity of depression [1]).

b. Significant unexplained statistical heterogeneity.

c. Unclear or no blinding of participants, investigators, and outcome assessors; unclear allocation concealment.

d. Wide variance of point estimates across studies; no overlap of confidence intervals.

e. Unclear or no blinding of participants, investigators, and outcome assessors; loss-to-follow-up ranged from 0 - 80% with higher rates in the control condition.

f. Unclear or no blinding of participants; unclear attrition rates.

g. Although the CI includes no effect, it includes a potential appreciable benefit.

h. Unclear or no lack of blinding; self-reporting bias for outcomes; measurement error for dietary intake.

**References *(for an AMSTAR assessment, see Table P below)***

^1^ Liyanage *et al.* (2016) [3]

^2^ Esposito *et al.* (2011) [4]

^3^ Nissensohn *et al.* (2016) [5]

^4^ Schwingshackl *et al.* (2015) [6]

| **Table P.** AMSTAR scores for meta-analyses of RCTs regarding other health-related outcomes | | | | | | | | | | | | | | | | | | | |
| --- | --- | --- | --- | --- | --- | --- | --- | --- | --- | --- | --- | --- | --- | --- | --- | --- | --- | --- | --- |
| **Author** | **Outcome** | **I1** | **I2** | **I3** | **I4** | **I5** | **I6** | **I7** | **I8** | **I9** | **I10** | **I11** | **I12** | **I13** | **I14** | **I15** | **I16** | **AMSTAR score** |  |
| Esposito *et al.* (2011) [4] | Weight loss | N | N | Y | P | Y | N | N | Y | P | N | Y | N | N | Y | Y | Y | Critically low |  |
| Schwingshackl *et al.* (2014) [6] | Diabetes | Y | N | Y | P | Y | Y | Y | Y | P | N | Y | Y | Y | Y | Y | Y | Low |  |
| Liyanage *et al.* ﻿(2016) [3] | Major cardiovascular events | Y | Y | Y | P | Y | Y | Y | Y | Y | N | Y | Y | Y | N | N | Y | Low |  |
| Nissensohn *et al.* (2016) [5] | Hypertension | Y | N | Y | P | Y | N | N | P | Y | Y | Y | N | Y | Y | N | N | Critically low |  |
| *Note.* Critical items are highlighted in grey. *Abbreviations:* I, item number; N, no; P, partial; Y, yes. *Items:* I1 = Did the research questions and inclusion criteria for the review include the components of PICO? ﻿I2 = Did the report of the review contain an explicit statement that the review methods were established prior to the conduct of the review and did the report justify any significant deviations from the protocol? ﻿I3 = Did the review authors explain their selection of the study designs for inclusion in the review? ﻿I4 = Did the review authors use a comprehensive literature search strategy? ﻿I5 = Did the review authors perform study selection in duplicate? ﻿I6 = Did the review authors perform data extraction in duplicate? ﻿I7 = Did the review authors provide a list of excluded studies and justify the exclusions? ﻿I8 = Did the review authors describe the included studies in adequate detail? ﻿I9 = Did the review authors use a satisfactory technique for assessing the risk of bias (RoB) in individual studies that were included in the review? ﻿I10 = Did the review authors report on the sources of funding for the studies included in the review? ﻿I11 = If meta-analysis was performed did the review authors use appropriate methods for statistical combination of results? ﻿I12 = If meta-analysis was performed, did the review authors assess the potential impact of RoB in individual studies on the results of the meta-analysis or other evidence synthesis? ﻿I13 = Did the review authors account for RoB in individual studies when interpreting/ discussing the results of the review? ﻿I14 = Did the review authors provide a satisfactory explanation for, and discussion of, any heterogeneity observed in the results of the review? ﻿I15 = If they performed quantitative synthesis did the review authors carry out an adequate investigation of publication bias (small study bias) and discuss its likely impact on the results of the review? ﻿I16 = Did the review authors report any potential sources of conflict of interest, including any funding they received for conducting the review? | | | | | | | | | | | | | | | | | | | |

References

1. Molendijk M, Molero P, Ortuño Sánchez-Pedreño F, Van der Does W, Angel Martínez-González M. Diet quality and depression risk: A systematic review and dose-response meta-analysis of prospective studies. J Affect Disord. 2018;226: 346–354. doi:10.1016/j.jad.2017.09.022

2. Molendijk ML, Fried EI, Van der Does W. The SMILES trial: do undisclosed recruitment practices explain the remarkably large effect? BMC Med. 2018;16: 243. doi:10.1186/s12916-018-1221-5

3. Liyanage T, Ninomiya T, Wang A, Neal B, Jun M, Wong MG, et al. Effects of the mediterranean diet on cardiovascular outcomes-a systematic review and meta-analysis. PLoS ONE. 2016. doi:10.1371/journal.pone.0159252

4. Esposito K, Kastorini CM, Panagiotakos DB, Giugliano D. Mediterranean diet and weight loss: Meta-analysis of randomized controlled trials. Metab Syndr Relat Disord. 2011;9: 1–12. doi:10.1089/met.2010.0031

5. Nissensohn M, Román-Viñas B, Sánchez-Villegas A, Piscopo S, Serra-Majem L. The Effect of the Mediterranean Diet on Hypertension: A Systematic Review and Meta-Analysis. J Nutr Educ Behav. 2016;48: 42-53.e1. doi:10.1016/j.jneb.2015.08.023

6. Schwingshackl L, Missbach B, König J, Hoffmann G. Adherence to a Mediterranean diet and risk of diabetes: A systematic review and meta-analysis. Public Health Nutr. 2015;18: 1292–1299. doi:10.1017/S1368980014001542
