# Supplementary material for: Impact of review method on the conclusions of clinical reviews: A systematic review on dietary interventions in depression as a case in point
Source: PLoS One. 2020 Sep 16;15(9):e0238131. doi: 10.1371/journal.pone.0238131 (PMC7494108; doi:10.1371/journal.pone.0238131)
Supplement: Attachment — Submitted filename: Response to reviewers.docx [file pone.0238131.s010.docx]

**Reviewer 1**

We thank reviewer 1 for the extensive feedback about the objective, introduction, methods, and results. We incorporated most feedback. Based on that we think our paper improved to a great extent. It also became more comprehensible for the reader. Thanks for that.

**General remarks**

**Comment # 1**

Some sentences need revision in order to be correct (e.g. past /present tenses; and some words have missing letters).

**Thomas-Odenthal and colleagues reply**

We agree that some errors were in the text. We corrected those errors.

**Comments about abstract**

**Comment # 2**

I suggest to be **more precise** in the objective of the work presented and revise which aspects are important to be mentioned in the abstract (and which are not so important). I think that the abstract needs more structure to present the main focus of the manuscript.

**Thomas-Odenthal and colleagues reply**

We agree and improved the abstract by focusing more on the conclusion strength and study type association instead of the biases that may underlie such conclusions.

**Comments about introduction**

**Comment # 3**

Authors should be sure that all statements in the introduction are referenced (e.g. first 4 sentences of the introduction need to be referenced).

**Thomas-Odenthal and colleagues reply**

We agree and added the references for the first four sentences.

**Comment # 4**

The objective of the present review is to investigate the difference between the conclusions given in the different types of reviews (narrative reviews, systematic reviews (with/and without meta-analysis)) regarding diet and depression. In this sense, I would suggest authors to rephrase the introduction, in order to back up their objective. For example, they could mention which kind of literature is found for diet and depression; give an overview (and definition) of (narrative review vs. systematic review with/without meta-analysis); and how the conclusion of each type of review could influence practice.

**Thomas-Odenthal and colleagues reply**

We agree that the introduction would benefit from a clearer structure. We gave examples for a narrative review and a meta-analysis and a broader statement that based on which conclusions may be adopted by clinicians, it may alter the clinical implementations (line 80-81). Furthermore, we think that we already gave an overview of and definition about each review type (systematic reviews: line 57-61, narrative reviews: line 88) and for systematic reviews also in the context of diet and depression research (line: 61-72).

**Comment # 5**

Recommendations and guidelines do not have to rely on RCTs. It is correct, that the best evidence usually stems from RCTs, because they can investigate causality. This does not imply that standards of conduct are high: There are RCTs with low-quality standards, and also non-RCTs with high-quality standards.

**Thomas-Odenthal and colleagues reply**

We agree that recommendations do not ultimately have to rely on RCTs, especially if no well-conducted RCTs exist. If multiple well-conducted observational studies exist, particularly prospective cohort studies, they should indeed be preferred over low-quality RCTs in guideline development, optimally pooled in a meta-analysis. In diet and depression research, only a few RCTs exist while the majority of evidence stems from observational studies. The problem is however that the research is weak for both RCTs and observational studies and therefore prone to bias and confounders [1]. Moreover, our review shows that most meta-analyses, which are often used for guideline development, are of critically low quality, too. Hence, we argue that the best available evidence still comes from multiple RCTs although overall all evidence is weak.

**Comment # 6**

There are some research questions which cannot be answered with RCTs (which is often the case in nutritional topics), thus, guidelines have to be done with non RCTs. Suggest authors to revise some literature about evidence and study designs and rephrase this section.

**Thomas-Odenthal and colleagues reply**

We agree with the reasoning behind the idea that guidelines should be done with well-conducted non-RCTs if risks of diseases are studied, or no well-conducted RCTs exist [2]. However, in diet and depression research, no such observational studies exist because reversed causation, undetected biases, and residual confounding may underlie such relationships [1,3–5]. Hence, the relationship between diet and depression is not clear. In our opinion, guidelines should not rely on weak evidence. Yet, many researchers do exactly this, which we try to show with our research.

Moreover, it is often argued that with lower-certainty evidence but high benefit:risk ratio, as in nutritional interventions, the dietary recommendations may be reasonable to implement [2]. We argue that this is not true for diet and depression research because such conclusions create the impression that patients are responsible for their mental illness because of their bad dietary habits, which may worsen disease progression. Hence, the evidence should be clear when making such recommendations, which we think is not the case. Better research on this topic is needed.

**Comment # 7**

Recommend to revise the following terms used: systematic review and meta-analysis. They are not strictly different type of reviews. A systematic review can include a meta-analysis, but does not necessary have to include one. My recommendation would be to use: narrative reviews, systematic reviews without meta-analysis, and systematic reviews with meta-analysis.

**Thomas-Odenthal and colleagues reply**

We agree with the reviewer that leaving meta-analyses under the term of systematic reviews makes the distinction between the two more comprehensible. Hence, we changed these two terms into systematic reviews with and without meta-analysis if we think the flow of the text would improve. We also kept the old distinction “meta-analysis” and “systematic review”. We think that the reader will understand.

**Comments about search strategy**

**Comment # 8**

Suggest to include the date of registration in PROSPERO (also in the supplemental material where the registration form is presented).

**Thomas-Odenthal and colleagues reply**

We added this detail.

**Comment # 9**

Please explain what OSF means.

**Thomas-Odenthal and colleagues reply**

We agree that this term can be specified. We already explained that it is a preregistration platform but also added this detail in parentheses: “a free, open-source web application”.

**Comment # 10**

Why did authors restrict your search from January 2019 until September 2019?

**Thomas-Odenthal and colleagues reply**

We began our initial search in January 2019 and continuously added papers and updated our dataset during the review process up until September 2019. We performed a completely new search within 4 days up until 30^th^ May, 2020 (see reply below). We understand that this is not equivalent to the years considered (from inception to May 30^th^, 2020). We changed it in the manuscript accordingly.

**Comment # 11**

Did authors use only free-text search or also controlled vocabulary? In the PROSPERO registration form, authors list more databases that were searched. Which databases were included in the final version of the review?

**Thomas-Odenthal and colleagues reply**

Yes, in our initial search, we only used free-text search and no controlled vocabulary. We understand that this might have led to an incomplete dataset missing relevant papers. Hence, we conducted a completely new search with the following search terms including MeSH terms and field codes and found 9 new reviews:

PubMed: 1775 hits

(diet OR food [MeSH] OR “diet therapy”[MeSH]) AND (depressive* OR depression OR “mental health”[MeSH] OR "mental disorder*"[Title/Abstract]) AND (“systematic review”[Publication Type] OR meta-analysis[Publication Type] OR review[Publication Type])

Web of Science Core Collection: 2248 hits

(TS=(diet OR food) AND TS=("depressive*" OR depression OR "mental health" OR "mental disorder*") AND TS=("systematic review" OR meta-analysis OR review)) AND DOCUMENT TYPES: (Review)

Indexes=SCI-EXPANDED, SSCI, A&HCI, ESCI Timespan=All years

Cochrane reviews: 55 hits

ID Search Hits

#1 ((diet OR food) AND (depression OR depressive* OR "mental disorder*" OR "mental health")):ti,ab,kw

in Cochrane Reviews (Word variations have been searched)

**Comment # 12**

In the PROSPERO registration form, authors list more databases that were searched. Which databases were included in the final version of the review?

**Thomas-Odenthal and colleagues reply**

Initially, we only searched PubMed and Web of Science. In our new search, we now systematically searched PubMed, Web of Science and Cochrane Reviews, and performed a broader, non-systematic search in google scholar.

**Comment # 13**

Suggest to include the whole search strategy as supplemental material, since the search strategy presented seems incomplete (also when opening the link provided in the PROSPERO form, the search strategy is incomplete).

**Thomas-Odenthal and colleagues reply**

We admit that the full search strategy was missing. Now we included each database-specific syntax as a supplemental file.

**Comments about inclusion criteria**

**Comment # 14**

Which depressive symptoms were considered eligible?

**Thomas-Odenthal and colleagues reply**

We did not consider specific depressive symptoms. Instead, we included studies that measured the construct of depression as assessed by standardized depression scales, such as the Beck Depression Inventory or Center for Epidemiological Studies – Depression. Also, depression diagnosed by a clinician or antidepressant-use were considered eligible as depression outcome. We made this clearer under eligibility criteria by adding this statement in parentheses behind *depressive symptoms* as inclusion criteria: “as measured by self-report depression symptom scales”.

**Comments about conclusion classification**

**Comment # 15**

How did authors define the conclusion, in cases where the study reported different statements between the conclusion in the abstract section and in the main manuscript? Also, one study could report on various conclusions: how was this handled (where all conclusions extracted and rated)?

**Thomas-Odenthal and colleagues reply**

Initially, we only included the conclusion and recommendation from the abstract when they fit our inclusion criteria. If they did not fit our inclusion criteria, we extracted the conclusions from the discussion section instead. We agree that this procedure may seem arbitrary (although we think that most people only read abstracts). Based on your feedback, we now extracted all conclusion again, but this time from both the abstract and discussion section, and re-ran our analysis for abstract- and discussion derived conclusions separately. The results of these still support our main findings.

**Comment # 16**

How did authors address **missing data**?

**Thomas-Odenthal and colleagues reply**

We handled missing data as *missing* since we have the population of interest (i.e., all review papers); we did not impute any data. If no conclusion or recommendation were provided, we organized them under a new category “no conclusion/recommendation provided”. For recommendations, we performed our analysis with and without that additional category (because we think it may be a meaningful category since some papers don't provide recommendations due to weak evidence).

**Comments about quality assessment**

**Comment # 17**

Lines 149-154: I did not understand what this paragraph is describing about the method of GRADE? To what outcomes did authors assess GRADE? Did they do this to all included reviews or only to the identified meta-analyses?

**Thomas-Odenthal and colleagues reply**

We agree that this paragraph may be confusing. We assessed the certainty of evidence (using GRADE) on the effects of diet not only on depression but also on other outcomes such as major cardiovascular events, blood pressure, etc. We based this assessment on other well-conducted meta-analyses of RCTs on these topics that we found through non-systematic searches. We did this to get a first impression for future research. We agree that this does not add to our main objective. Therefore, we excluded this part from our method section to keep it concise and presented it only in the appendix.

**Comment # 18**

Did authors assess association with correlation coefficient (Spearman, Pearson)?

**Thomas-Odenthal and colleagues reply**
The significance statistics stem from ordinal logistic regression analyses. We added this detail for clarification: “Odds ratios (ORs) and their respective 95% confidence intervals (CIs) were used as the measure for effect size.”

**Comments about results**

**Comment # 19**

Suggest to start the results section more general. How many references were identified in the databases, how many screened, how many included, etc. Before presenting any results it is important to give the reader an overview about the studies/publications included, their general characteristics, etc.

**Thomas-Odenthal and colleagues reply**

We agree that the flow of the paper would benefit by first presenting the results of our main findings – the search strategy. We, therefore, placed the part about our meta-analysis and GRADE assessment at the end of the results section before presenting the results about our included reviews. We also added the general characteristics of the studies because we think it is important for the reader.

**Comment # 20**

Authors start the results describing their GRADE assessment of one meta-analysis. I do not understand how this should be understood contextualizing their objective? I think authors should first give results addressing their objective (i.e. how many narrative reviews, systematic reviews, etc. were found and what did they conclude).

**Thomas-Odenthal and colleagues reply**

We agree that the results of the included studies in our systematic review should be presented first, which we now did. In general, however, we also think that the GRADE assessment is important for our objective to get an idea about which review types provide an accurate representation of the current state of the evidence. Since we found that different types of review come to different conclusions, we feel obliged, as a next step, to give an overview on which review types should be relied on for evidence-based decision making and which types are more likely to be biased.

**Comment # 21**

Lines 196-200: Which outcomes were assessed with GRADE? The manuscript is lacking an overview about which publications were taken into consideration for the GRADE approach (this is also missing in the methods section).

**Thomas-Odenthal and colleagues reply**

We understand that we may not have explained the reason for integrating the GRADE approach well enough. The RCTs on diet and depression focuses on mainly three outcomes. First, most RCTs aimed to investigate the association between high diet quality and depressive symptoms without the intention to treat or prevent depression. At the same time, a few RCTs, in particular the more recent ones, went a step further and specifically tested whether depression could be clinically treated or prevented by a healthy diet. With the GRADE approach, we aimed to show how strong the evidence was regarding these 3 outcomes: association, treatment, prevention. Furthermore, we looked into the evidence of other health-related outcomes for depressed people, such as major cardiovascular diseases, type II diabetes, blood pressure, and weight loss. This assessment was based on different meta-analyses on RCTs, which is also a GRADE requirement. We understand this is confusing for the reader and therefore now only explain and present it in the appendix.

**Comment # 22**

Line 202: I think this is how the whole results section should start with.

**Thomas-Odenthal and colleagues reply**

We agree.

**Comment # 23**

Line 205: The study of Firth et al was excluded, but their data was used to re-run meta-analysis (described at the beginning of the results section)? This is confusing.

**Thomas-Odenthal and colleagues reply**

Initially, we wanted to use the meta-analysis of RCTs by Firth et al because it was the only meta-analysis on RCTs on this topic and one of the best meta-analyses we found overall. However, we detected that this study included a wrong effect size calculation, and one study that did not meet their own inclusion criteria. Hence, we excluded this meta-analysis from our systematic review on conclusion strength (because their conclusion is invalid). Instead, we decided to use the method of that meta-analysis method (e.g., inclusion criteria, search strategy) to re-run a corrected version because it was otherwise a good quality meta-analysis. This corrected meta-analysis we used then for our GRADE approach. We deleted this sentence in the results section to make it less confusing.

**Comment # 24**

Suggest to re-order the results section and provide all necessary information for the reader in order to understand the results. Supplementary material is vast and includes all results, but I suggest to depict the most important results also in the text.

**Thomas-Odenthal and colleagues reply**

We agree that the paper would benefit from a better structure. Hence, we moved the first paragraph of the results section to the end of the results section and included some study characteristics at the beginning of the results section.

Comments about conclusion section

**Comment # 25**

The conclusion sections is well written and has all elements needed in the conclusion section. As an option, authors could include some further limitations about the design of their investigation (e.g. search strategy)?

**Thomas-Odenthal and colleagues reply**
We are pleased that the reviewer is satisfied with our conclusion section. Since we updated our search strategy and performed a better search, we think that our search strategy is not a weakness anymore and therefore don’t need to be mentioned. We hope the reviewer agrees.

**Reviewer 2**

We thank reviewer two for the comprehensive feedback in particular about the search strategy. We believe that our paper significantly improved by incorporating the feedback. Thank you!

**Comments about methods – search strategy**

**Comment # 1**

Although the authors state they used PRISMA to report their study, the description of the search strategy is not compliant with that guideline. The authors don’t report the coverage of the databases– the period of time covered in the database when it is searched (not the period of time during which the searches were run by the investigators).

**Thomas-Odenthal and colleagues reply**

We agree that the description with the search strategy is not compliant with the PRISMA guidelines, in particular, the coverage of databases. Thus, we added the period of time covered in the databases in parentheses behind the names of the databases.

**Comment # 2**

It seems unlikely the search strategy reported is the actual search run in each database (PRISMA recommends reporting of at least one search strategy “…such that it could be repeated”), because it lacks database-specific syntax or indication of use of field codes. If this is the actual search strategy, it is not systematic or comprehensive: a) The lack of field codes suggests that, at best, the authors conducted a basic search in PubMed, while not taking advantage of the extensive controlled vocabulary (Medical Subject Headings) that should be used to ensure thorough searching.

**Thomas-Odenthal and colleagues reply**

We agree that the search strategy was incomplete and only contained a free-text search. This is why we did not include any data-base specific syntax or field codes. We also only assessed the unique records in Web of Science that were not captured by PubMed. We admit this was not a good strategy. We updated our search strategy and included relevant MeSH terms and field codes adapted to each individual database, see below. With our new search, we found 9 new eligible studies. We added the search strategy for each individual database in the supplemental material S2 Text.

PubMed: 1775 hits

(diet OR food [MeSH] OR “diet therapy”[MeSH]) AND (depressive* OR depression OR “mental health”[MeSH] OR "mental disorder*"[Title/Abstract]) AND (“systematic review”[Publication Type] OR meta-analysis[Publication Type] OR review[Publication Type])

Web of Science Core Collection: 2248 hits

(TS=(diet OR food) AND TS=("depressive*" OR depression OR "mental health" OR "mental disorder*") AND TS=("systematic review" OR meta-analysis OR review)) AND DOCUMENT TYPES: (Review)

Indexes=SCI-EXPANDED, SSCI, A&HCI, ESCI Timespan=All years

Cochrane reviews: 55 hits

ID Search Hits

#1 ((diet OR food) AND (depression OR depressive* OR "mental disorder*" OR "mental health")):ti,ab,kw

in Cochrane Reviews (Word variations have been searched)

**Comment # 3**

b) The search terms used do not appear to fully reflect “diet” as conceptualized by the authors. That is, the authors wanted to include reviews that examined the impact of consumption of food groups such as fruits, vegs, or fish on depressive symptoms. Yet the search string contains no terms about food groups.

**Thomas-Odenthal and colleagues reply**

We indeed did not include any terms that covered papers on the effect of food groups on depression. Initially, we thought these papers would be covered by the term “diet”. But we understand that this term does not entirely cover the consumption of food groups. Hence, for PubMed, we included the MeSH term “food” into our search strategy, which has food groups such as fruits, vegetables, or fish as subcategories. Web of Science and Cochrane reviews do not use MeSH terms. There, we narrowed our search by using field codes for topics for Web of Science and titles, abstracts, and keywords for Cochrane reviews.

**Comment # 4**

c) At the same time, several search terms seem inappropriate for the topic of depression: anxiety, psychosis.

**Thomas-Odenthal and colleagues reply**

Initially, we included these terms for future research projects. We didn’t see this as a problem because they are comorbid disorders of depression. Also, the inclusion of these terms made our search more comprehensive capturing both relevant papers about depression as well as others about comorbid disorders. However, we agree that for reasons of parsimony it is better to exclude these terms in our new search strategy, which we did.

**Comment # 5**

The protocol for this study indicates that Cochrane Library and Google Scholar would also be searched, but this does not appear to be the case? Please clarify if these were completed and if so, why the results are not included here. If not done, please explain why.

**Thomas-Odenthal and colleagues reply**

We admit that we did not mention to have searched Cochrane Reviews and Google scholar in our initial search. We indeed performed a systematic search using the old keywords in Cochrane Reviews but we forgot to mention this in the main manuscript. Similarly, we performed a non-systematic search in google scholar by browsing through the most recent and relevant ones. However, since this search was not performed systematically, we did not include it as a searched database. In our updated search, we included these searched databases and performed a systematic search in Cochrane Reviews and a general search in google scholar using a broad range of keywords related to reviews on the topic of diet and depression.

**Comments about methods – inclusion criteria**

**Comment # 6**

Expert opinion articles do not seem to me to be in the same category of document as reviews or meta analyses, so it is not clear why they should have been included in this study. Given their nature and typically brief format, it is expected that such documents would be selective in the studies cited and perhaps adamant in the conclusions drawn, so lumping them in with narrative reviews (which is done in the Results) might lead to bias.

**Thomas-Odenthal and colleagues reply**

Expert opinion articles may indeed be of shorter format and therefore more selective in their nature. Yet, not all expert opinions are short but come close to the length of narrative reviews [6]. Some expert opinions are cited very often and have a huge impact; hence, we think it is important to include these in our main analysis. However, we performed a sensitivity analysis with expert opinions excluded. We presented the results in the results section (line 262-265).

**Comments about results**

**Comment # 7**

The entire first paragraph here (lines 174-187) seems to be about the meta analysis the authors used to create what I will call a “gold standard” for the true or real effect of diet on depressive symptoms. I think the manuscript would flow better if this paragraph were in the Methods section.

**Thomas-Odenthal and colleagues reply**

We agree that the flow of the paper benefits by putting this paragraph at the end of the results section.

**Comment # 8**

I’m concerned about the data as reported here and in the flow chart (Fig 1). a) minor detail: OSF and PROSPERO are not bibliographic databases, so the # of records found by those searches should be included in the box used to indicate “additional articles identified through other means”

**Thomas-Odenthal and colleagues reply**

We agree that the OSF and PROSPERO databases are not bibliographic databases. Hence, we placed them under the category “additional articles identified through other means”.

**Comment # 9**

b) The initial search results, across all databases and resources, found a total of 1696 records. The flow chart indicates that, after duplicate removal, 1696 records remained. So there were no duplicates? I work in an environment where colleagues are collaborating on multiple SRs per year, and I have never heard of a systematic review where the searches produced no duplicates. That oddity needs to be explained.

**Thomas-Odenthal and colleagues reply**

We agree that the flow chart was incorrectly presented. We just reported the individual number of papers that remained left after removing the duplicates. With our updated search, we corrected this mistake and included the number of studies found before duplicates were removed.

References

1.	Molendijk M, Molero P, Ortuño Sánchez-Pedreño F, Van der Does W, Angel Martínez-González M. Diet quality and depression risk: A systematic review and dose-response meta-analysis of prospective studies. J Affect Disord. 2018;226: 346–354. doi:10.1016/j.jad.2017.09.022

2.	Blumberg J, Heaney RP, Huncharek M, Scholl T, Stampfer M, Vieth R, et al. Evidence-based criteria in the nutritional context. Nutr Rev. 2010;68: 478–484. doi:10.1111/j.1753-4887.2010.00307.x

3.	Jacka FN. Nutritional Psychiatry: Where to Next? EBioMedicine. 2017;17: 24–29. doi:10.1016/j.ebiom.2017.02.020

4.	Trepanowski JF, Ioannidis JPA. Perspective: Limiting Dependence on Nonrandomized Studies and Improving Randomized Trials in Human Nutrition Research: Why and How. Adv Nutr. 2018;9: 367–377. doi:10.1093/advances/nmy014

5.	Vandenbroucke JP. Observational Research, Randomised Trials, and Two Views of Medical Science. PLoS Med. 2008;5: e67. doi:10.1371/journal.pmed.0050067

6.	Sarris J, Logan AC, Akbaraly TN, Amminger GP, Balanzá-Martínez V, Freeman MP, et al. Nutritional medicine as mainstream in psychiatry. The Lancet Psychiatry. 2015;2: 271–274. doi:10.1016/S2215-0366(14)00051-0
